# Estimating the effect of cesarean delivery on long-term childhood health across two countries

**DOI:** 10.1371/journal.pone.0268103

**Published:** 2022-10-18

**Authors:** Ayya Keshet, Hagai Rossman, Smadar Shilo, Shiri Barbash-Hazan, Guy Amit, Maytal Bivas-Benita, Chen Yanover, Irena Girshovitz, Pinchas Akiva, Avi Ben-Haroush, Eran Hadar, Arnon Wiznitzer, Eran Segal

**Affiliations:** 1 Department of Computer Science and Applied Mathematics, Weizmann Institute of Science, Rehovot, Israel; 2 Department of Molecular Cell Biology, Weizmann Institute of Science, Rehovot, Israel; 3 Pediatric Diabetes Unit, Ruth Rappaport Children’s Hospital of Haifa, Rambam Healthcare Campus, Haifa, Israel; 4 Helen Schneider Hospital for Women, Rabin Medical Center, Petach Tikva, Israel; 5 Sackler Faculty of Medicine, Tel Aviv University, Tel Aviv, Israel; 6 KI Research Institute, Kfar Malal, Israel; Texas A&M University College Station, UNITED STATES

## Abstract

Assessing the impact of cesarean delivery (CD) on long-term childhood outcomes is challenging as conducting a randomized controlled trial is rarely feasible and inferring it from observational data may be confounded. Utilizing data from electronic health records of 737,904 births, we defined and emulated a target trial to estimate the effect of CD on predefined long-term pediatric outcomes. Causal effects were estimated using pooled logistic regression and standardized survival curves, leveraging data breadth to account for potential confounders. Diverse sensitivity analyses were performed including replication of results in an external validation set from the UK including 625,044 births. Children born in CD had an increased risk to develop asthma (10-year risk differences (95% CI) 0.64% (0.31, 0.98)), an average treatment effect of 0.10 (0.07–0.12) on body mass index (BMI) z-scores at age 5 years old and 0.92 (0.68–1.14) on the number of respiratory infection events until 5 years of age. A positive 10-year risk difference was also observed for atopy (10-year risk differences (95% CI) 0.74% (-0.06, 1.52)) and allergy 0.47% (-0.32, 1.28)). Increased risk for these outcomes was also observed in the UK cohort. Our findings add to a growing body of evidence on the long-term effects of CD on pediatric morbidity, may assist in the decision to perform CD when not medically indicated and paves the way to future research on the mechanisms underlying these effects and intervention strategies targeting them.

## Introduction

Medically indicated cesarean delivery (CD) is a lifesaving procedure for both mother and newborn, although it bears risk for short and long-term pediatric adverse health outcomes [1]. In the past decades, the incidence of CD increased annually by 4%, and is highly variable geographically, ranging from less than 5% of deliveries in southern Africa to almost 60% in some parts of Latin America [2]. This rise, which also reflects an increase in CDs without a medical or obstetric indication [3], is the result of cultural, personal and medico-legal reasons, with limited consideration to the impact that mode of delivery may have on long‐term pediatric health.

Previous studies have highlighted childhood obesity [4]; atopy, asthma, allergies, atopic dermatitis [5]; attention deficit hyperactivity disorder (ADHD), autistic spectrum disorder (ASD) [6]; autoimmune diseases [7] such as type 1 diabetes [8] as possible long-term outcomes of children born by CD. However, many of the studies were based on relatively small cohorts from a single geographic location, had a short follow-up period, did not account for possible confounders that might affect these associations and yielded diverse results in regard to the magnitude of the effect [1].

While the optimal incidence of CD is debatable [9–11], a better understanding of how delivery mode affects long-term health outcomes in children may influence health policies and decision processes of both clinicians and women. It could also serve as a basis for further research investigating the underlying biological mechanisms of these effects and possible interventions to improve pediatric outcomes of those born by CD. As conducting randomized controlled trials (RCTs) of delivery mode is rarely feasible and may be viewed as unethical, as it may increase the risk for adverse maternal and infant outcomes, we utilized state-of-the-art causal inference methods on high quality, high volume, longitudinal observational data that originates from Israel’s largest healthcare provider, to assess long-term adverse outcomes of children born by CD.

Following an appropriate study design and methodologies tailored to the use of observational data for causal analysis has shown promise to overcome some of the issues that arise when working with such data [12]. One such study design is the *Target Trial* [13] framework. In this method, the observational analysis is designed to explicitly emulate an RCT, including definitions for trial eligibility and treatment assignment with time zero of follow-up. By emulating an RCT, flaws such as selection bias and immortal time bias can be avoided [14]. Effect estimates from observational data obtained using this framework were comparable to those obtained from RCTs [12].

In accordance with this framework, we envisioned a hypothetical RCT which could estimate the causal effect of CD on pediatric outcomes, and utilized observational data to emulate it as closely as possible. Table 1 summarizes key components of this RCT versus their corresponding definition in our observational data. *Time-zero* (which can be viewed as target trial initiation), the eligibility-determining date in which treatment strategies are assigned and follow-up starts, was defined as the time of birth. Survival curves were estimated by fitting a pooled logistic regression model [15, 16], on a person-time data format. To adjust for baseline confounders we either added baseline selected variables to the logistic model (standardization) [17], or performed weighting using either IPW [18] or OW [19] (see Methods).

**Table 1:** Specification and emulation of a target trial of mode of delivery childhood health outcomes.

Protocol component	Target trial specification: hypothetical RCT	Target trial emulation: existing observational data
Eligibility criteria	1. Births between 1 January 2007 and 31 December 2018 2. Only women who were in Clalit HMO for at least 5 years prior to birth 3. Exclude women who have more than one previous CD 4. Exclude preterm births, defined as delivery prior to 37 completed gestational weeks 5. Exclude children born small for gestational age (SGA), defined as birth weight less than 2500 gr 6. Exclude multiple gestations	1. Same as for the target trial 2. We identified women with at least 5 years of documented medical history in Clalit’s registry data. 3. We excluded women with more than one previous CD in their medical history data 4. We define preterm births by premature_flag available from hospital data, or gestational age at birth below 37 weeks 5. We exclude children with birth weight less than 2500 grams 6. We exclude multiple gestations 7. We require information on mother-child linkages, delivery date, mode of delivery and neonatal sex
Treatment strategies	vaginal or cesarean deliveries	Same as for the target trial
Treatment assignment	Mothers are randomly assigned to a delivery strategy (cesarean section or vaginal delivery) at birth and will be aware of the strategy to which they have been assigned.	Mothers were classified by a delivery strategy (cesarean section or vaginal delivery) according to their birth data. Randomization was emulated by adjusting for baseline confounders.
Outcomes	➢ Asthma ➢ Overweight \ Obesity (defined by BMI z-score) ➢ Atopic dermatitis ➢ Celiac disease ➢ Allergies ➢ Autistic spectrum disorder (ASD) ➢ Attention deficit hyperactivity disorder (ADHD) ➢ Inflammatory bowel disease (IBD) ➢ Type 1 diabetes ➢ Juvenile idiopathic arthritis ➢ Respiratory infections ➢ Autoimmune diseases ➢ Atopy ➢ Death	Same as for the target trial
Follow-up	Follow-up starts at treatment assignment (birth time) and ends at loss to follow-up, 10 years after baseline, or on 31 December 2018, whichever occurs first.	Same as for the target trial
Causal contrasts	Intention-to-treat effect	Observational analog of intention-to-treat effect
Statistical analysis	Intention-to-treat effect is estimated by fitting a pooled logistic regression model	Same as for the target trial—also adding to the pooled logistic model the baseline covariates

## Results

**Fig 1:**
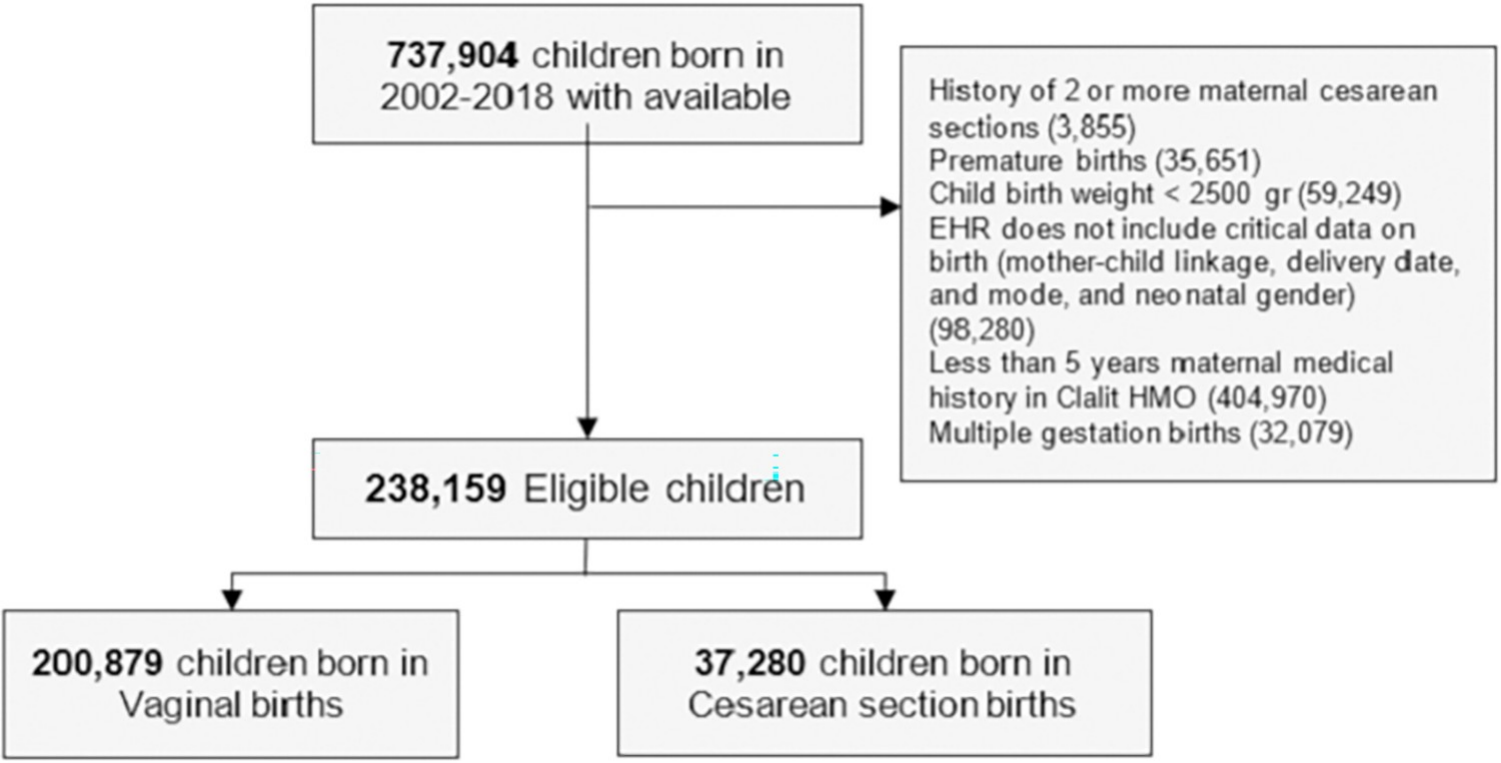
Cohort selection process. Flowchart for selection of eligible individuals from the Clalit database for emulating a target trial of birth delivery mode and childhood health outcomes. Numbers in parentheses represent unique individuals in each group. Abbreviations: EHR- Electronic health records, HMO- Health maintenance organization.

**Fig 2:**
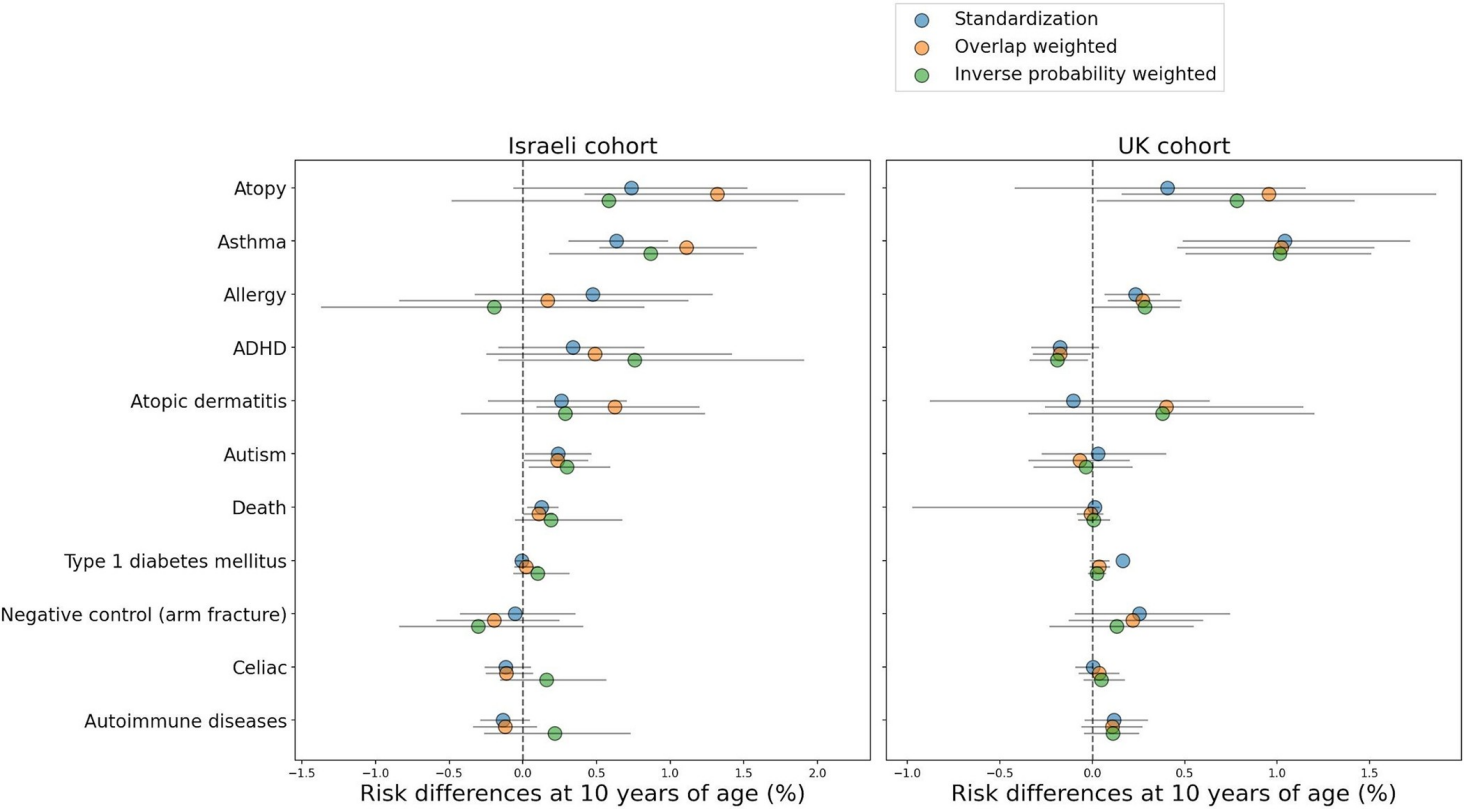
Estimated 10-yr risk difference (%) between children born vaginally or through CD. Results are shown in the Israeli cohort (Left) and UK cohort (Right), when using standardization (blue circles), weighting with overlap weights (orange circles) and weighting with Inverse probability weighting (green circles), sorted by standardized risk differences in the Israel cohort. Black lines represent 95% confidence intervals.

**Fig 3:**
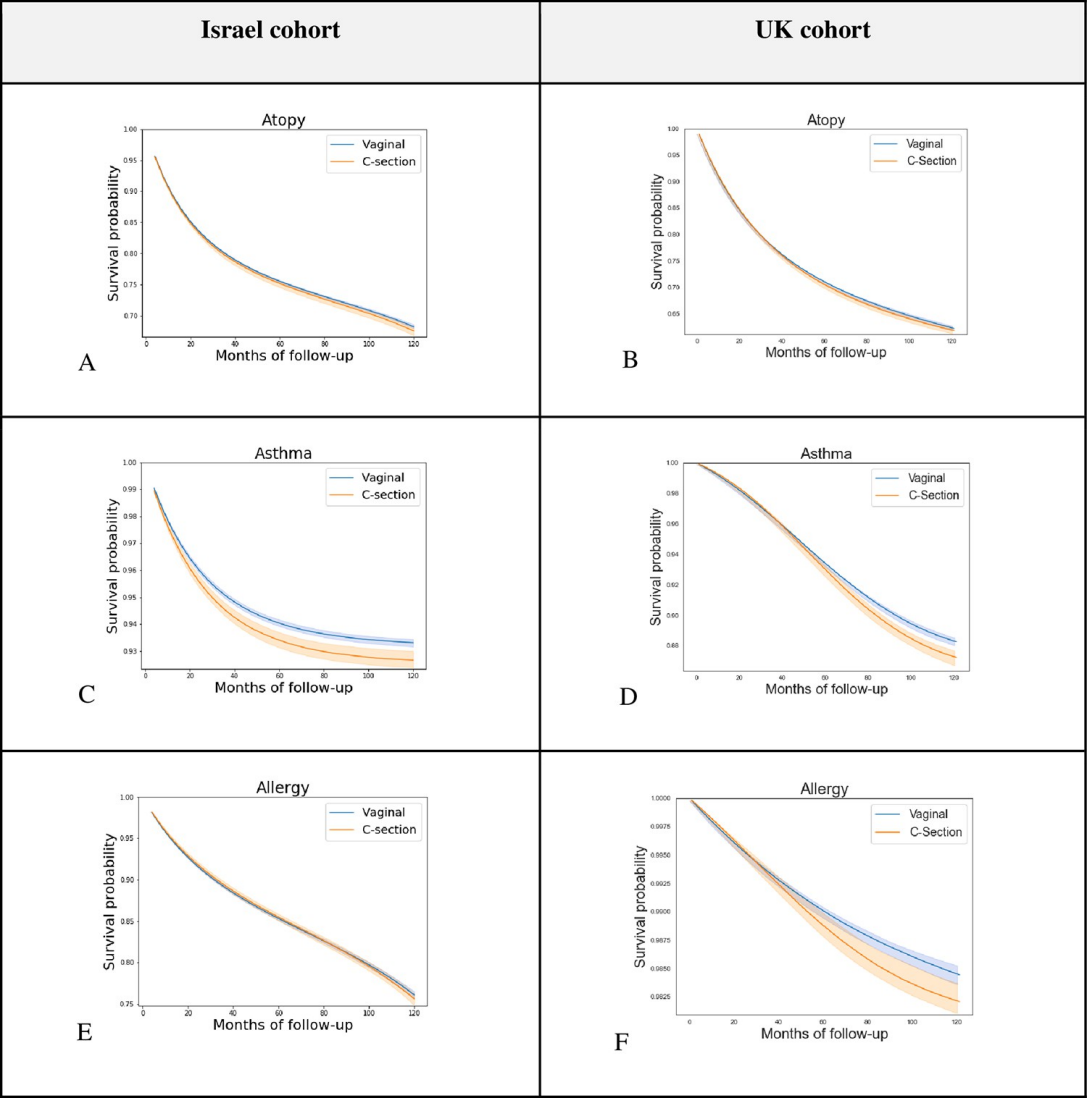
Childhood disease-free survival curves comparing vaginal and cesarean deliveries for top 3 childhood outcomes. Standardized survival curves with 95% CI of children born through vaginal delivery (blue) and CD (orange) are shown by months of follow-up. **Left:** Israel cohort, full study population. **Right:** UK cohort, full study population.

Overall, 238,159 eligible births, 84.35% vaginal and 15.65% cesarean deliveries, were included in the Israel cohort (Table 2). Fig 1 describes eligibility criteria and flow chart of cohort selection. In the replication UK cohort, 163,272 eligible births, 77.01% vaginal and 22.99% cesarean deliveries were included (Table S2.1 in S1 Appendix). Children had a mean follow-up of 5.66 (SD 2.96) years and 6.77 (SD 4.54) years, in the Israel and UK cohorts, respectively. In both cohorts, the risk for atopy, asthma and allergy was higher in children born in CD compared to vaginal delivery. For these outcomes, the 10-year standardized risk differences observed were 0.74% (-0.06, 1.52) for atopy, 0.64% (0.31, 0.98) for asthma and 0.47% (-0.32, 1.28) for allergy in the Israel cohort and 0.41% (-0.42, 1.15), 1.04% (0.49, 1.71), and 0.23% (0.07, 0.36) in the UK cohort respectively (Table 3, Figs 2 and 3). A small risk difference was also found in the Israel cohort for ADHD, 0.34% (-0.16, 0.82) and for ASD, 0.24% (0.02, 0.46), however, in the UK cohort no difference was found for ASD, and a lower 10-year risk was found for ADHD among children born through CD—but this might result from the very low incident rate of ADHD in the UK cohort. P-values for the 10-year risk survival differences were obtained for these outcomes [20] in the full cohort, and corrected for multiple hypotheses [21] for each analysis method separately (Table S6.10 in S1 Appendix). The three outcomes with higher risk in children born in CD—atopy, asthma and allergy, had significant (<0.05) p-values after correction, in at least one analysis method and one cohort (Table S6.10 in S1 Appendix). The 10-year risk difference for asthma was significant both in the Israel and the UK cohorts. An average effect of 0.10 (0.07, 0.12) and 0.92 (0.68, 1.14) was found for BMI z-score at age 5–6 years old and for the number of respiratory infection incidents until 5 years of age in the Israel cohort accordingly (Table 4). Of note, in all of these outcomes, a similar trend was observed in all estimation strategies: IPW, OW, and standardization (see Methods), with the exception of IPW on the allergy outcome in the Israeli cohort (Tables S6.1, S6.3 in S1 Appendix). Additional predefined long-term pediatric outcomes, including death, type 1 diabetes mellitus, celiac, and autoimmune diseases had a negligible risk difference both in the Israel and UK cohort. Forearm fracture, which was used as a negative control (see Methods) also had a negligible risk difference, further validating our findings. As additional stringent analyses, we next studied the effect of CD in specific subpopulations as follows:

**Table 2:** Baseline characteristics of the study population—Israeli cohort.

Characteristic, mean (SD) or counts %	Vaginal (n = 200,879) (84.3%)	Cesarean (n = 37,280) (15.7%)	All (n = 238,159)
**Neonatal characteristics**			
Sex—(Male)	102,717 (51.13%)	20,264 (54.36%)	122,981 (51.64%)
Birth weight (grams)	3,291 (391)	3,306 (460)	3,293 (403)
Gestational age at delivery (weeks)	39.32 (1.14)	38.74 (1.24)	39.23 (1.18)
**Maternal characteristics**			
Maternal age (years)	29.78 (5.34)	32.11 (5.44)	30.14 (5.42)
Maternal weight pre-pregnancy (Kg)	62.37 (12.78)	66.92 (15.51)	63.12 (13.38)
Maternal height (meters)	1.62 (0.06)	1.61 (0.07)	1.62 (0.06)
Diabetes Mellitus	2,461 (1.23%)	941 (2.52%)	3,402 (1.43%)
Chronic Hypertension	1,642 (0.82%)	814 (2.18%)	2,456 (1.03%)
Gravidity	0.77 (0.81)	0.53 (0.65)	0.73 (0.79)
Previous cesarean deliveries	6,604 (3.29%))	11,514 (30.89%)	18,118 (7.61%)
Sister’s cesarean deliveries, percent*	12.25 (27.98)	18.7 (34.38)	13.06 (28.95)
**Pregnancy characteristics**			
Gestational Diabetes	9,439 (4.7%)	3,588 (9.62%)	13,027 (5.47%)
Gestational Hypertension	1,867 (0.93%)	607 (1.63%)	2,474 (1.04%)
Gestational weight gain z-score	-0.51 (2.42)	-0.31 (2.56)	-0.47 (2.45)

Gestational weight gain z-score was calculated according to Hutcheon et al. [65].

*Sister’s CD percent was calculated as the percentage of CDs over all sister’s pregnancies which ended prior to the relevant participant’s date of birth.

**Table 3:** Estimated 10-yr childhood disease-free risk differences for comparing vaginal and cesarean deliveries.

**Full study population**						
**Childhood health outcome**	**Israel cohort**			**UK cohort**		
	**Number of incident cases**		**10-yr risk difference (95% CI)**	**Number of incident cases**		**10-yr risk difference (%)**
	**Vaginal (n = 200,879)**	**Cesarean (n = 37,280)**		**Vaginal (n = 125,743)**	**Cesarean (n = 37,529)**	
Atopy	55,505 (27.63%)	11,483 (30.8%)	0.74% (-0.06, 1.52)	35,805 (28.47%)	10,747 (28.64%)	0.41% (-0.42, 1.15)
Asthma	11,900 (5.92%)	2,756 (7.39%)	0.64% (0.31, 0.98)	9,117 (7.25%)	2,789 (7.43%)	1.04% (0.49, 1.71)
Allergy	34,045 (16.95%)	6,924 (18.57%)	0.47% (-0.32, 1.28)	1,270 (1.01%)	467 (1.24%)	0.23% (0.07, 0.36)
ADHD	6,119 (3.05%)	1,485 (3.98%)	0.34% (-0.16, 0.82)	516 (0.41%)	114 (0.3%)	-0.17% (-0.33, 0.03)
Atopic dermatitis	20,019 (9.97%)	4,201 (11.27%)	0.26% (-0.23, 0.7)	30,234 (24.04%)	9,031 (24.06%)	-0.1% (-0.87, 0.63)
Autistic spectrum disorder	1,212 (0.6%)	400 (1.07%)	0.24% (0.02, 0.46)	1,887 (1.5%)	592 (1.58%)	0.03% (-0.27, 0.39)
Death	583 (0.29%)	169 (0.45%)	0.13% (0.03, 0.24)	209 (0.17%)	73 (0.19%)	0.01% (-0.97, 0.03)
Type 1 diabetes mellitus	79 (0.04%)	20 (0.05%)	-0.01% (-0.05, 0.03)	33 (0.03%)	14 (0.04%)	0.16% (-0.01, 0.09)
Negative control (arm fracture)	3,581 (1.78%)	707 (1.9%)	-0.05% (-0.42, 0.35)	3,278 (2.61%)	951 (2.53%)	0.26% (-0.09, 0.74)
Celiac	784 (0.39%)	123 (0.33%)	-0.12% (-0.26, 0.05)	223 (0.18%)	81 (0.22%)	0.0% (-0.09, 0.09)
Autoimmune diseases	1,062 (0.53%)	178 (0.48%)	-0.13% (-0.29, 0.04)	597 (0.47%)	200 (0.53%)	0.12% (-0.04, 0.3)
**Elective CD subpopulation**						
**Childhood health outcome**	**Israel cohort**			**UK cohort**		
	**Number of incident cases**		**10-yr risk difference (95% CI)**	**Number of incident cases**		**10-yr risk difference (%)**
	**Vaginal (n = 200,879)**	**Cesarean (n = 16,379)**		**Vaginal (n = 125,743)**	**Cesarean (n = 15,479)**	
Atopy	55,505 (27.63%)	4,861 (29.68%)	-0.29% (-1.53, 0.9)	35,805 (28.47%)	4,383 (28.32%)	0.02% (-1.02, 1.77)
Asthma	11,900 (5.92%)	1,194 (7.29%)	0.37% (-0.09, 0.92)	9,117 (7.25%)	1,151 (7.44%)	0.96% (0.05, 2.33)
Allergy	34,045 (16.95%)	2,898 (17.69%)	-0.13% (-1.51, 1.28)	1,270 (1.01%)	196 (1.27%)	0.24% (0.02, 0.55)
ADHD	6,119 (3.05%)	585 (3.57%)	0.2% (-0.6, 0.92)	516 (0.41%)	53 (0.34%)	-0.05% (-0.34, 0.26)
Atopic dermatitis	20,019 (9.97%)	1,746 (10.66%)	0.33% (-0.27, 0.95)	30,234 (24.04%)	3,668 (23.7%)	-0.32% (-1.39, 1.32)
Autistic spectrum disorder	1,212 (0.6%)	178 (1.09%)	0.19% (-0.05, 0.47)	1,887 (1.5%)	249 (1.61%)	-0.09% (-0.58, 0.61)
Death	583 (0.29%)	66 (0.4%)	0.07% (-0.04, 0.22)	209 (0.17%)	23 (0.15%)	-0.03% (-0.1, 0.0)
Type 1 diabetes mellitus	79 (0.04%)	8 (0.05%)	0.0% (-0.1, 0.0)	33 (0.03%)	9 (0.06%)	0.08% (0.0, 0.17)
Negative control (arm fracture)	3,581 (1.78%)	306 (1.87%)	-0.14% (-0.72, 0.38)	3,278 (2.61%)	418 (2.7%)	0.42% (-0.25, 1.43)
Celiac	784 (0.39%)	52 (0.32%)	-0.26% (-0.42, -0.05)	223 (0.18%)	43 (0.28%)	0.8% (-0.06, 0.23)
Autoimmune diseases	1,062 (0.53%)	78 (0.48%)	-0.23% (-0.4, -0.04)	597 (0.47%)	100 (0.65%)	0.28% (0.07, 0.59)

ADHD—Attention deficit hyperactivity Disorder

**Table 4:** Estimated childhood obesity, antibiotics intake and clinic visits differences for comparing vaginal and cesarean deliveries.

Full study population						
Childhood health outcome	Israel cohort			UK cohort		
	Number of children with sufficient data		ATE difference	Number of children with sufficient data		ATE difference
	Vaginal	Cesarean		Vaginal	Cesarean	
BMI z-score at age 5–6 years old	99,345 (83.6%)	19,454 (16.4%)	0.10 (0.07, 0.12)	-	-	-
Respiratory infections—number of incidents until 5 years of age	107,654 (83.8%)	20,864 (16.2%)	0.92 (0.68, 1.14)	70,705 (78.24%)	19,666 (21.76%)	0.12 (0.08, 0.15)
**Elective CD subpopulation**						
BMI z-score at age 5–6 years old	99,345 (92.5%)	8,058 (7.5%)	0.10 (0.08, 0.12)	-	-	-
Respiratory infections—number of incidents until 5 years of age	107,654 (92.6%)	8,577 (7.4%)	1.11 (0.91, 1.35)	70,705 (89.63%)	8,181 (10.37%)	0.10 (0.06, 0.15)

BMI z-score calculated according to 2000 CDC Growth Charts for the United States [27], ATE—Average Treatment Effect

### Elective CD subpopulation

While some studies have shown a similar association between elective and unscheduled CD and pediatric outcomes [22, 23], others have shown different correlations depending on the type of CD performed [24, 25], possibly due to the fact that unscheduled CDs could result from an emergency during labor, and therefore additional factors might affect the newborn’s health. We therefore created a subgroup of children born by elective CD for both cohorts. However, while in the UK cohort, information on the type of CD was available, in the Israeli cohort it was not, and was therefore estimated by other parameters (see Methods). Overall, 217,258 children from the Israel cohort and 141,222 children from the UK cohort were included in these analyses (Tables S6.5, S6.6 in S1 Appendix). In both subgroups, standardized risk differences were still observed for asthma 0.37% (-0.09, 0.92) in the Israel cohort and 0.96% (0.05, 2.33) in the UK cohort respectively. The results for the other outcomes were not consistent in both subgroups (Table 3). In the Israeli cohort, the average treatment effect for BMI z-score at age 5–6 years old and for the number of respiratory infection incidents until 5 years of age was consistent in this subgroup with the findings in the full cohort (Table S6.3 in S1 Appendix).

### Clinics matched subpopulation

Potential confounding factors such as environment, socioeconomic status and standard of care may have a substantial impact on the validity of the results. To investigate the sensitivity of our estimates to these factors, we analyzed a subpopulation that included 66,464 children born by either CD or vaginal deliveries grouped by maternal clinics and year of birth (see Methods, Table S6.7 in S1 Appendix). In this subgroup, similarly to the full Israel cohort, a small risk difference of 0.44% (0.08, 0.88) was observed for ASD. Standardized risk differences for atopy, asthma and allergy were 0.93% (-0.36, 2.02), 0.31% (-0.14, 0.82) and 0.23% (-1.15, 1.47) respectively, and an average treatment effect of 0.09 (0.07, 0.12) and 0.96 (0.76, 1.17) was observed for BMI z-score at age 5–6 years old and for the number of respiratory infection incidents until 5 years of age. Some of these risks are smaller than those seen in the full Israel population, and they were not consistent across all analysis methods (Table S6.2, Fig S6.1 in S1 Appendix), possibly due to the relatively small sample size of this group.

### Siblings matched subpopulation

It has been previously shown that siblings studies might deal well with potential unobserved confounders related to environmental and genetic factors [26]. We analyzed a subpopulation of 3,936 children that included pairs of same-sex siblings born in discordant birth modes (see Methods, Table S6.8 in S1 Appendix). In this subgroup, we observed risk differences of 0.4% (-4.43, 5.92) for atopy, 1.7% (-0.37, 3.84) for asthma and 1.85% (-4.62, 8.32) for allergy, which also had positive 10-year risk differences across all analysis methods, apart from IPW on asthma (Table S6.2 in S1 Appendix, Fig S6.1 in S1 Appendix). Though the 95% CIs from OW on asthma include zero (Table S6.2 in S1 Appendix), and other results show larger CIs compared to the main analysis—these might stem from the very small size of this subpopulation. Overall, the same trend was observed for these outcomes in this subpopulation. Consistent with the full cohort, we observed an average treatment effect of 0.10 (0.07, 0.12) and 1.02 (0.81, 1.25) for BMI z-score at age 5–6 years old and for the number of respiratory infection incidents until 5 years of age.

### Subpopulation of women with no history of a previous CD

For women with a history of one previous CD, clinicians recommend a trial of labor after cesarean delivery (TOLAC). Yet, although TOLAC is appropriate for many women, several factors increase the likelihood of a failed trial of labor. We analyzed a subpopulation of 220,041 children whose mothers did not have a history of CDs (Table S6.9 in S1 Appendix). In this subgroup, risk differences for atopy, asthma and allergy were 0.89% (0.15, 1.8), 0.82% (0.48, 1.15) and 0.24% (-0.88, 0.94) respectively. An average effect of 0.09 (0.07, 0.12) and 0.92 (0.70, 1.16) was found for BMI z-score at age 5–6 years old and for the number of respiratory infection incidents until 5 years of age in this subpopulation. These, along with results for other outcomes, were consistent with the results observed in the full cohort (Tables S6.2, S6.4 in S1 Appendix).

Standardized survival curves for all outcomes, in the Israel and UK cohorts can be seen in Fig S6.2 in S1 Appendix. Unadjusted Kaplan-Meier curves for all outcomes and full results that include OW and IPW, as well as all sensitivity analyses can be found in Section 4 and 6 of the S1 Appendix.

## Discussion

In this study, we utilized data from the largest HMO in Israel in order to assess long-term pediatric adverse outcomes of CD, compared to vaginal delivery. While evidence regarding the effect of birth mode on future long-term outcomes of children is accumulating [1, 28], conducting an RCT to assess these effects is rarely feasible. Here, we adopted the *target trial* framework which relies on counterfactual reasoning [13] to analyse the effect of delivery mode on predefined outcomes among 238,159 children for a mean follow-up period of 5.66 (SD 2.96) years in Israel, and 163,272 children with a mean follow-up of 6.77 (SD 4.54) years in the UK. We revealed that CD had an effect on the occurrence of asthma, and may also have an effect on atopy, allergy and the number of respiratory infection events by the age of 5 years old both in the Israel and UK cohorts. An effect of CD on BMI z-scores at the age of 5 years old was also observed in the Israel cohort, but this result could not be validated in the UK cohort since routine anthropometric measurements for children were not available. Although results were not replicated for all outcomes in the different subgroups analysed, it may be a result of insufficient power to detect these differences in these small-scale cohorts, as similar trends were often observed.

The effect of mode of delivery on pediatric outcomes is hypothesized to be mediated by several mechanisms [29]. These include: hormonal surges and exposure to different levels of physical stress during labor, which may trigger protective developmental processes in the newborns and play a role in normal postnatal physiological development [30]; perturbations in the transmission of maternal microbiome to the infant during CD, which may result in different establishment and diversity of the microbiota thus possibly affecting future childhood health [31, 32]; changes in the regulation of gene expression as a result of alterations in epigenetic patterns such as DNA‐methylation [33]; abnormal short-term immune responses observed in infants born in CD, such as reduced expression of inflammatory markers [7, 34]; and exposure to general anesthesia and anesthetic medications that may cross the placental barrier [35].

Among all pediatric outcomes analysed, CD had the largest effect on atopy and asthma development (0.74% and 0.64% accordingly in the Israel cohort, 0.41% and 1.04% accordingly in the UK cohort). Risk differences for asthma were also observed in several sensitivity analysis subpopulations; 0.37% (-0.09, 0.92) in the estimated elective CD subpopulation, 0.96% (0.05, 2.33) in the UK elective CD subpopulation, 0.31% (-0.14, 0.82) in the clinics matched subpopulation and 1.7% (-0.37, 3.84) in the siblings matched subpopulation. Notably, although the magnitude of these risk differences is relatively small, the relative risk of asthma diagnosis by 10 years of age increased by almost 10% in children born by CD in the Israeli cohort and by 8.91% in the UK cohort. The association between CD and the development of asthma was previously demonstrated in numerous studies. A meta-analysis found an \ increase of 20% in the risk of asthma in children who were delivered by CD [36, 37]. A possible mechanism for this association was recently demonstrated in a study on the gut microbiome of children born by cesarean delivery showing an increased asthma risk only in children in whom microbiome composition at 1 year of age still retained a CD microbial signature, suggesting a role of altered maturation of the gut microbiota in the increased risk observed [32]. Another hypothesis underlying this association is an altered pulmonary physiology as a result of a delayed removal of amniotic fluid from the lung of infants that are born by CD, resulting in transient tachypnea of the newborn and higher incidence of respiratory distress syndrome after birth [38], which may increase the risk for asthma in the future. Interestingly, we found an average difference of 0.92 and 0.12 for the number of incidents of respiratory infections up to the age of 5 years old, in Israel and the UK respectively. This was previously demonstrated [39], and may partially mediate the effect observed on asthma, as viral infections are important causes of wheezing illnesses in children of all age ranges [40] and evidence demonstrating a link between early viral infections and asthma inception and exacerbations is accumulating [41].

The effect of CD on the incidence of atopy in children in our study is mostly driven by its effects on the development of asthma and allergy, each affected by CD when analysed separately. In the Israel cohort, the effect on atopy is larger partially due to the fact that in this cohort, atopic dermatitis is also positively affected by CD in contrast to the UK cohort (Table 3). As expected, there is an overlap between these diagnoses (as presented for the Israel cohort in Fig S4.11 in S1 Appendix). Several previous studies did not find an association between CD and atopy [5, 28] or atopic dermatitis [42, 43]. However, Meta-analyses analysing this effect display significant heterogeneity between studies [5, 28] with relatively short follow-up time in some of the studies(1–3 years) [42, 43].

A smaller risk difference was found for ADHD, 0.34% (-0.16, 0.82), and ASD, 0.24% (0.02, 0.46), between infants born by CD and those born by vaginal delivery in the Israeli cohort. These differences were smaller in the estimated elective CD subpopulation—0.2% (-0.6, 0.92) and 0.19% (-0.05, 0.47), and even smaller in the full UK cohort—-0.17% (-0.33, 0.03) and 0.03% (-0.27, 0.39), for ADHD and ASD respectively. Other subpopulations and methods used as sensitivity analyses resulted in wide estimates which included zero for both outcomes. Previous studies resulted in opposite conclusions regarding the effect of CD on the development of ADHD and ASD. While some studies found that children born by CD are roughly 20% more likely to be diagnosed with ASD [6], others concluded no association exists [44]. A previous study highlighted a possible association between general anesthesia during CD and ASD [45]. Lack of information on social parameters and variable diagnosis procedures of ADHD and ASD in different health systems might explain the variability in our results.

Finally, we have found CD had an average difference of 0.10 for BMI z-scores at age 5 years old. Very similar differences were found in all sensitivity analyses performed. The association of CD with subsequent obesity of the offspring was vastly studied, with mixed conclusions regarding the existence and magnitude of the effect. While some studies did not find any effect of delivery mode on childhood overweight [46], two meta-analyses of the literature concluded that CD is associated with an increased risk of subsequent obesity in offspring [47, 48]. It has been hypothesized that other than the above mentioned mechanisms, the difference may also arise from an altered level of appetite regulation hormones, as evident from a lower concentration of circulating ghrelin [49], and a lower umbilical leptin concentration [50] in infants born by CD.

Our study has several strengths. First, we analyzed a large and comprehensive nationwide dataset including both maternal and offspring’s data, with a long follow-up period. Second, we replicated our estimates using the same computational methods on an independent cohort which vary in both genetic and environmental factors, providing further validation to our findings. Finally, while the relation between CD and several long-term childhood outcomes has been previously investigated in many observational studies, most have concentrated on associations rather than explicitly pursuing a causal estimate.

However, our study also has several limitations. Our dataset does not contain information on some potential confounding factors such as maternal nutritional and environmental exposures. In order to minimize the effect of these confounders on our results we performed several sensitivity analyses. Another limitation is that our data did not include explicit information on whether the CD was a result of an emergency during labor versus an elective procedure, a differentiation that might be important. Although some studies found a similar association of the two CD types with pediatric outcomes [22, 23], others did not [51]. To try to overcome this we analyzed a subpopulation of CDs which were classified as elective with a high probability. In addition, we were able to analyze the subpopulation of elective CD in the UK cohort. Our data also lack information on other potential confounding factors related to labor, such as information on mode of anesthesia and usage of anesthetic medications during surgery. Finally, our results are only valid for term, appropriate for gestational age infants.

In conclusion, by emulating a *target trial* on two cohorts from different countries, we found a small causal effect of CD on pediatric asthma and childhood BMI, and a possible effect on several other pediatric health outcomes, including atopy, allergy and respiratory infections. For other outcomes, such as ASD and autoimmune diseases, an increased risk was not observed consistently when employing different methods and across both countries. Our findings might contribute to the ongoing discussion on the optimal rate of CD, with an emphasis on its adversarial effect on long-term pediatric health and may enhance discussions between clinicians and parents regarding these risks. In addition, it may pave the way to future research on the mechanism underlying these effects and possible intervention strategies targeting them.

## Methods

### Ethics declarations

The study protocol in this research was approved by the Institutional Review Board (IRB) of the Rabin Medical Center, experiment protocol number 0158-19-RMC. Informed consent was waived by the IRB, as this is a retrospective study based on unidentified data taken from EHRs. Use of IQVIA Medical Research Data (IMRD) is approved by the NHS London—South East Research Ethics Committee (REC reference: 18/LO/0441); in accordance with this approval, the study protocol was reviewed and approved by an independent Scientific Review Committee (SRC) of IQVIA Inc. (reference number: 20SRC018).

### Data

Data of the main cohort—Israel cohort, were extracted from the Clalit Health Services (Clalit) database, which is the largest Health maintenance organization (HMO) in Israel [52]. Clalit is a nongovernmental, nonprofit organization with an electronic health record (EHR) database of more than 5 million patients, representing over 50% of Israel’s adult population (Section 1a in the S1 Appendix). The data includes anthropometrics measurements, blood pressure measurements, laboratory test results, diagnoses recorded by physicians, dispensed pharmaceuticals and family linkage.

### Replication data—UK cohort

Data of the replication cohort—UK cohort, were extracted using primary care electronic health records from IQVIA Medical Research Data (IMRD), incorporating data from The Health Improvement Network (THIN, a Cegedim database). This database contains records of more than 12.5 million patients, covering approximately 6% of the UK population, and is representative of the population in terms of demographics and condition prevalence [53]. The data includes patient demographics, medical diagnoses, medication prescriptions, anthropometrics measurements and laboratory test results, which were transformed to the OMOP common data model [54].

### Study population

We analyzed a total of 737,904 births, from 2002 until 2018. CD rates in Israel were relatively stable (roughly 17%) with a small linear decreasing trend during this time period (Fig S2.1 in S1 Appendix). The cohort included offsprings of women across the whole spectrum of social deprivation in Israel (see S1 Appendix). Eligible birth records were required to contain information which we defined as critical for our analysis, including mother-child linkage and at least 5 years of documented maternal medical history in Clalit’s EHR data prior to delivery. Additional exclusion criteria were preterm birth (delivery prior to 37 completed gestational weeks) and low birth weight (birth weight below 2500 grams), as these factors can greatly impact both short-term and long-term pediatric health. Although birth weight is only measured after birth, it captures the infant’s weight prior to birth, and is not affected by the treatment—delivery mode, and can therefore be considered as an exclusion in the target trial framework. While clinicians, supported by guidelines [55], generally recommend a trial of vaginal delivery after one CD, it is generally not offered after two or more CDs. Therefore, women with a history of 2 or more CDs were also excluded. Fig 1 describes eligibility criteria and flow chart of cohort selection, and Table 2 summarizes baseline characteristics of the 238,159 eligible children. 625,044 births, from 1994 until 2019 were analyzed in the UK cohort. Of these, 250,269 births could be linked to the child’s medical record. Table S2.1 in S1 Appendix summarizes baseline characteristics of the 163,272 eligible children from this cohort.

### Statistical analysis

**Fig 4:**
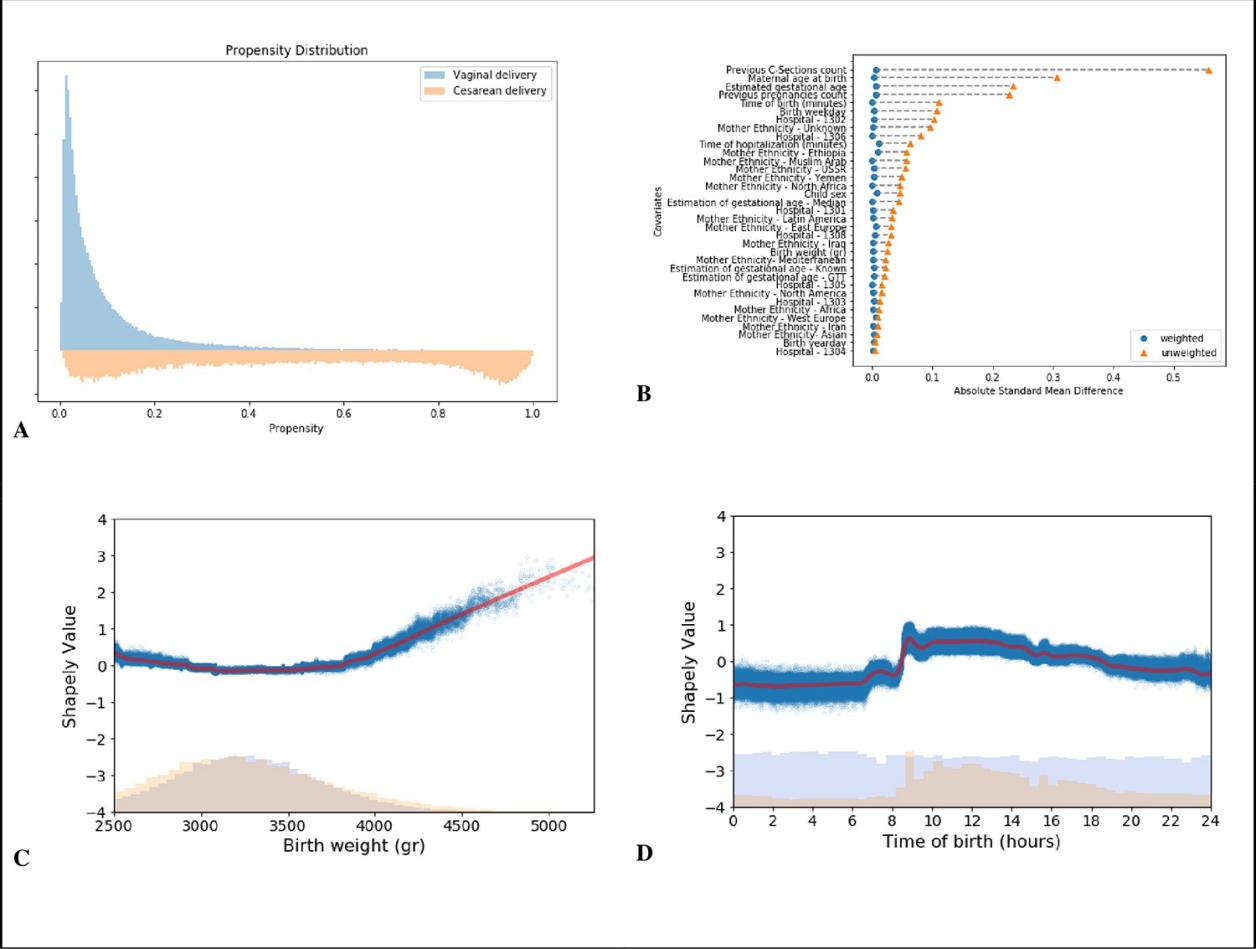
Propensity model evaluation for cesarean delivery. **A:** Distribution of propensity predictions; Blue-Vaginal delivery, Orange-CD. **B:** Absolute standardized mean differences of baseline covariates between birth mode groups. Orange triangles are the differences before weighting; Blue circles—differences after applying weighting. **C, D**: Dependence plots of birth weight and time of birth; lower part shows a histogram of the distribution of number of births, and the upper part shows a dependence plot of the log-odds Shapley value for a CD. Blue dots represent individual covariate values and Shapley values, red line represents a locally weighted scatterplot smoothing (LOWESS) regression curve.

Directed Acyclic Graphs (DAGs) [56, 57], were constructed together with physicians expert in obstetrics and pediatrics (Fig S8.1 in S1 Appendix). We trained a propensity model [58] estimating the probability of being treated, i.e. giving birth by CD, using variables selected using the DAG described above. For learning the propensity model with a large number of covariates and to allow nonlinearities, we trained Gradient Boosting trees [59]. Evaluation of the propensity model and covariate balance was done in a similar manner to the workflow described by Shimoni, Y. *et al*. [60]. In Fig 4A the distributions of propensities for each treatment group are plotted, and overlap between delivery modes is observed at least up to a score of 0.4. Covariate balance before and after reweighting is presented in Fig 4B (see Section 5 in the S1 Appendix). Applying a feature attribution framework for machine learning models based on estimated *Shapley values* [61], we were able to estimate the contribution of the baseline covariates to the estimated propensity. This setup allowed us to capture any non-linear relationships between a covariate’s contribution and the prediction value. For example, we observed a well-known [62] non-linear *U-shaped* impact of birth weight on the propensity score (Fig 4C). Another variable that had a non-linear impact on the propensity model was the time of day at delivery. It is known that elective CDs are scheduled for daytime working hours (8am-4pm), while vaginal deliveries are expected to distribute uniformly throughout the entire day (Fig S3.3 in S1 Appendix and Fig 4D). Utilizing the additive property of Shapley values, we were also able to analyze groups of related features according to domain knowledge (Fig S5.3 in S1 Appendix).

### Outcomes

Long-term childhood outcomes of CD were first identified by previous studies that demonstrated associations between these outcomes and the mode of delivery [1, 7]. Each outcome was then defined and ascertained separately by a trained pediatrician. The diagnoses were based on the relevant ICD-9 codes, laboratory test results, and prescriptions of medications for the relevant medical conditions. When feasible, the diagnoses were based on previously published diagnostic approaches from EHR available in the literature or https://phekb.org/phenotypes website, and in accordance with the Israeli healthcare policies and definitions (see section 4 in the S1 Appendix). Only outcomes with at least 10 diagnosed children who were born by each delivery mode were included in the main analysis, and the rest are presented in section 6 of the S1 Appendix. Censoring was used in case of death or if the child did not register to Clalit HMO until the age of 3 months.

### Estimation of delivery mode effect

For each of the described outcomes, we estimate the causal effect with 3 different strategies: (1) Weighting by standard (non-stabilized) inverse-probability-weighting (IPW) [18], (2) Weighting by overlap-weights (OW) [19], and (3) Standardization [17]. The different outcomes can be categorized to 2 different types: (I) time-to-event outcomes (such as asthma onset), for which survival curves were estimated; and (II) fixed continuous outcomes (such as BMI z-score), for which average treatment effect (ATE) differences were estimated. For time-to-event outcomes, survival curves were constructed by fitting a pooled logistic regression model [15, 16], on a person-time data format. Time resolution in the person-time format was 4 months, and the logistic model was fitted with a time-varying intercept and product terms between time and treatment [63]. To adjust for baseline confounders, we either added baseline selected variables to the logistic model (standardization), or performed weighting using either IPW or OW. Both weighting methods were evaluated when constructing the propensity model, OW resulted in a weighted population with better covariance balance (Fig S5.5 in S1 Appendix). 95% CIs were estimated by bootstrap sampling with 100 iterations.

### Sensitivity analysis

As some of our assumptions cannot be verified from the data, we performed several sensitivity analyses as described below.

### Negative controls

One tool to detect unmeasured confounding is the use of negative controls [64]. Here, upper forearm fracture (upper end of radius and ulna), a relatively common diagnosis in children, was chosen as negative control since no studies thus far indicated that there is a plausible association between this diagnosis and CD.

### Elective CD subpopulation

In the UK cohort, data on whether CD was unscheduled or elective was available, allowing us to create an elective CD subpopulation, which includes 141,222 children. Baseline characteristics of this subpopulation can be found in Table S6.6 in S1 Appendix. In the Israel data, a clear distinction between unscheduled and elective CD was not available. To estimate the probability of elective CD in this cohort, we utilized an additional data set, in which the type of CD was specified, from in-hospital electronic records, obtained from Rabin Medical Center, the third largest medical center in Israel. The data set contained information on 56,260 births between 2012–2020, of which 3,827 were by unscheduled CD and 3,972 by elective CD. Using this additional data, we built a model that predicts elective CD from variables that are also present in the original Clalit EHR database (see section 7 in the S1 Appendix). We used this trained model to obtain predictions for elective CD in our cohort, and created a subpopulation which contained vaginal deliveries from our full study population and CDs which were predicted as elective with a high probability (greater or equal to 0.5, selected according to the distribution of predicted probabilities) (Fig S7.2 in S1 Appendix).

### Clinics matched subpopulation

Matching by clinics can assist in minimizing confounders such as socioeconomic status that may differ between geographic locations. In this subpopulation, children were grouped by maternal clinics (defined as the most frequently visited clinic prior to pregnancy) and year of birth. In each group we sampled an equal number of CDs and vaginal deliveries.

### Siblings matched subpopulation

To create a subgroup of siblings we made use of the longitudinal and large-scale family data available in the Clalit EHRs and built a sensitivity analysis based on a subpopulation of *discordant siblings* comprised of pairs of siblings born in discordant birth modes, where both siblings are with the same sex, and matching the birth order by the number of first CD and first vaginal pairs.

### Subpopulation of women with no history of a previous CD

Using the medical history data available in Clalit, we excluded any women who had a history of birth by CD. 220,041 children, born to mothers with no CD history were included in this subpopulation.

## Supporting information

S1 Appendix (DOCX)

## References

[1] Sandall J, Tribe RM, Avery L, Mola G, Visser GH, Homer CS, et al. Short-term and long-term effects of caesarean section on the health of women and children. Lancet. 2018;392: 1349–1357. doi: 10.1016/S0140-6736(18)31930-5 30322585

[2] Boerma T, Ronsmans C, Melesse DY, Barros AJD, Barros FC, Juan L, et al. Global epidemiology of use of and disparities in caesarean sections. Lancet. 2018;392: 1341–1348. doi: 10.1016/S0140-6736(18)31928-7 30322584

[3] Barber EL, Lundsberg LS, Belanger K, Pettker CM, Funai EF, Illuzzi JL. Indications contributing to the increasing cesarean delivery rate. Obstet Gynecol. 2011;118: 29–38. doi: 10.1097/AOG.0b013e31821e5f65 21646928 PMC3751192

[4] Blustein J, Attina T, Liu M, Ryan AM, Cox LM, Blaser MJ, et al. Association of caesarean delivery with child adiposity from age 6 weeks to 15 years. Int J Obes (Lond). 2013;37: 900–906. doi: 10.1038/ijo.2013.49 23670220 PMC5007946

[5] Bager P, Wohlfahrt J, Westergaard T. Caesarean delivery and risk of atopy and allergic disease: meta-analyses. Clin Exp Allergy. 2008;38: 634–642. doi: 10.1111/j.1365-2222.2008.02939.x 18266879

[6] Curran EA, O’Neill SM, Cryan JF, Kenny LC, Dinan TG, Khashan AS, et al. Research review: Birth by caesarean section and development of autism spectrum disorder and attention-deficit/hyperactivity disorder: a systematic review and meta-analysis. J Child Psychol Psychiatry. 2015;56: 500–508. doi: 10.1111/jcpp.12351 25348074

[7] Sevelsted A, Stokholm J, Bønnelykke K, Bisgaard H. Cesarean section and chronic immune disorders. Pediatrics. 2015;135: e92–8. doi: 10.1542/peds.2014-0596 25452656

[8] Cardwell CR, Stene LC, Joner G, Cinek O, Svensson J, Goldacre MJ, et al. Caesarean section is associated with an increased risk of childhood-onset type 1 diabetes mellitus: a meta-analysis of observational studies. Diabetologia. 2008;51: 726–735. doi: 10.1007/s00125-008-0941-z 18292986

[9] Althabe F, Belizán JM. Caesarean section: the paradox. Lancet. 2006;368: 1472–1473. doi: 10.1016/S0140-6736(06)69616-5 17071266

[10] Betran AP, Torloni MR, Zhang J, Ye J, Mikolajczyk R, Deneux-Tharaux C, et al. What is the optimal rate of caesarean section at population level? A systematic review of ecologic studies. Reprod Health. 2015;12: 57. doi: 10.1186/s12978-015-0043-6 26093498 PMC4496821

[11] Wiklund I, Malata AM, Cheung NF, Cadée F. Appropriate use of caesarean section globally requires a different approach. Lancet. 2018;392: 1288–1289. doi: 10.1016/S0140-6736(18)32325-0 30322564

[12] Dickerman BA, García-Albéniz X, Logan RW, Denaxas S, Hernán MA. Avoidable flaws in observational analyses: an application to statins and cancer. Nat Med. 2019;25: 1601–1606. doi: 10.1038/s41591-019-0597-x 31591592 PMC7076561

[13] Hernán MA, Robins JM. Using big data to emulate a target trial when a randomized trial is not available. Am J Epidemiol. 2016;183: 758–764. doi: 10.1093/aje/kwv254 26994063 PMC4832051

[14] Hernán MA, Sauer BC, Hernández-Díaz S, Platt R, Shrier I. Specifying a target trial prevents immortal time bias and other self-inflicted injuries in observational analyses. J Clin Epidemiol. 2016;79: 70–75. doi: 10.1016/j.jclinepi.2016.04.014 27237061 PMC5124536

[15] Thompson WA. On the treatment of grouped observations in life studies. Biometrics. 1977;33: 463–470. 911970

[16] D’Agostino RB, Lee ML, Belanger AJ, Cupples LA, Anderson K, Kannel WB. Relation of pooled logistic regression to time dependent Cox regression analysis: the Framingham Heart Study. Stat Med. 1990;9: 1501–1515. doi: 10.1002/sim.4780091214 2281238

[17] Hernán MA, Robins JM. Causal Inference: What If. Boca Raton: Chapman & Hall/CRC; 2020. Available: https://cdn1.sph.harvard.edu/wp-content/uploads/sites/1268/2020/11/ciwhatif_hernanrobins_23nov20.pdf

[18] Rubin DB. Using propensity scores to help design observational studies: Application to the tobacco litigation. Health Serv Outcomes Res Methodol. 2001;2: 169–188. doi: 10.1023/a:1020363010465

[19] Li F, Thomas LE, Li F. Addressing extreme propensity scores via the overlap weights. Am J Epidemiol. 2019;188: 250–257. doi: 10.1093/aje/kwy201 30189042

[20] Altman DG, Bland JM. How to obtain the P value from a confidence interval. BMJ. 2011;343: d2304. doi: 10.1136/bmj.d2304 22803193

[21] Controlling the False Discovery Rate: A Practical and Powerful Approach to Multiple Testing on JSTOR. [cited 21 Jul 2022]. Available: https://www.jstor.org/stable/2346101

[22] Darmasseelane K, Hyde MJ, Santhakumaran S, Gale C, Modi N. Mode of delivery and offspring body mass index, overweight and obesity in adult life: a systematic review and meta-analysis. PLoS ONE. 2014;9: e87896. doi: 10.1371/journal.pone.0087896 24586295 PMC3935836

[23] Mueller NT, Whyatt R, Hoepner L, Oberfield S, Dominguez-Bello MG, Widen EM, et al. Prenatal exposure to antibiotics, cesarean section and risk of childhood obesity. Int J Obes (Lond). 2015;39: 665–670. doi: 10.1038/ijo.2014.180 25298276 PMC4390478

[24] Black M, Bhattacharya S, Philip S, Norman JE, McLernon DJ. Planned cesarean delivery at term and adverse outcomes in childhood health. JAMA. 2015;314: 2271–2279. doi: 10.1001/jama.2015.16176 26624826 PMC5055095

[25] Kristensen K, Henriksen L. Cesarean section and disease associated with immune function. J Allergy Clin Immunol. 2016;137: 587–590. doi: 10.1016/j.jaci.2015.07.040 26371844

[26] Donovan SJ, Susser E. Commentary: Advent of sibling designs. Int J Epidemiol. 2011;40: 345–349. doi: 10.1093/ije/dyr057 21450688 PMC3066430

[27] Kuczmarski RJ, Ogden CL, Guo SS, Grummer-Strawn LM, Flegal KM, Mei Z, et al. 2000 CDC Growth Charts for the United States: methods and development. Vital Health Stat 11. 2002; 1–190. 12043359

[28] Keag OE, Norman JE, Stock SJ. Long-term risks and benefits associated with cesarean delivery for mother, baby, and subsequent pregnancies: Systematic review and meta-analysis. PLoS Med. 2018;15: e1002494. doi: 10.1371/journal.pmed.1002494 29360829 PMC5779640

[29] Tribe RM, Taylor PD, Kelly NM, Rees D, Sandall J, Kennedy HP. Parturition and the perinatal period: can mode of delivery impact on the future health of the neonate? J Physiol (Lond). 2018;596: 5709–5722. doi: 10.1113/JP275429 29533463 PMC6265543

[30] Baines DL, Folkesson HG, Norlin A, Bingle CD, Yuan HT, Olver RE. The influence of mode of delivery, hormonal status and postnatal O2 environment on epithelial sodium channel (ENaC) expression in perinatal guinea-pig lung. J Physiol (Lond). 2000;522 Pt 1: 147–157. doi: 10.1111/j.1469-7793.2000.t01-2-00147.xm 10618159 PMC2269744

[31] Tamburini S, Shen N, Wu HC, Clemente JC. The microbiome in early life: implications for health outcomes. Nat Med. 2016;22: 713–722. doi: 10.1038/nm.4142 27387886

[32] Stokholm J, Thorsen J, Blaser MJ, Rasmussen MA, Hjelmsø M, Shah S, et al. Delivery mode and gut microbial changes correlate with an increased risk of childhood asthma. Sci Transl Med. 2020;12. doi: 10.1126/scitranslmed.aax9929 33177184

[33] Schlinzig T, Johansson S, Gunnar A, Ekström TJ, Norman M. Epigenetic modulation at birth—altered DNA-methylation in white blood cells after Caesarean section. Acta Paediatr. 2009;98: 1096–1099. doi: 10.1111/j.1651-2227.2009.01371.x 19638013

[34] Malamitsi-Puchner A, Protonotariou E, Boutsikou T, Makrakis E, Sarandakou A, Creatsas G. The influence of the mode of delivery on circulating cytokine concentrations in the perinatal period. Early Hum Dev. 2005;81: 387–392. doi: 10.1016/j.earlhumdev.2004.10.017 15814224

[35] Rizzo A, Campanile D, Spedicato M, Minoia G, Sciorsci RL. Update on anesthesia and the immune response in newborns delivered by cesarian section. Immunopharmacol Immunotoxicol. 2011;33: 581–585. doi: 10.3109/08923973.2010.549137 21275778

[36] Huang L, Chen Q, Zhao Y, Wang W, Fang F, Bao Y. Is elective cesarean section associated with a higher risk of asthma? A meta-analysis. J Asthma. 2015;52: 16–25. doi: 10.3109/02770903.2014.952435 25162303

[37] Pennington AF, Strickland MJ, Klein M, Drews-Botsch C, Hansen C, Darrow LA. Caesarean delivery, childhood asthma, and effect modification by sex: An observational study and meta-analysis. Paediatr Perinat Epidemiol. 2018;32: 495–503. doi: 10.1111/ppe.12510 30266042 PMC6261703

[38] Gerten KA, Coonrod DV, Bay RC, Chambliss LR. Cesarean delivery and respiratory distress syndrome: does labor make a difference? Am J Obstet Gynecol. 2005;193: 1061–1064. doi: 10.1016/j.ajog.2005.05.038 16157112

[39] Peters LL, Thornton C, de Jonge A, Khashan A, Tracy M, Downe S, et al. The effect of medical and operative birth interventions on child health outcomes in the first 28 days and up to 5 years of age: A linked data population-based cohort study. Birth. 2018;45: 347–357. doi: 10.1111/birt.12348 29577380 PMC6282837

[40] Gern JE. Viral respiratory infection and the link to asthma. Pediatr Infect Dis J. 2008;27: S97–103. doi: 10.1097/INF.0b013e318168b718 18820588 PMC3913380

[41] Mikhail I, Grayson MH. Asthma and viral infections: An intricate relationship. Ann Allergy Asthma Immunol. 2019;123: 352–358. doi: 10.1016/j.anai.2019.06.020 31276807 PMC7111180

[42] Laubereau B, Filipiak-Pittroff B, von Berg A, Grübl A, Reinhardt D, Wichmann HE, et al. Caesarean section and gastrointestinal symptoms, atopic dermatitis, and sensitisation during the first year of life. Arch Dis Child. 2004;89: 993–997. doi: 10.1136/adc.2003.043265 15499049 PMC1719727

[43] Papathoma E, Triga M, Fouzas S, Dimitriou G. Cesarean section delivery and development of food allergy and atopic dermatitis in early childhood. Pediatr Allergy Immunol. 2016;27: 419–424. doi: 10.1111/pai.12552 26888069

[44] Curran EA, Cryan JF, Kenny LC, Dinan TG, Kearney PM, Khashan AS. Obstetrical mode of delivery and childhood behavior and psychological development in a british cohort. J Autism Dev Disord. 2016;46: 603–614. doi: 10.1007/s10803-015-2616-1 26412364

[45] Huberman Samuel M, Meiri G, Dinstein I, Flusser H, Michaelovski A, Bashiri A, et al. Exposure to General Anesthesia May Contribute to the Association between Cesarean Delivery and Autism Spectrum Disorder. J Autism Dev Disord. 2019;49: 3127–3135. doi: 10.1007/s10803-019-04034-9 31053992

[46] Ajslev TA, Andersen CS, Gamborg M, Sørensen TIA, Jess T. Childhood overweight after establishment of the gut microbiota: the role of delivery mode, pre-pregnancy weight and early administration of antibiotics. Int J Obes (Lond). 2011;35: 522–529. doi: 10.1038/ijo.2011.27 21386800

[47] Kuhle S, Tong OS, Woolcott CG. Association between caesarean section and childhood obesity: a systematic review and meta-analysis. Obes Rev. 2015;16: 295–303. doi: 10.1111/obr.12267 25752886

[48] Li H, Zhou Y, Liu J. The impact of cesarean section on offspring overweight and obesity: a systematic review and meta-analysis. Int J Obes (Lond). 2013;37: 893–899. doi: 10.1038/ijo.2012.195 23207407

[49] Bellone S, Rapa A, Vivenza D, Vercellotti A, Petri A, Radetti G, et al. Circulating ghrelin levels in newborns are not associated to gender, body weight and hormonal parameters but depend on the type of delivery. J Endocrinol Invest. 2003;26: RC9–11. doi: 10.1007/BF03345172 12841532

[50] Yoshimitsu N, Douchi T, Kamio M, Nagata Y. Differences in umbilical venous and arterial leptin levels by mode of delivery. Obstet Gynecol. 2000;96: 342–345. doi: 10.1016/s0029-7844(00)00927-3 10960623

[51] Cai M, Loy SL, Tan KH, Godfrey KM, Gluckman PD, Chong Y-S, et al. Association of elective and emergency cesarean delivery with early childhood overweight at 12 months of age. JAMA Netw Open. 2018;1: e185025. doi: 10.1001/jamanetworkopen.2018.5025 30646378 PMC6324378

[52] We’ll be right back! [cited 8 Oct 2020]. Available: http://clalitresearch.org/about-us/our-data/

[53] Blak BT, Thompson M, Dattani H, Bourke A. Generalisability of The Health Improvement Network (THIN) database: demographics, chronic disease prevalence and mortality rates. Inform Prim Care. 2011;19: 251–255. doi: 10.14236/jhi.v19i4.820 22828580

[54] OMOP Common Data Model–OHDSI. [cited 29 Nov 2020]. Available: https://www.ohdsi.org/data-standardization/the-common-data-model/

[55] ACOG practice bulletin no. 205: vaginal birth after cesarean delivery. Obstet Gynecol. 2019;133: e110–e127. doi: 10.1097/AOG.0000000000003078 30681543

[56] Pearl J. Causality. Cambridge: Cambridge University Press; 2009. doi: 10.1017/CBO9780511803161

[57] Tennant PW, Harrison WJ, Murray EJ, Arnold KF, Berrie L, Fox MP, et al. Use of directed acyclic graphs (DAGs) in applied health research: review and recommendations. medRxiv. 2019. doi: 10.1101/2019.12.20.19015511 PMC8128477 33330936

[58] Rosenbaum PR, Rubin DB. The central role of the propensity score in observational studies for causal effects. Biometrika. 1983;70: 41–55. doi: 10.1093/biomet/70.1.41

[59] Chen T, Guestrin C. XGBoost: A Scalable Tree Boosting System. Proceedings of the 22nd ACM SIGKDD International Conference on Knowledge Discovery and Data Mining—KDD ‘16. New York, New York, USA: ACM Press; 2016. pp. 785–794. doi: 10.1145/2939672.2939785

[60] Shimoni Y, Karavani E, Ravid S, Bak P, Ng TH, Alford SH, et al. An Evaluation Toolkit to Guide Model Selection and Cohort Definition in Causal Inference. arXiv. 2019.

[61] Lundberg SM, Erion G, Chen H, DeGrave A, Prutkin JM, Nair B, et al. From Local Explanations to Global Understanding with Explainable AI for Trees. Nat Mach Intell. 2020;2: 56–67. doi: 10.1038/s42256-019-0138-9 32607472 PMC7326367

[62] Kirchengast S, Hartmann B. Recent Lifestyle Parameters Are Associated with Increasing Caesarean Section Rates among Singleton Term Births in Austria. Int J Environ Res Public Health. 2018;16. doi: 10.3390/ijerph16010014 30577604 PMC6338883

[63] Hernán MA, Robins JM. Causal Inference: What If. 2020 [cited 20 Jan 2020]. Available: https://www.hsph.harvard.edu/miguel-hernan/causal-inference-book/

[64] Lipsitch M, Tchetgen Tchetgen E, Cohen T. Negative controls: a tool for detecting confounding and bias in observational studies. Epidemiology. 2010;21: 383–388. doi: 10.1097/EDE.0b013e3181d61eeb 20335814 PMC3053408

[65] Hutcheon JA, Platt RW, Abrams B, Himes KP, Simhan HN, Bodnar LM. A weight-gain-for-gestational-age z score chart for the assessment of maternal weight gain in pregnancy. Am J Clin Nutr. 2013;97: 1062–1067. doi: 10.3945/ajcn.112.051706 23466397 PMC3625243

