# Supplementary material for: Estimating the effect of cesarean delivery on long-term childhood health across two countries
Source: PLoS One. 2022 Oct 18;17(10):e0268103. doi: 10.1371/journal.pone.0268103 (PMC9578586; doi:10.1371/journal.pone.0268103)
Supplement: S1 Appendix — (DOCX) [file pone.0268103.s001.docx]

## Supplementary Appendix

1. Data sources
2. Study cohort characteristics
3. Baseline covariates definitions
4. Pediatric outcomes definitions
5. Propensity model analysis
6. Expanded results and sensitivity analysis
7. DAGs for CD and pediatric health
8. References

### 1. Data sources

### Clalit EHRs - Israeli Cohort

Clalit is the largest of four health maintenance organizations (HMO) in Israel and one of the largest in the world. Serving over 4.4 million individuals, it includes over half of the Israeli population which is diverse in terms of race, ethnicity and socioeconomic status. Clalit owns and operates approximately 1,500 primary care clinics and 14 hospitals. Israel's adoption of EHRs started during the mid-1990’s and provision of healthcare services have been recorded in EHRs for more than 20 years. Clalit’s member population is stable (< 2% annual turnover), the data is longitudinal (spans from birth to death), includes both claims and direct clinical data and is linked through a unique anonymized identifier. The data captures administrative and clinical information across hospitals (inpatient and emergency department settings), primary care clinics, specialty clinics, pharmacies, laboratories, and clinical measures. The data are unique in their breadth, historical depth and harmonization (single EMR software), and as they originate from an operating HMO - they are constantly being generated.

#### Replication data - UK cohort

Data of the replication cohort - UK cohort, were extracted using primary care electronic health records from IQVIA Medical Research Data (IMRD), incorporating data from The Health Improvement Network (THIN, a Cegedim database). This database contains records of more than 12.5 million patients, covering approximately 6% of the UK population, and is representative of the population in terms of demographics and condition prevalence [[51]](https://sciwheel.com/work/citation?ids=9885860&pre=&suf=&sa=0). The data includes patient demographics, medical diagnoses, medication prescriptions, anthropometrics measurements and laboratory test results, which were transformed to the OMOP common data model [[52]](https://sciwheel.com/work/citation?ids=10094490&pre=&suf=&sa=0).

###

### 2. Study cohort characteristics

CD prevalence rate by year

| 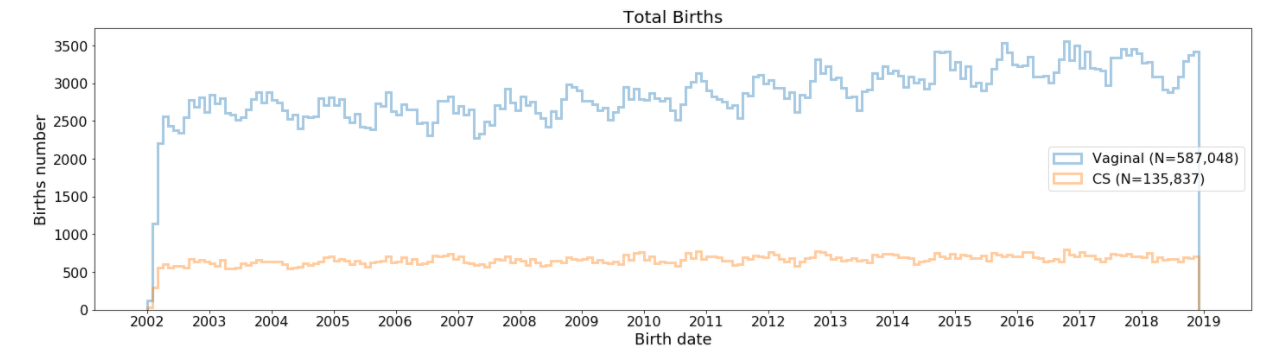 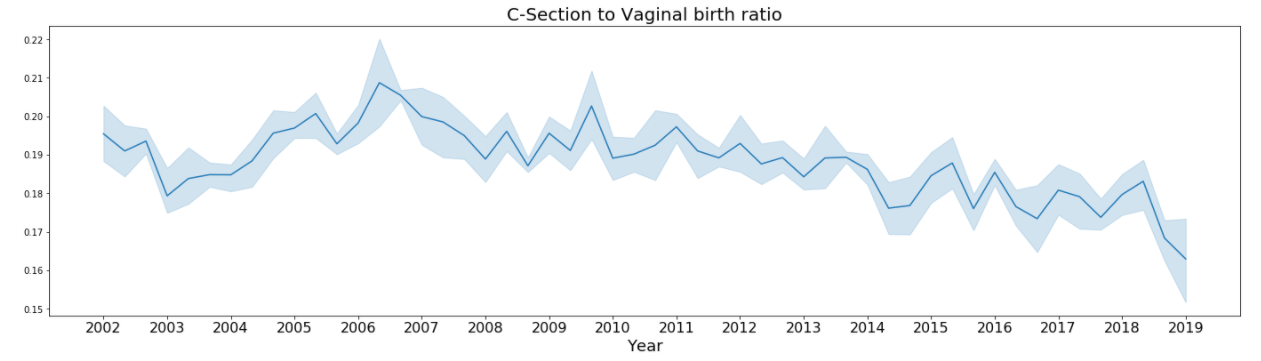 **Figure S2.1: Birth rates by years - Israel cohort. Top:** Birth rates in the years 2002 to 2019 in Clalit database; Blue-vaginal delivery, Orange-CD. **B:** CD to vaginal delivery births ratio in the years 2002 to 2019. |
| --- |

| **Table S2.1. Baseline characteristics of the study population - UK cohort** | | | |
| --- | --- | --- | --- |
| **Characteristic, mean (SD) or counts %** | **Vaginal (n = 125,743) (77.01%)** | **Cesarean (n =37,529) 22.99(%)** | **All (n = 163,272)** |
| **Infant characteristics** | | | |
| Sex - Male | 63,755 (50.70%) | 19,875 (52.96%) | 83,630 (51.20%) |
| Birth weight (grams) (N=67,692) | 3,478 (453) | 3,510 (499) | 3,485 (464) |
| Gestational age at delivery (weeks) (N=158,264) | 40.24 (1.28) | 39.78 (1.41) | 40.14 (1.32) |
| **Maternal characteristics** | | | |
| Maternal age (years) | 30.32 (5.59) | 32.07 (5.37) | 30.73 (5.56) |
| Maternal weight pre-pregnancy (Kg) (N=52,042) | 69.09 (16.36) | 72.87 (18.16) | 69.99 (16.88) |
| Maternal height (meters) (N=154,297) | 1.65 (0.07) | 1.63 (0.07) | 1.64 (0.07) |
| Diabetes Mellitus | 730 (0.58%) | 606 (1.61%) | 1336 (0.82%) |
| Chronic Hypertension | 528 (0.42%) | 287 (0.76%) | 815 (0.50%) |
| Gravidity | 0.82 (1.21) | 0.78 (1.16) | 0.81 (1.20) |
| Previous cesarean deliveries | 3206 (2.55%) | 8033 (21.40%) | 11,239 (6.88%) |
| Sister’s cesarean deliveries, percent* | - | - | - |
| **Pregnancy characteristics** | | | |
| Gestational Diabetes | 1,699 (1.35%) | 1,084 (2.89%) | 2,783 (1.70%) |
| Gestational Hypertension | 1,248 (0.99%) | 740 (1.97%) | 1988 (1.22%) |
| Gestational weight gain z-score | - | - | - |

Gestational weight gain z-score was calculated according to [Hutcheon et al. 2013](https://f1000.com/work/#/items/8196823).

*Sister’s CD percent was calculated as the percentage of CDs over all sister’s pregnancies which ended prior to the relevant participant’s date of birth.

### 3. Baseline covariates definitions

The following list describes the generation mechanism for each of the baseline covariates extracted from the data. We defined “index date” as the date of birth. All baseline covariates were extracted from data collected in the 5 years prior to the index date. Covariates were defined by a trained gynecologist and separated to three interest times; pre-pregnancy, during pregnancy and during labor\birth.

1. **Pre-pregnancy**
   1. Maternal diagnoses
      1. Diabetes diagnosis was defined by either the presence of one Diabetes code (ICD-9 codes of 250.xx), an Hemoglobin A1c test with value of at least 6.5% or a Glucose blood test with value of at least 200 mg/dL.
      2. Hypertension was defined by the presence of at least one Hypertension code (ICD-9 codes 642.2X, 642.9X)
      3. Diagnoses from previous pregnancies were defined by the presence of at least on ICD-9 code: shoulder dystocia (ICD-9 codes 660.4X) perineal tear (stage 3 or more) (ICD-9 codes 664.2X, 664.3X, 664.6X).
   2. Maternal Anthropometrics
      1. Weight and height measurements were used for calculation of mean BMI and change in BMI before and during pregnancy.
      2. Systolic and Diastolic Blood pressure mean values.
   3. Maternal Ancestry: 15 features breaking down the origin of the patient’s ancestors, as logged in their country of origin. World’s countries were clustered into 14 categories, corresponding to Israel’s major ancestry groups: North Africa, Iraq, Iran, Yemen, East Europe, West Europe, ex-USSR, North America, Latin America, Arab, Mediterranean, Ethiopia, Asia and Africa. Another feature logs the percentage of unknown origin. The distribution of the different ancestry indices in the cohort is presented in Fig. S3.1.

| 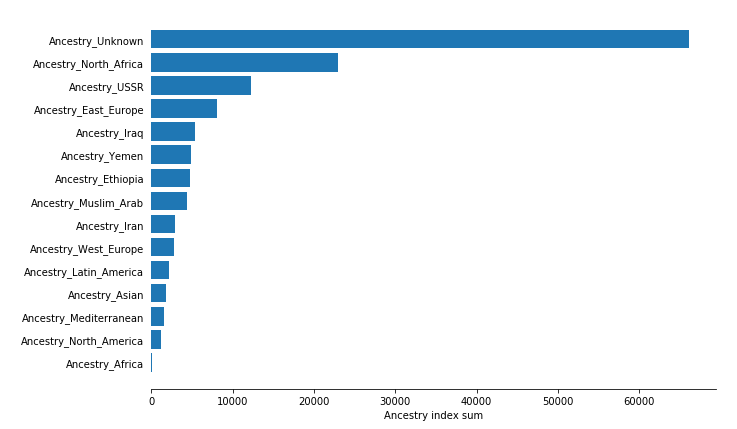  **Figure S3.1: The distribution of the different maternal ancestries index in the cohort** |
| --- |

- 1. Maternal socio-economic data: Although personal socio-economic data were not available, we generated estimates using the locality of most of the patient’s clinic visits and data available by Israel's Central Bureau of Statistics. Features include locality type (length 20, 1-hot vector) and locality religion breakdown (length 5, summing to 1 vector).
  2. Maternal and paternal age at birth.
  3. Maternal family cesarean section delivery prevalence: Utilizing the family connectivity of the data we constructed a feature which summarizes the percentage of cesarean section deliveries amongst the mother’s sisters, which can affect the risk of the mother to give birth in a cesarean section delivery [[38]](https://sciwheel.com/work/citation?ids=8137919&pre=&suf=&sa=0) Obstetrical history: Number of previous deliveries and number of previous cesarean deliveries.

#### During pregnancy

- 1. Gestational weight gain z-score was calculated using reference weight gain percentiles [[39]](https://sciwheel.com/work/citation?ids=8196823&pre=&suf=&sa=0).
  2. Maternal\fetal Diagnoses in the form of ICD9 codes during pregnancy were manually sorted by an Obstetrician. Diagnoses taken were those with medical relevance to birth type (vaginal\CD). Each diagnosis was defined by the presence of at least one ICD-9 code: Hypertensive disorders in pregnancy (ICD-9 codes 64233, 64232, 64231, 6423, 6423), major fetal anomalies (ICD-9 codes 6537, 65370, 655, 6558, 65580, 65581, 65582, 65583, 6559, 65590, 65591, 65592, 65593, 65594, 656, 65680, 65681, 65682, 65683, 65690, 65691, 65693, 6597, 65970, 65971, 65973, 678, 7429, 7439, 74400, 74403, 7443, 74600, 7462, 7469, 74720, 74740, 74760, 74761, 74762, 74763, 74764, 74782, 7479, 74860, 7489, 75010, 7509, 75160, 75240, 7529, 7539, 75550, 75560, 7559, 7561, 75610, 7579, 7589, 7599, 76381, 76383, 7780, V8903), Macrosomia (ICD-9 codes 7660, 7661, 766, 65664, 65663, 65662, 65661, 65660, 6566), malpresentation\malposition (ICD-9 codes 652.XX, 660.X), placental abruption (ICD-9 codes 641.X), Oligohydramnios (ICD-9 codes 658.0X) and Polyhydramnios (ICD-9 codes 657.X), Preeclampsia (ICD-9 codes
  3. Gestational diabetes (GDM) was defined by the results of a glucose challenge test. Either a Glucose challenge test of 50g with a measurement above 200 mg/dL or a Glucose challenge test of 100g with one glucose measurements above the thresholds of 95, 180, 155 and 140 mg/dL under fasting conditions - fasting, one, two and three hours after glucose intake, respectively, were considered as a positive GDM diagnosis.

#### Labor\birth

- 1. Diagnoses which occur during labor which are medically relevant to birth type (vaginal\CD) were selected by a gynecologist, and defined by the presence of at least one ICD-9 code in the hospital’s records: Non reassuring fetal heart rate (NRFHR) (ICD-9 codes 656.3X, 659.7X), arrest of descent\arrest of dilatation (ICD-9 codes 652.5X, 660.9X, 660.8X, 660.6X), failed induction (ICD-9 codes 659.X) and failed vacuum\forceps (ICD-9 codes 660.7X).
  2. Gestational age: For children born in a Clalit hospital after 2012, gestational age information was available in the data. For women with available GCT results, performed routinely in Israel at 24-28 weeks of gestation, GCT result was used as a proxy for week 26 of gestation and then used for approximation of gestational age. Otherwise median gestational age in the Clalit birth data was taken - 39 weeks.
  3. Hospital code.
  4. Newborn birth weight.
  5. Newborn sex.
  6. Birth weekday and year day (Fig. S3.2).
  7. Birth hour of day (Fig. S3.3).
  8. Admission hour of the day (Fig. S3.3).

#

| 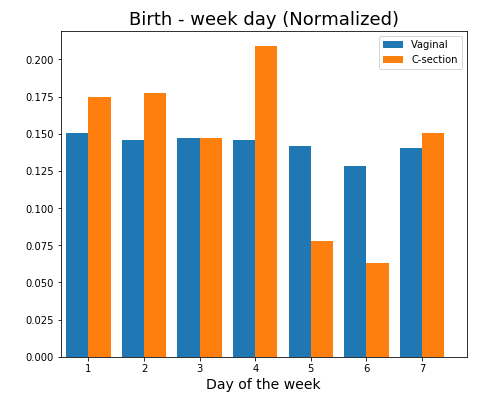 | 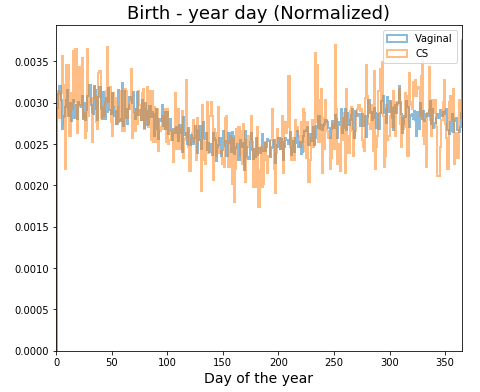 |
| --- | --- |
| **Figure S3.2: Left:** Normalized histogram of birth week day. **Right:** Normalized distribution of birth year day. | |

| **A B**  **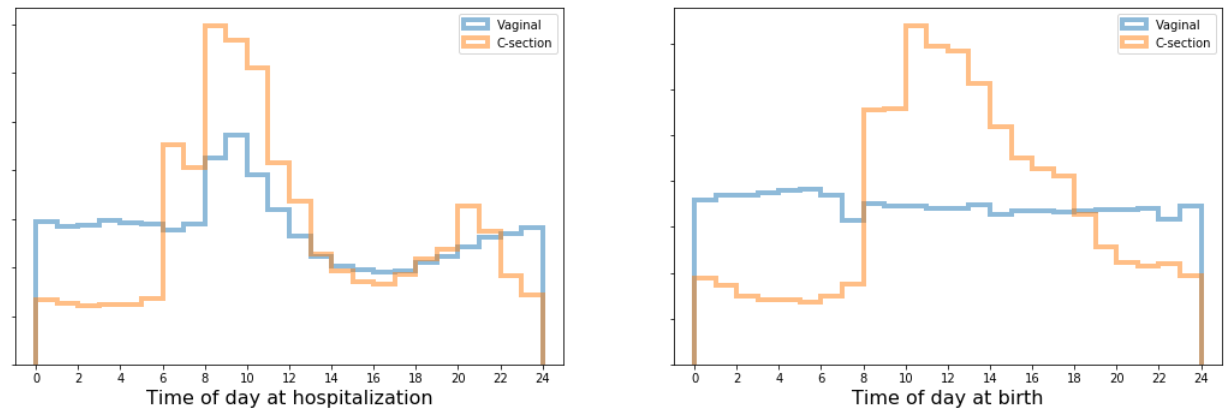**  **Figure S3.3:** Normalized distribution of birth related time features**. A.** Time of admission to the hospital. **B.** Time of birth. |
| --- |

### 4. Pediatric outcomes definitions

First, potential long-term childhood outcomes of cesarean section delivery were identified by previous studies that demonstrated correlations between these outcomes and delivery by cesarean section [[1,7]](https://sciwheel.com/work/citation?ids=5886892,2129196&pre=&pre=&suf=&suf=&sa=0,0). Second, each outcome was defined separately by a trained pediatrician, as defined below.

Notably, the same definitions were also used in the UK cohort analysis. Diagnosis codes were converted from ICD9 and ICD10 to standardized SNOMED-CT codes. For outcome definitions requiring medication regimens, specific medications were added according to their generic and/or commercial names as used in the UK.

#### Asthma

It has been previously demonstrated that identifying asthma cases in electronic health records is possible with high sensitivity and specificity, by combining multiple data sources [[40]](about:blank). We defined asthma status based on the definition in the PheKB website <https://phekb.org/phenotype/asthma-response-inhaled-steroids>. As this definition refers specifically to patients with asthma who use inhaled steroids, we expanded the definition to all asthma drugs. More specifically, a diagnosis of asthma was considered if all of the following criteria were met:

1. At least 2 asthma codes on different days were present (ICD-9 codes of 493.xx)
2. At least 1 asthma drug was dispensed (Salbutamol (R03AC02), Terbutaline (R03AC03), Salmeterol (R03AC12), Seretide Cd (R03AK06), Symbicort/Duoresp (R03AK07), , Beclometasone (R03BA01), Budesonide (R03BA02), Fluticasone (R03BA05) , Ipratropium Bromide (R03BB01), Salbutamol (R03CC02), Terbutaline (R03CC03), Montelukast (R03DC03), Budesonide (R01AD05), Fluticasone (R01AD08).
3. Children with the following diagnoses were censored for the asthma outcome: Cystic Fibrosis 277.00 - 277.02, Immunodeficiency 279.xx, Bronchiectasis 494.0 - 494.1, Hereditary and degenerative diseases of CNS (331), Mental Retardation (317,318,319), Congestive Heart Failure 428-429.9, Pulmonary Hypertension, Embolism 415.11-415.19, 416.0 - 416.8, 417.XX, COPD 496, lupus (710.0), RA (714.0), Tuberculosis (011.1), Lung cancer (162.*), Sarcoidosis (135)

| **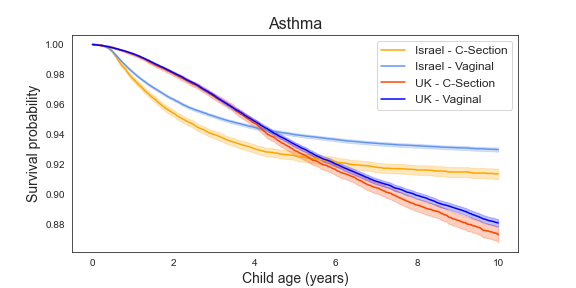**  **Figure S4.1:** Kaplan-Meier unadjusted curves for asthma outcome in children born in vaginal delivery or CD, in Israel (orange and light blue accordingly) and UK cohorts (red and dark blue accordingly). |
| --- |

#### Overweight\Obesity

We defined obesity status in accordance with health care professionals in Israel, using the CDC BMI reference percentiles [[25]](https://sciwheel.com/work/citation?ids=1111621&pre=&suf=&sa=0). Cutoffs for normal weight, overweight, and obesity were determined using the CDC's standard thresholds of the 85th percentile for overweight and 95th percentile for obesity. Of note, it was previously shown similar estimates of obesity risk at 5 years of age as other percentile curves such as The World Health Organization (WHO) WFL [[41]](https://sciwheel.com/work/citation?ids=6652734&pre=&suf=&sa=0). Valid measurements were defined as being in the range of 5 CDC standard deviation scores for weight and height.

#### Atopic dermatitis

Was defined as the presence of at least 2 atopic dermatitis codes on different days (ICD-9 codes 6918, S87, 691).

| **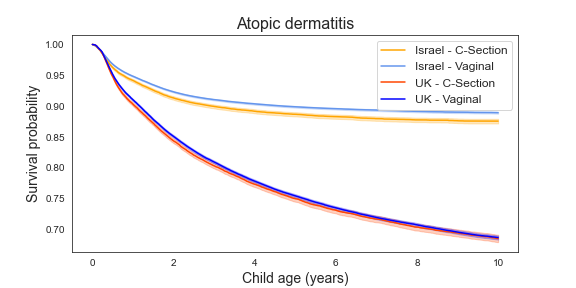**  **Figure S4.2:** Kaplan-Meier unadjusted curves for atopic dermatitis outcome in children born in vaginal delivery or CD, in Israel (orange and light blue accordingly) and UK cohorts (red and dark blue accordingly). |
| --- |

#### Celiac disease

Was defined as the presence of at least 2 Celiac codes on different days (ICD-9 code 5790).

| **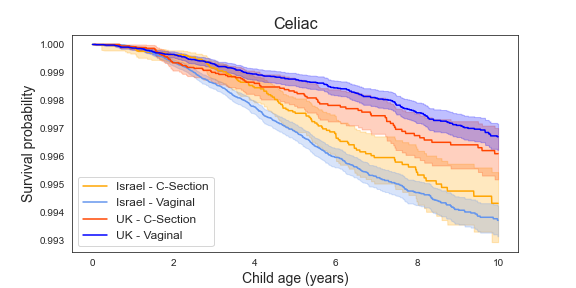**  **Figure S4.3:** Kaplan-Meier unadjusted curves for celiac outcome in children born in vaginal delivery or CD, in Israel (orange and light blue accordingly) and UK cohorts (red and dark blue accordingly). |
| --- |

#### Allergy

The task of clinical coding in allergy is complex, as allergic disorders have a wide range, they occur across the entire life course, and there are several organ systems that can be affected [[42]](https://sciwheel.com/work/citation?ids=7783245&pre=&suf=&sa=0). Allergy was defined by the presence of one of the following criteria:

1. At least one ICD-9 diagnosis (expanded definition based on Hirsch et al. [[43]](https://sciwheel.com/work/citation?ids=3889195&pre=&suf=&sa=0)), including: Food allergy cases : milk allergy ICD‐9 code (V1502, 99567) and Non‐milk food allergy cases (e.g. nuts, seafood) ICD‐9 code (V1501, V1503, V1504, V1505, 6931, 99560, 99561, 99563, 99564, 99565, 99566, 99568, 99569, 9957), drug allergy ICD‐9 code (V140, V141, V142,V143, V144, v145, V146, V147, V148, V149, 99527, V1508), other allergies (e.g. Latex, Insects), allergic rhinitis, allergic purpura,(allergic urticaria, allergic conjunctivitis, allergic pneumonitis, allergic arthritis, allergic gastroenteritis ICD‐9 code (9953, 4770, 4772, 4778, 4779, A12, 7080, R97, 2870, 5583, 7162, 38104, 37214, 4959, 4958, 38105, 71626, Z9912, V1506, v1507, v150 , V1509, V1381.
2. At least one prescription of the drug Epipen (ATC codes:C01CA24)

| **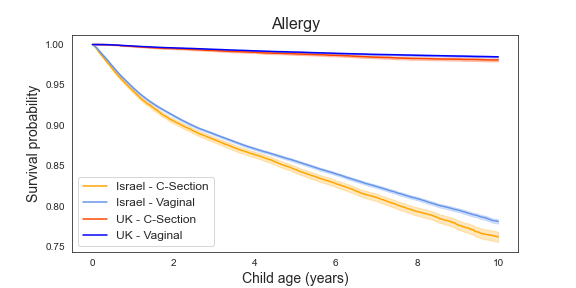**  **Figure S4.4:** Kaplan-Meier unadjusted curves for allergy outcome in children born in Vaginal delivery or CD, in Israel (orange and light blue accordingly) and UK cohorts (red and dark blue accordingly). |
| --- |

#### Autistic spectrum disorder (ASD)

Was defined based on a previous study [[44]](https://sciwheel.com/work/citation?ids=2989354&pre=&suf=&sa=0). As information from clinical notes was not available as part of our data, ASD was defined as follows: at least 1 ICD-9 codes for ASD, Asperger’s or Pervasive Developmental Disorder-Not Otherwise Specified (PDD-NOS), ICD-9 codes (299.0, 299.80,299.9, F84.0, F84.1, F84, F84.9, F84.5, F84.8)

Children with the following diagnoses were censored for the ASD outcome: Childhood disintegrative disorder (ICD-9 code 299.1), Schizophrenia (ICD-9 codes 295.X), Tuberous Sclerosis (ICD-9 code 759.5), Fragile X Syndrome (ICD-9 code 759.83), Rett’s syndrome (F84.2) and Other specified cerebral degenerations in childhood (ICD-9 code 330.8).

| **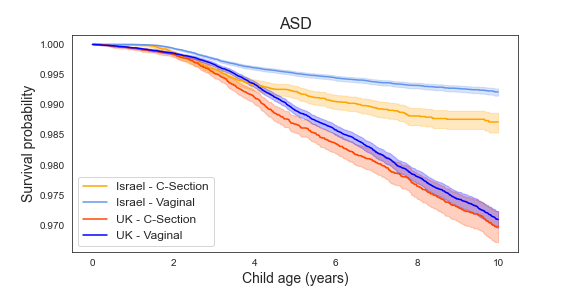**  **Figure S4.5:** Kaplan-Meier unadjusted curves for ASD outcome in children born in vaginal delivery or CD, in Israel (orange and light blue accordingly) and UK cohorts (red and dark blue accordingly). |
| --- |

#### ADHD

We defined ADHD based on the definition in the PheKB website, based on the following study [[45]](https://sciwheel.com/work/citation?ids=4519071&pre=&suf=&sa=0). ADHD cases must be 4 years of age or older, and are defined by a diagnostic history of ADHD as determined by the relevant ICD9 codes and/or a history of ADHD medications, based on the following criteria:

1. Age equals or greater than 4 years old

and

1. At least 1 relevant ICD-9 diagnosis codes on different days was present (ICD-9 code 314.0, 314.01, 314.00, 7745, 314.2, F908, F90, F909, 314.9)

and

1. Individual’s medical record includes one or more prescriptions of ADHD-related medications (as previously classified [[46]](https://sciwheel.com/work/citation?ids=3447956&pre=&suf=&sa=0): methylphenidate (ATC code N06BA04), atomoxetine (N06BA09), amphetamine (N06BA01) and dexamphetamine (N06BA02)

Or:

1. Age equals or greater than 4 years

and

1. At least two relevant ICD-9 diagnosis codes on different days were present ICD-9 code (314.0, 314.01, 314.00, 7745, 314.2, F908, F90, F909, 314.9)

Children with the following diagnoses were censored for the ADHD outcome: : Dementia (2900, 2904, F03, F01), Somatoform disorder (F45, 30081, 30089, 3008, F453), Stereotyped Repetitive Movements (3073), Mental Retardation (317, F70, F71, 3182, F711, 3180, F721, F720, F701, F700, F708, F710, F728, F718, F73, F730, F731, F790), CNS Tuberculoma (132), CNS Malignancies ( 1983, 1917, 1910, 1911, 1912, 1913, 1914, 1915, 1916, 1917, 1918, 1919, 1920, 1922, 1928), Neurofibromatosis (23770, 23772, 2377,23771),Anoxic Brain Damage (3481) , Benign Intracranial Hypertension (3482), Encephalopathy, Unspecified (3483), Compression Of Brain (3484), Cerebral Edema (3485), Temporal Sclerosis (34881), Brain Death (34882), Cerebral Calcification (348891), Other Conditions Of Brain (34889), Hypertensive Encephalopathy (4372), Other congenital anomalies of nervous system (742-742.x ), Slow fetal growth and fetal malnutrition (764-764.x), Subdural and cerebral hemorrhage (767.0), Birth trauma, unspecified (767.9 ), Fracture of skull (800-804), Head injury, unspecified (959.01).

| **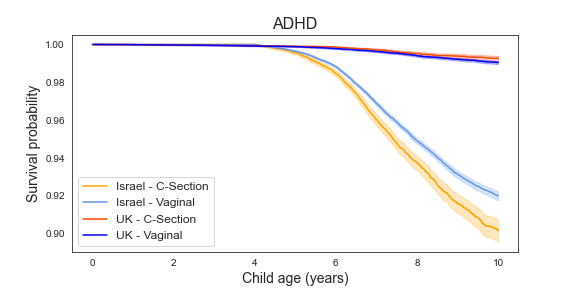**  **Figure S4.6:** Kaplan-Meier unadjusted curves for ADHD outcome in children born in vaginal delivery or CD, in Israel (orange and light blue accordingly) and UK cohorts (red and dark blue accordingly). |
| --- |

#### Inflammatory bowel disease

Was defined as the presence of at least two relevant ICD-9 diagnosis on different days. Ulcerative colitis (UC) ICD-9 codes 556.X and crohn's disease (CD): ICD9 - 555.X

| **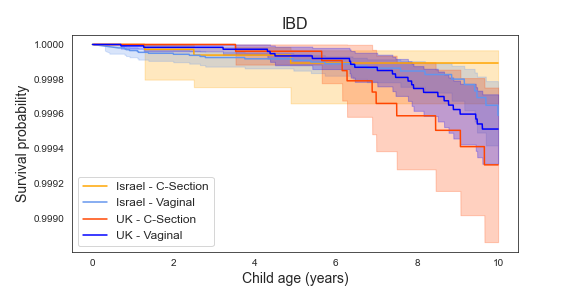**  **Figure S4.7:** Kaplan-Meier unadjusted curves for IBD outcome in children born in vaginal delivery or CD, in Israel (orange and light blue accordingly) and UK cohorts (red and dark blue accordingly). |
| --- |

#### Type 1 diabetes

We defined Type 1 diabetes based on the definition in the PheKB website, based on the following

Study [[47]](https://sciwheel.com/work/citation?ids=7729773&pre=&suf=&sa=0), with an adaptation, as it was originally used for diagnosing both type 1 & 2 diabetes. We defined that a patient has T1DM based on the following criteria:

1. One or more [outpatient](https://www.sciencedirect.com/topics/medicine-and-dentistry/outpatient) diabetes-related ICD-9- diagnosis codes (250, 2500, 2501, 25001, 25011, 25013,25003, 2504, 25061, 25081, 2508, 25083, 25091, 25093) as well as a local Clalit diagnosis code “Diabetes Routine Follow Up” (8).

Or

1. At least two prescriptions of Insulin (ATC code A10AB0X)

And

Hemoglobin A1c (HbA1C) > 6.4 in at least one blood test

Children with the following diagnoses were censored for the T1DM outcome:Adult-onset Type Diabetes Mellitus, Neonatal Diabetes Mellitus, Mody Mature Onset Diabetes In Young And Secondary Diabetes Mellitus (ICD-9 codes 25000, 25080, 25010, 7751, 25050, E118, 24901, 25020, 24900, 25012, 25002).

| **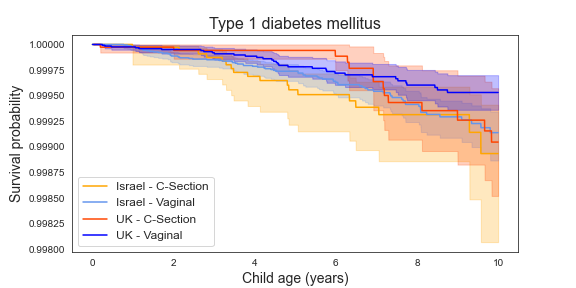**  **Figure S4.8:** Kaplan-Meier unadjusted curves for type 1 diabetes outcome in children born in vaginal delivery or CD, in Israel (orange and light blue accordingly) and UK cohorts (red and dark blue accordingly). |
| --- |

#### Juvenile idiopathic arthritis

Was defined as the presence of at least 2 relevant ICD-9 codes on different days ( 7143 ,71430, 71432, 71433, and 71431).

| **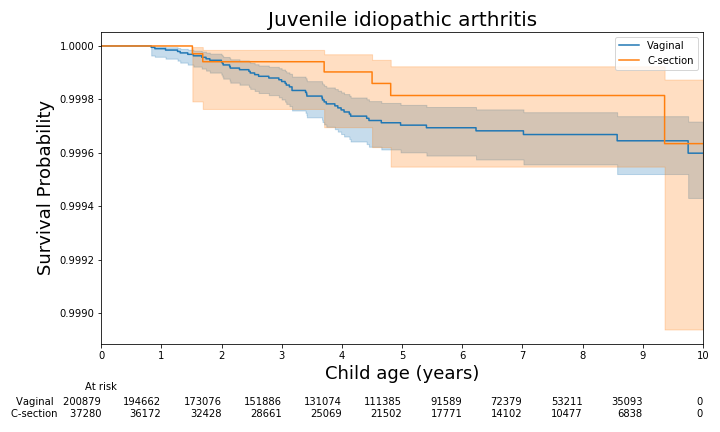**  **Figure S4.9:** Kaplan-Meier unadjusted curves for type 1 diabetes outcome in children born in vaginal delivery (blue) or CD (orange) in Israel cohort. |
| --- |

#### Respiratory infections

Acute respiratory infection (ARI) was defined as the number of the following ICD-9 diagnosis codes until the age of 5 years [[48]](https://sciwheel.com/work/citation?ids=7783755&pre=&suf=&sa=0): nonspecific URIs (ICD-9-CM 460.X, 464.X, and 465.X), otitis media (ICD-9-CM 381.X (with the exception of 381.6 and 381.7) and 382.X , sinusitis (ICD-9-CM 461.X and 473.X), pharyngitis (ICD-9-CM 034.0 ,462, and 463), acute bronchitis (ICD-9-CM 466 and 490), pneumonia (ICD-9-CM 481–486), and influenza (ICD-9-CM 487) in addition to local Clalit diagnostic codes (R81 pneumonia, H72 serous otitis media , R74 URI, H71 Acute OM

Children with the following ICD-9 codes were censored from the Respiratory infections outcome: Cystic Fibrosis 277.00 - 277.02, Immunodeficiency 279.xx, Bronchiectasis 494.0 - 494.1, Hereditary and degenerative diseases of CNS (331), Mental Retardation (317,318,319), Congestive Heart Failure 428-429.9, Pulmonary Hypertension, Embolism 415.11-415.19, 416.0 - 416.8, 417.XX, COPD 496, lupus (710.0), RA (714.0), Tuberculosis (511.1), Lung cancer (162.*), Sarcoidosis (135)

#### Autoimmune disease

Many autoimmune diseases are very rare in the pediatric population. To identify autoimmune diseases in our data, we first extract an initial list of autoimmune diseases as defined by ‘Autoimmune Disease’ (D001327) in the MeSH® hierarchy <https://meshb.nlm.nih.gov/record/ui?ui=D001327> , i.e., all the descriptors with a MeSH® Tree Number starting with C20.111.

A patient was considered as having Autoimmune diseases in the presence of at least 2 relevant ICD-9 codes on different days including: Addison Disease [C20.111.163] - ICD-9 code 25541, Anemia, Hemolytic, Autoimmune [C20.111.175] - ICD-9 code 2830, [Graves Disease [C20.111.555]](https://meshb.nlm.nih.gov/record/ui?ui=D006111) - ICD-9 codes 2420, 24200, Hepatitis, Autoimmune [C20.111.567] - ICD-9 code 57142, Lupus Erythematosus, Systemic [C20.111.590] - ICD-9 codes 7100, 6954, Pemphigus [C20.111.736] - ICD-9 code 6944, Purpura, Thrombocytopenic, Idiopathic [C20.111.759] - ICD-9 code 28731, Thyroiditis, Autoimmune [C20.111.809] - ICD-9 code 2452, Undifferentiated Connective Tissue Diseases [C20.111.904] - ICD-9 code 7109, Autoimmune Diseases of the Nervous System [C20.111.258] included Multiple sclerosis - ICD-9 codes: 340, 3400, Acute inflammatory demyelinating polyneuropathy (Guillain-Barré syndrome) - ICD-9 code 3570 and Myasthenia Gravis - ICD-9 codes 3580 and 35800.

The following diseases were diagnosed as described above: Arthritis, Juvenile [C20.111.198] - section J , Diabetes Mellitus, Type 1 [C20.111.327] - Section I, Celiac disease - Section D.

| **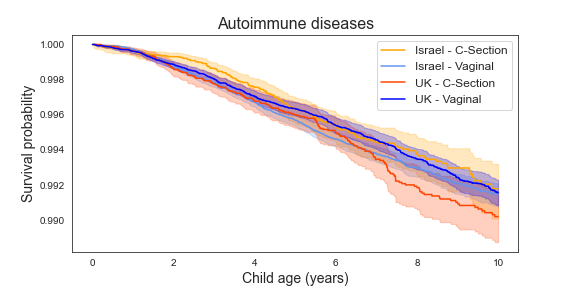**  **Figure S4.10:** Kaplan-Meier unadjusted curves for autoimmune diseases outcome in children born in vaginal delivery or CD, in Israel (orange and light blue accordingly) and UK cohorts (red and dark blue accordingly). |
| --- |

#### Atopy

Was defined as children who fulfilled the criteria described above for one of the following phenotypes: asthma (Section A), allergy (Section E) and atopic dermatitis (Section C).

| 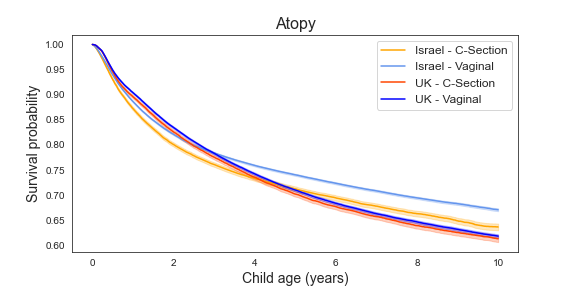 | 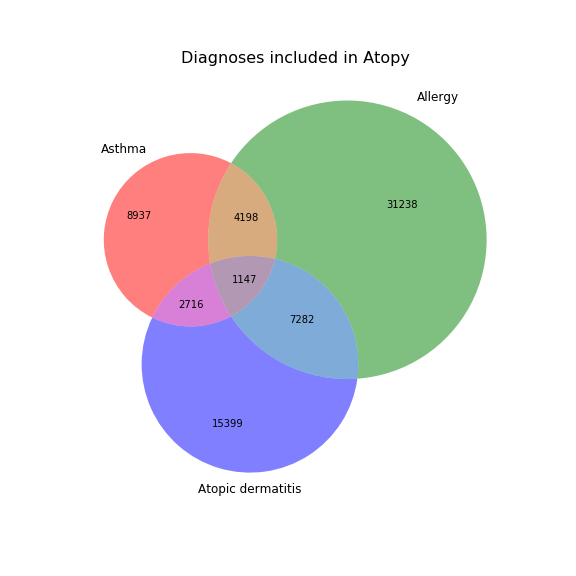 |
| --- | --- |
| **Figure S4.11: Left:** Kaplan-Meier unadjusted curves for Atopy outcome in children born in vaginal delivery or CD, in Israel (orange and light blue accordingly) and UK cohorts (red and dark blue accordingly). **Right:** Venn diagram of the outcomes included in the atopy outcome, from Israel cohort. | |

#### Negative Controls

The following pediatric outcomes were defined as negative controls, as they are not expected to be affected by c/s delivery compared to a normal vaginal delivery according to existing medical literature.

1. Fracture of upper end of radius and ulna, closed - ICD-9 code 8130.

| 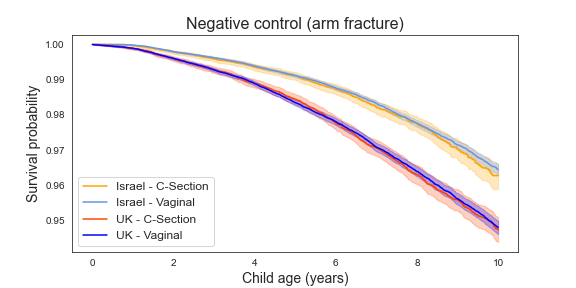  **Figure S4.12:** Kaplan-Meier unadjusted curves for arm fracture (negative control) outcome in children born in vaginal delivery or CD, in Israel (orange and light blue accordingly) and UK cohorts (red and dark blue accordingly). |
| --- |

### 5. Propensity model analysis

#### Prediction model parameters

We used a gradient boosting trees model trained with the XGBOOST python package. Hyperparameters were selected following a cross-validated grid search, with the following settings selected:

- nuestimators= 500
- max_depth= 3
- colsample_bytree = 0.8
- subsample = 0.8

Missing values were not imputed prior to training the model. XGBOOST uses the method of *block propagation* in which tree splits are learned only from non-missing data, and only after that, the direction of splitting missing values is learned (by minimizing error) for the whole block of samples for missing values for that feature. Josse et al has shown that this procedure is a good option relative to various imputation methods [[49]](https://sciwheel.com/work/citation?ids=7217224&pre=&suf=&sa=0).

| 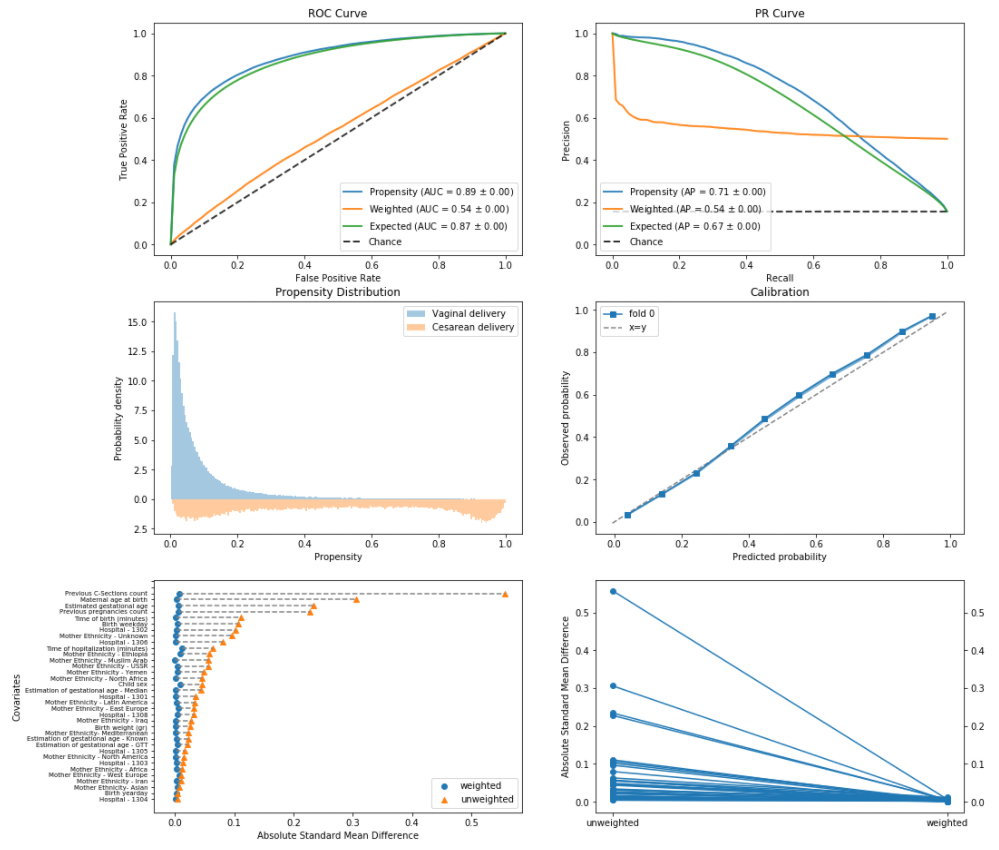  **Figure S5.1:** Propensity model evaluation on train set. |
| --- |

| 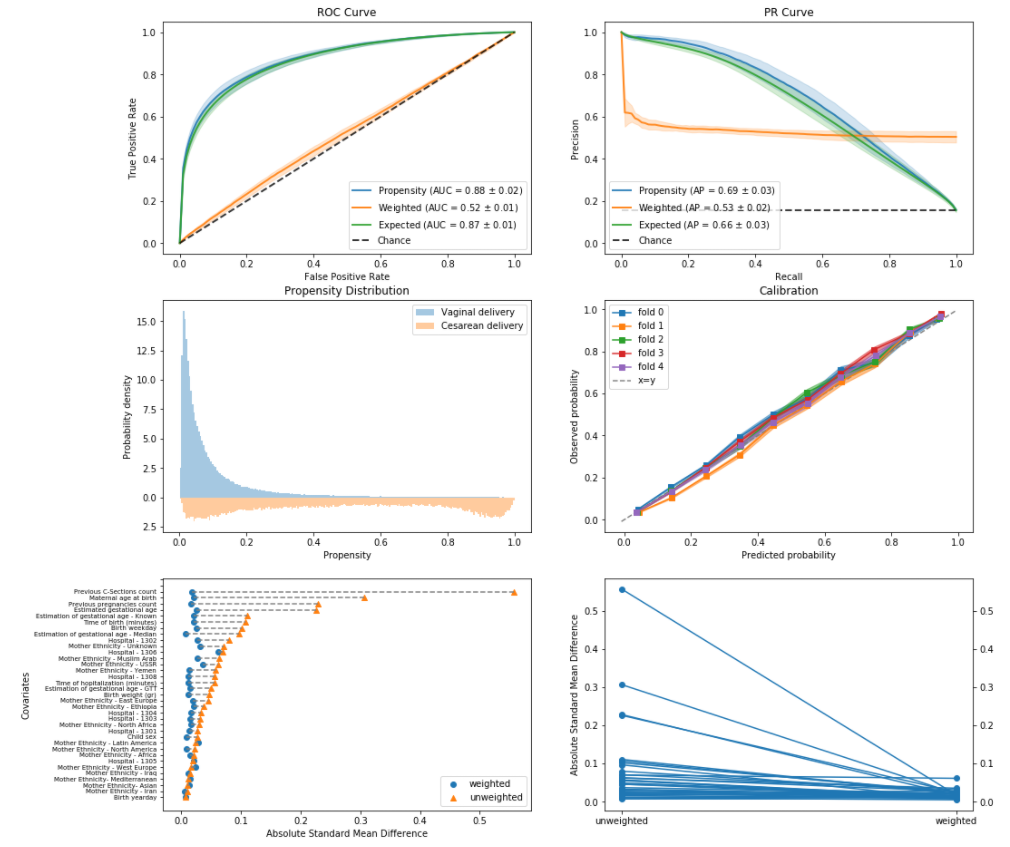  **Figure S5.2:** Propensity model evaluation on cross-validated test sets |
| --- |

####

#### Extended feature attribution analysis

| 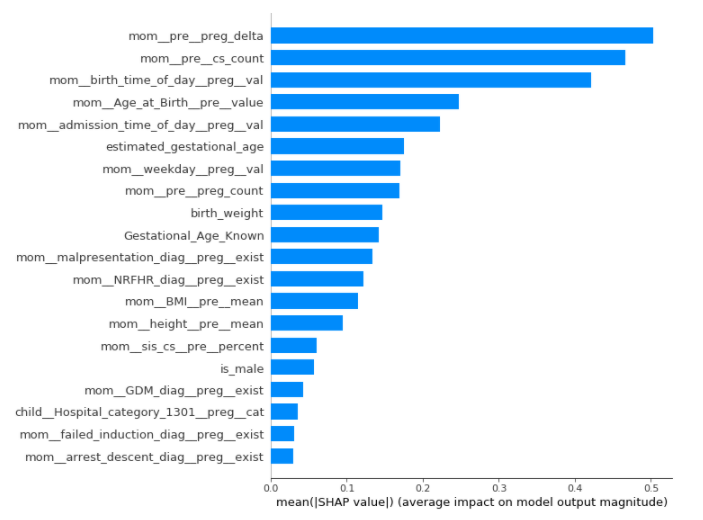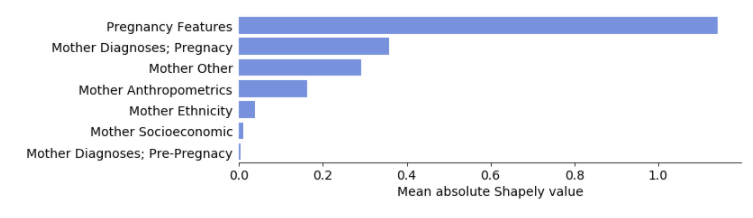  **Figure S5.3: A:** Mean absolute Shapley values (in log-odds scale) of the propensity prediction model. 20 top contributing features are shown. **B:** Mean absolute Shapley values (in log-odds scale) of groups of features. “Others” sums up remaining features not included in the rest of the groups such as blood pressure measurements and father’s age. |
| --- |

###

| **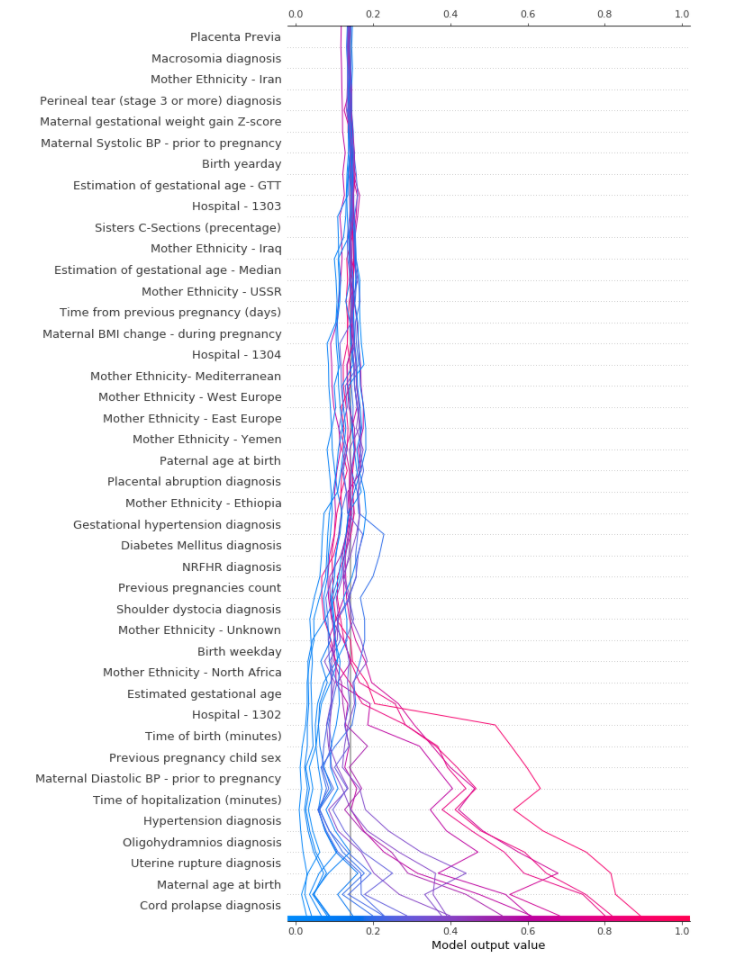**  **Figure S5.4: Decision plot of the probability of CD.** A random sample of the population is presented. Each individual’s prediction is represented by a colored line. At the bottom, each line strikes the x-axis at its corresponding predicted probability. This value determines the color of the line. Moving from top to bottom, SHAP values for each feature are added to the model’s base value. |
| --- |

####

#### Weighting methods

Weighting methods aim to adjust for baseline confounders by creating a new weighted pseudo-population for which all variable distributions are identical in the treatment and control groups. These methods use the estimated propensity score, $e_{i}$, to weigh each individual in the cohort. When using IPW the weight for each individual is defined as $w_{i}=1/e_{i}$for treated units and $w_{i}=1/(1-e_{i})$ for control units. When the treated and control populations differ, some individuals might have extreme propensity scores, which in turn leads to very high inverse-propensity weights. When using OW the weight for each individual is defined as $w_{i}=1-e_{i}$for a treated unit and $w_{i}=e_{i}$ for a control unit [[19]](https://sciwheel.com/work/citation?ids=8324953&pre=&suf=&sa=0). The construction of these weights allows up-weighing of individuals that were equally likely to be treated\untreated, and smoothly down-weighing individuals in the tails of the propensity score distribution. This allows for a weighted population that more closely emulates a target trial population.

| 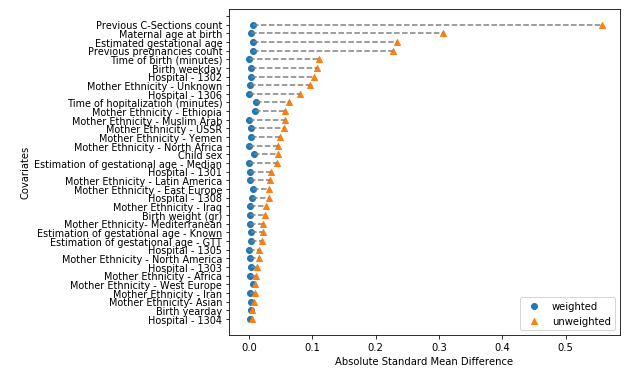 | 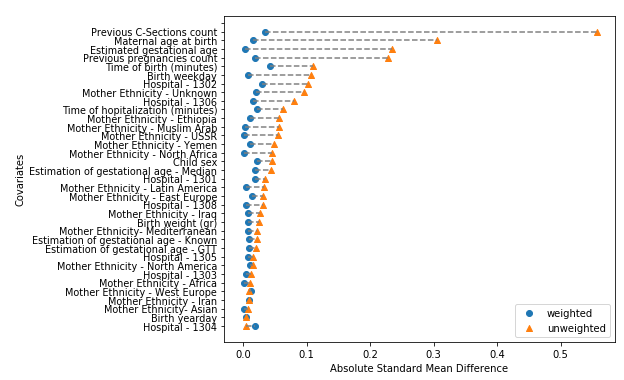 |
| --- | --- |
| **Figure S5.5: Covariates balance using Overlap Weights (OW) (left) or Inverse-Probability-weighting (IPW) (right).** | |

####

### 6. Expanded results and sensitivity analysis

| **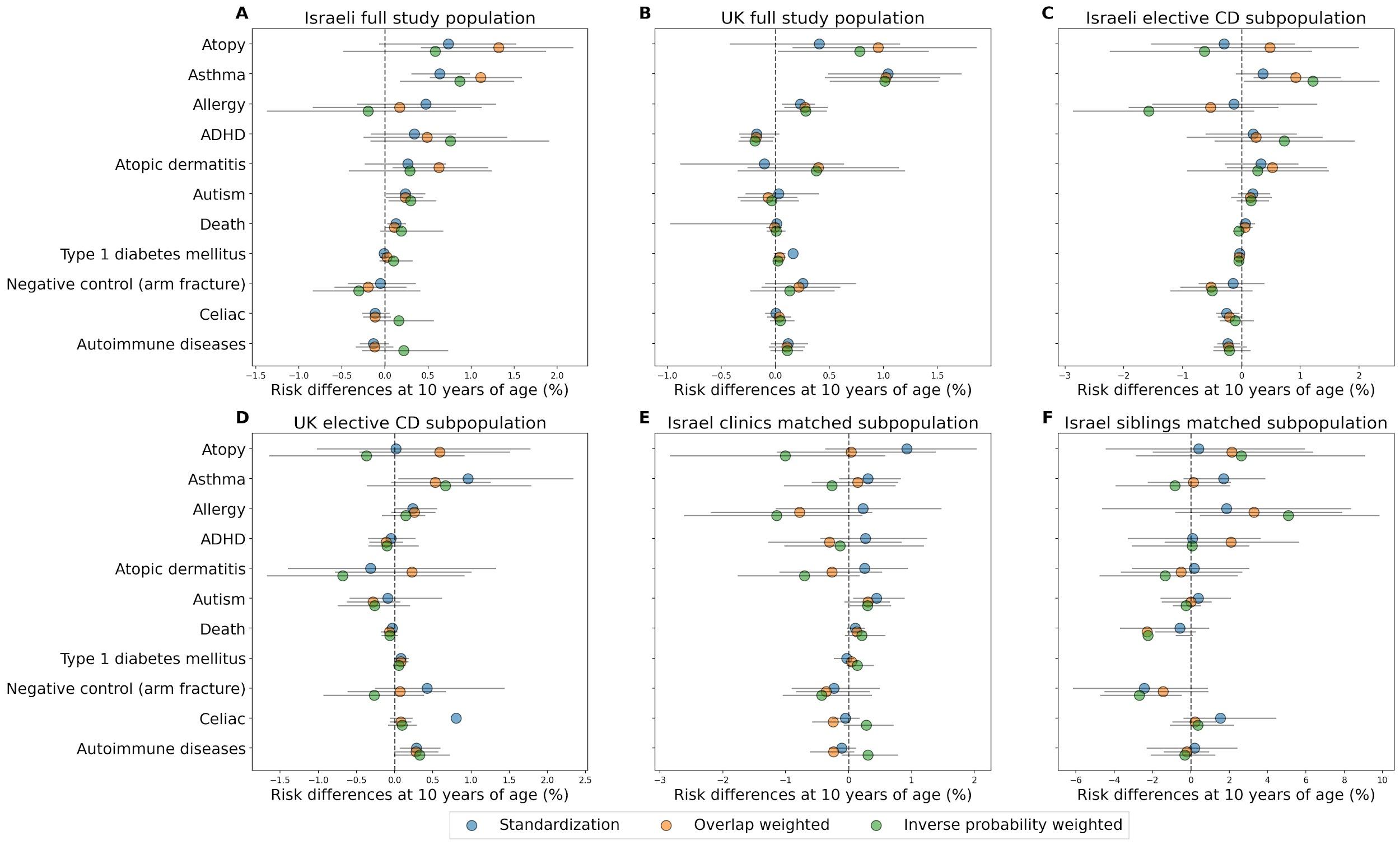**  **Figure S6.1: Estimated 10-yr risk difference (%) between children born vaginally or through CD.** Results are shown when using Standardization (blue circles), weighting with Overlap weights (orange circles) and Weighting with Inverse-Probability-Weighting (green circles), sorted by standardized risk differences in the Israel full cohort. Black lines represent 95% confidence intervals.  **A:** Israel full cohort results **B:** UK full cohort results **C:** Israel elective CD subpopulation results **D:** UK elective CD subpopulation results **E:** Israel clinics matched subpopulation results **F:** Israel siblings matched subpopulation results. |
| --- |

| **Israel cohort** | **UK cohort** |
| --- | --- |
| 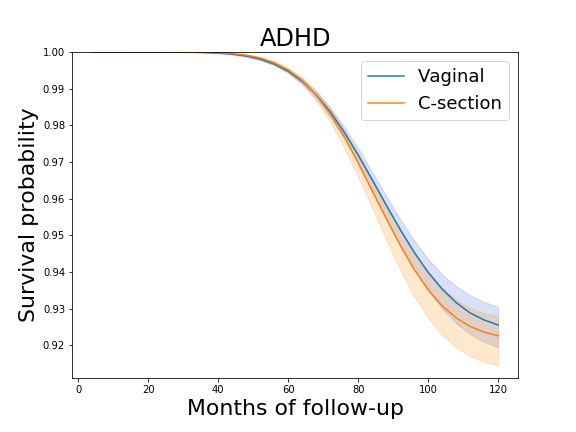 | 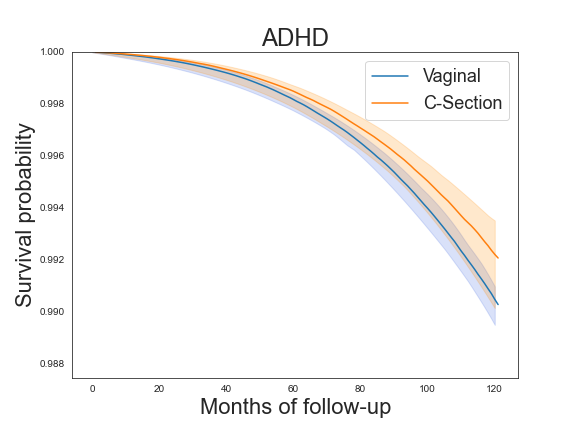 |
| 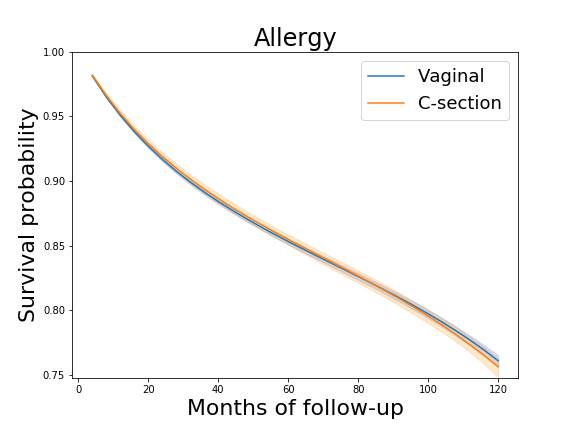 | 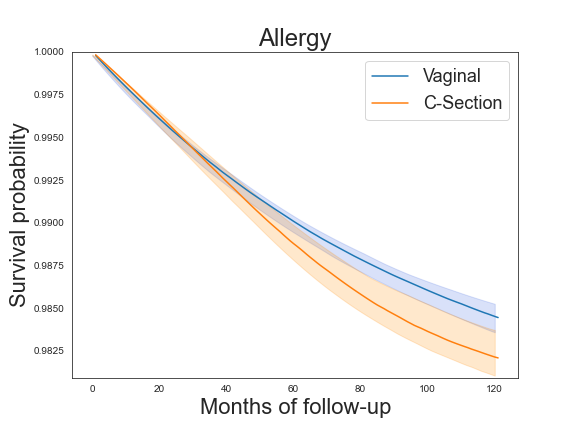 |
| 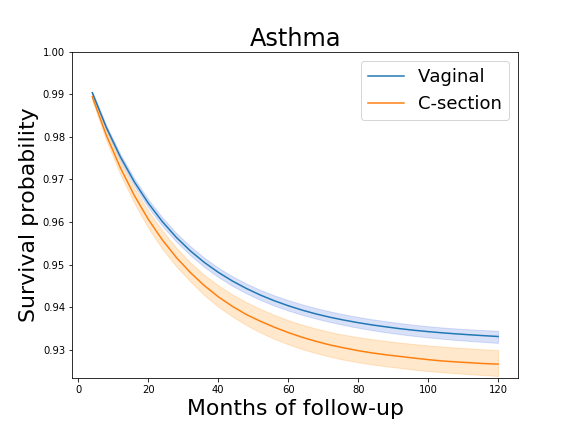 | 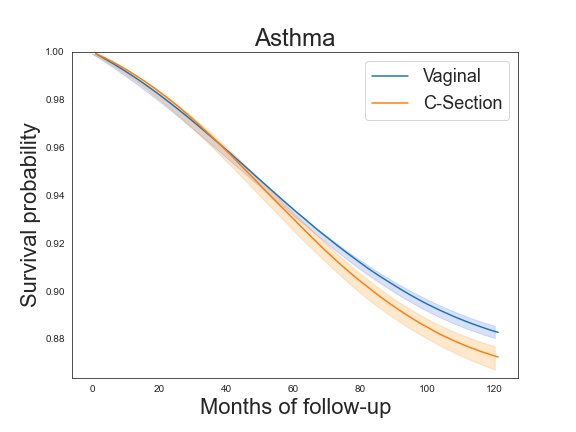 |
| 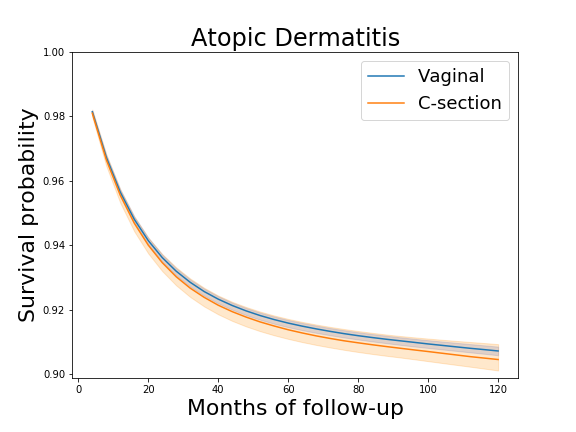 | 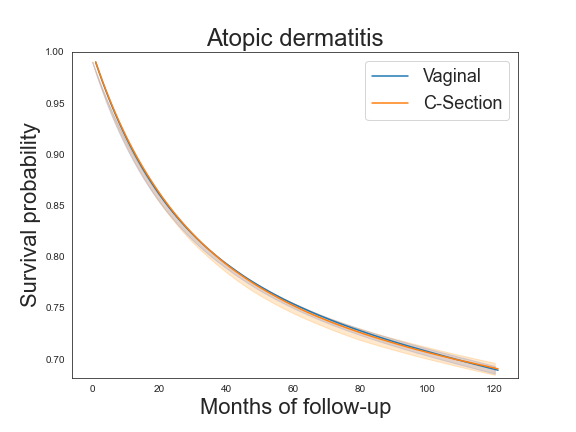 |
| 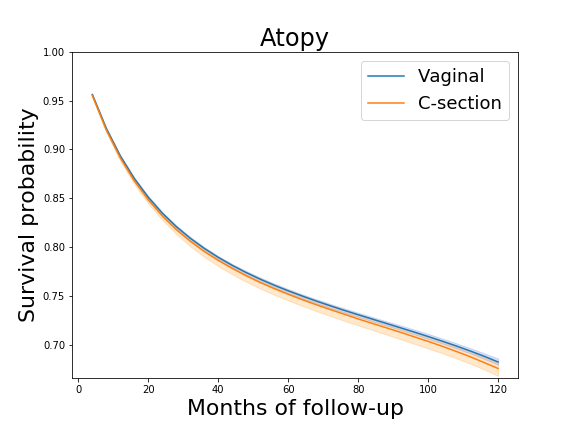 | 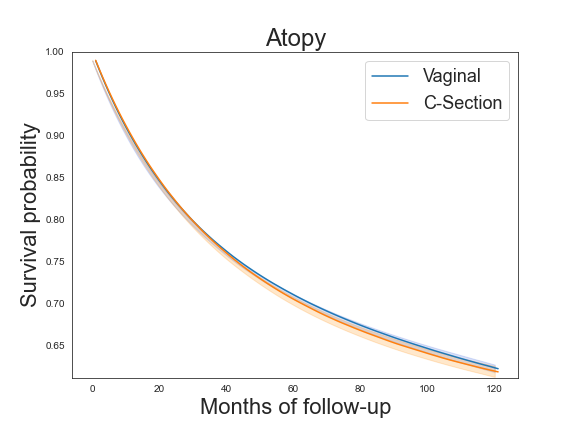 |
| 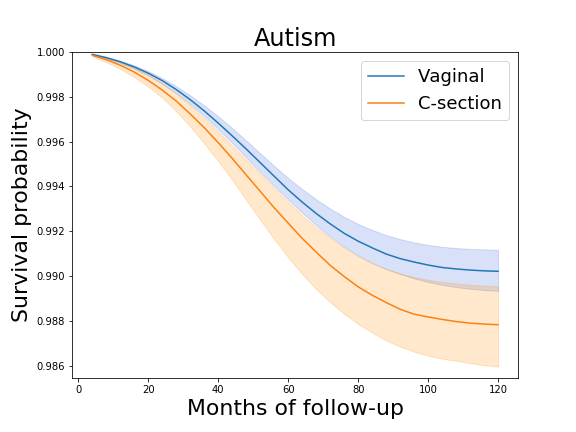 | 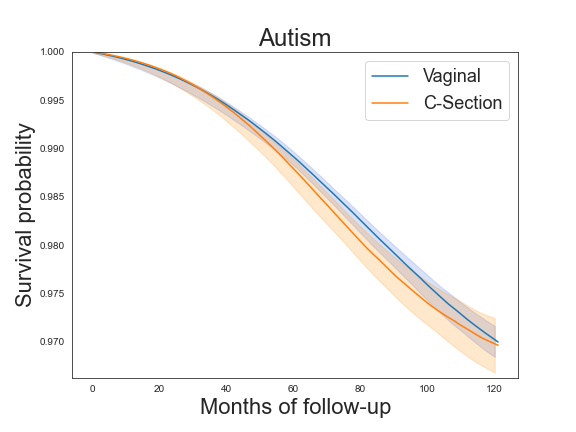 |
| 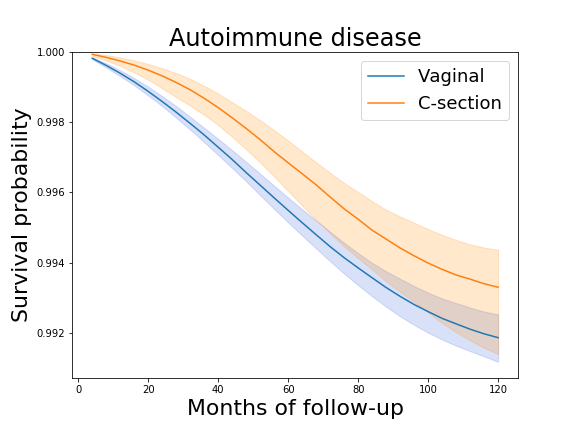 | 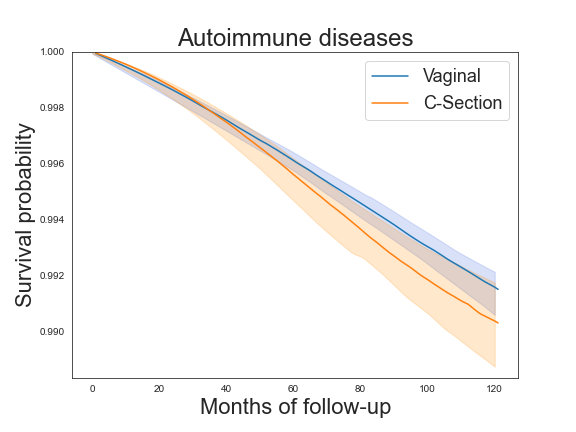 |
| 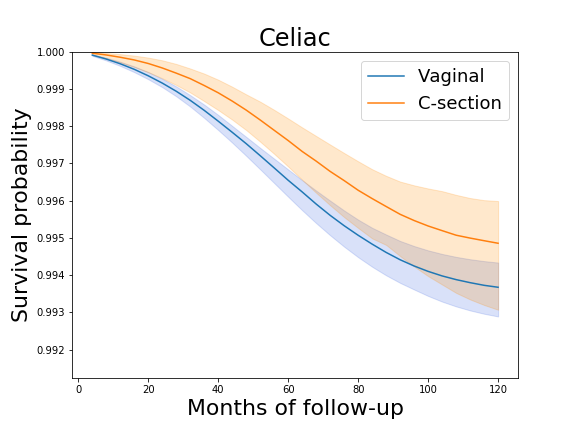 | 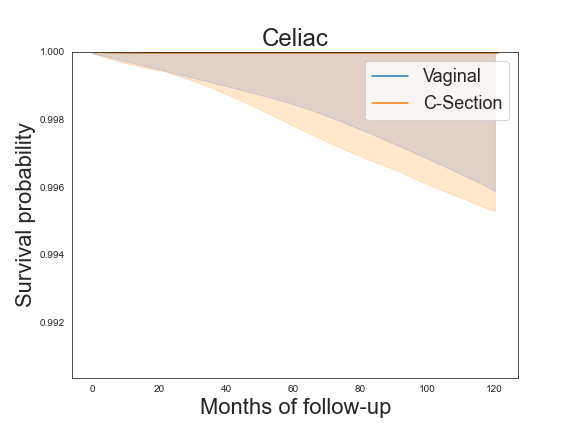 |
| 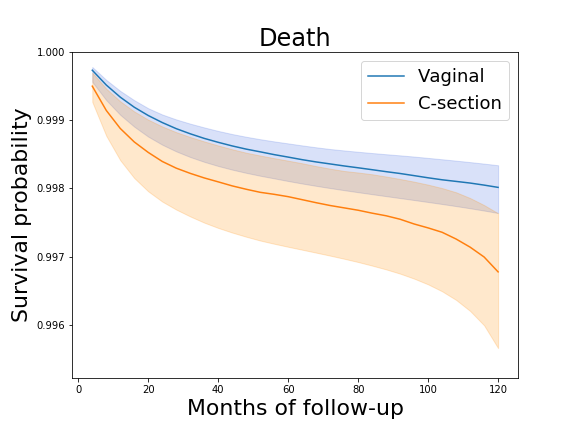 | 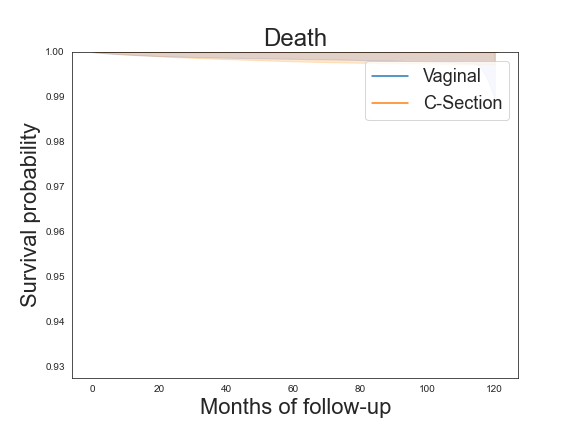 |
| 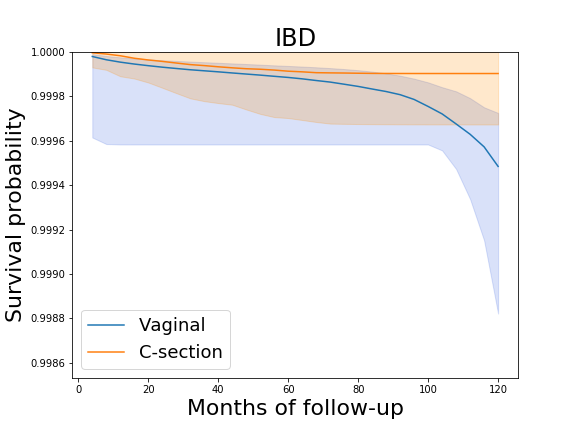 | 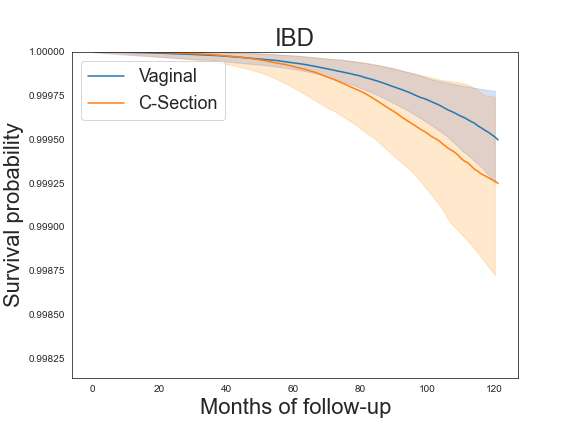 |
| 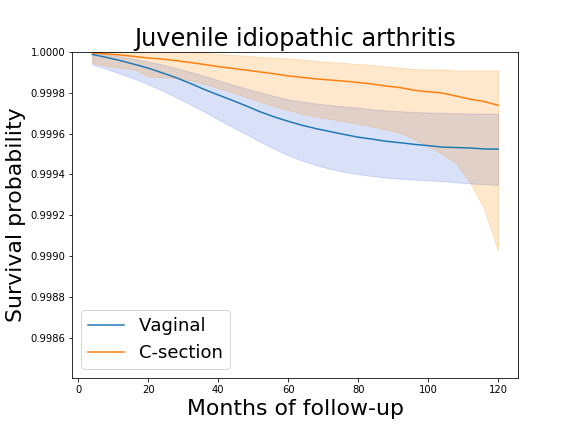 | X |
| 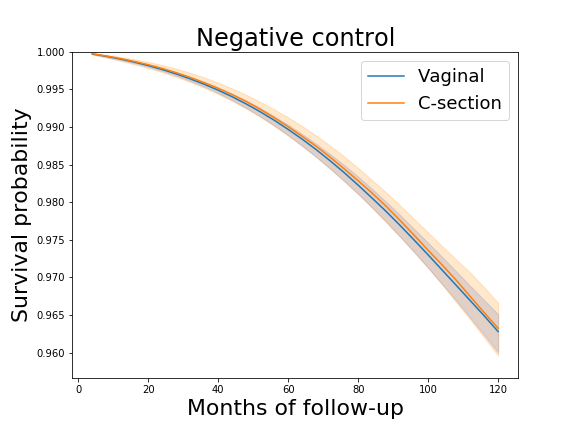 | 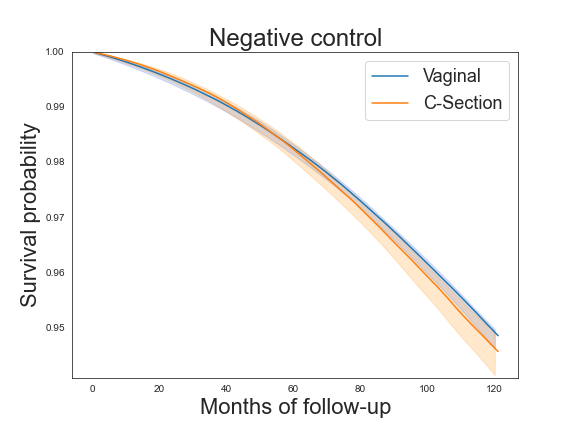 |
| 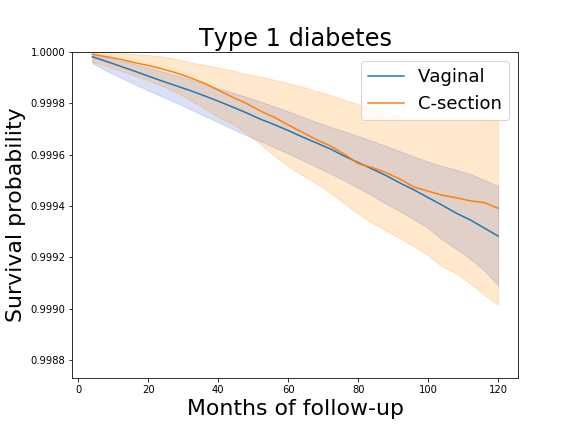 | 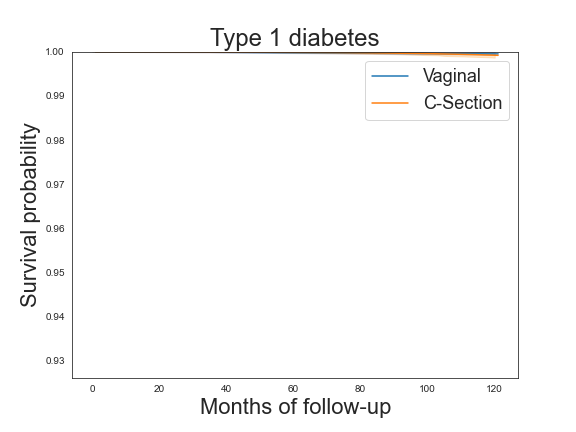 |
| **Figure S6.2: Childhood disease-free survival curves comparing vaginal and cesarean deliveries.** Standardized survival curves with 95% CI of children born through vaginal delivery (blue) and CD (orange) are shown by months of follow-up. **Left:** Israel cohort, full study population. **Right:** UK cohort, full study population. | |

| **Table S6.1 Sensitivity analysis of estimated 10-yr childhood disease-free risk differences for comparing vaginal and cesarean deliveries - standardized, OW, IPW, across two data sources** | | | | | | | | | | |
| --- | --- | --- | --- | --- | --- | --- | --- | --- | --- | --- |
| **Full study population** | | | | | | | | | | |
| **Childhood health outcome** | **Israel cohort** | | | | | **UK cohort** | | | | |
|  | **Number of incident cases** | | **10-yr risk**  **difference (95% CI)** | | | **Number of incident cases** | | **10-yr risk**  **difference (95% CI)** | | |
|  | **Vaginal**  **(n=200,879)** | **Cesarean**  **(n=37,280)** |  |  |  | **Vaginal**  **(n=125,743)** | **Cesarean**  **(n=37,529)** |  |  |  |
|  |  |  | **Standardized** | **OW** | **IPW** |  |  | **Standardized** | **OW** | **IPW** |
|  |  |  |  |  |  |  |  | **Standardized** | **OW** | **IPW** |
| ADHD | 6,119 (3.05%) | 1,485 (3.98%) | 0.34% (-0.16, 0.82) | 0.49% (-0.24, 1.41) | 0.76% (-0.16, 1.9) | 516 (0.41%) | 114 (0.3%) | -0.17% (-0.33, 0.03) | -0.18% (-0.32, -0.01) | -0.19% (-0.34, -0.03) |
| Allergy | 34,045 (16.95%) | 6,924 (18.57%) | 0.47% (-0.32, 1.28) | 0.17% (-0.84, 1.12) | -0.2% (-1.37, 0.82) | 1,270 (1.01%) | 467 (1.24%) | 0.23% (0.07, 0.36) | 0.27% (0.09, 0.48) | 0.28% (0.0, 0.47) |
| Asthma | 11,900 (5.92%) | 2,756 (7.39%) | 0.64% (0.31, 0.98) | 1.11% (0.52, 1.58) | 0.87% (0.18, 1.49) | 9,117 (7.25%) | 2,789 (7.43%) | 1.04% (0.49, 1.71) | 1.02% (0.46, 1.52) | 1.01% (0.51, 1.5) |
| Atopic dermatitis | 20,019 (9.97%) | 4,201 (11.27%) | 0.26% (-0.23, 0.7) | 0.62% (0.1, 1.19) | 0.29% (-0.41, 1.23) | 30,234 (24.04%) | 9,031 (24.06%) | -0.1% (-0.87, 0.63) | 0.4% (-0.25, 1.13) | 0.38% (-0.34, 1.19) |
| Atopy | 55,505 (27.63%) | 11,483 (30.8%) | 0.74% (-0.06, 1.52) | 1.32% (0.42, 2.18) | 0.58% (-0.48, 1.86) | 35,805 (28.47%) | 10,747 (28.64%) | 0.41% (-0.42, 1.15) | 0.95% (0.16, 1.85) | 0.78% (0.03, 1.41) |
| Autistic spectrum disorder | 1,212 (0.6%) | 400 (1.07%) | 0.24% (0.02, 0.46) | 0.23% (0.01, 0.43) | 0.3% (0.04, 0.59) | 1,887 (1.5%) | 592 (1.58%) | 0.03% (-0.27, 0.39) | -0.07% (-0.34, 0.2) | -0.04% (-0.32, 0.21) |
| Autoimmune diseases | 1,062 (0.53%) | 178 (0.48%) | -0.13% (-0.29, 0.04) | -0.12% (-0.34, 0.09) | 0.21% (-0.26, 0.73) | 597 (0.47%) | 200 (0.53%) | 0.12% (-0.04, 0.3) | 0.11% (-0.05, 0.27) | 0.11% (-0.04, 0.25) |
| Celiac | 784 (0.39%) | 123 (0.33%) | -0.12% (-0.26, 0.05) | -0.11% (-0.25, 0.06) | 0.16% (-0.15, 0.56) | 223 (0.18%) | 81 (0.22%) | 0.0% (-0.09, 0.09) | 0.04% (-0.07, 0.14) | 0.05% (-0.05, 0.17) |
| Death | 583 (0.29%) | 169 (0.45%) | 0.13% (0.03, 0.24) | 0.11% (0.01, 0.2) | 0.19% (-0.05, 0.67) | 209 (0.17%) | 73 (0.19%) | 0.01% (-0.97, 0.03) | -0.01% (-0.08, 0.05) | 0.01% (-0.07, 0.09) |
| Inflammatory bowel disease | 30 (0.01%) | 3 (0.01%) | -0.05% (-0.1, -0.01) | -0.03% (-0.06, 0.0) | -0.03% (-0.05, -0.01) | 25 (0.02%) | 10 (0.03%) | 0.03% (-0.02, 0.09) | 0.03% (-0.02, 0.08) | 0.02% (-0.02, 0.08) |
| Juvenile idiopathic arthritis | 55 (0.03%) | 8 (0.02%) | -0.01% (-0.04, 0.04) | -0.03% (-0.06, -0.01) | -0.03% (-0.05, -0.02) | - | - | - | - | - |
| Negative control (arm fracture) | 3,581 (1.78%) | 707 (1.9%) | -0.05% (-0.42, 0.35) | -0.2% (-0.58, 0.24) | -0.31% (-0.84, 0.4) | 3,278 (2.61%) | 951 (2.53%) | 0.26% (-0.09, 0.74) | 0.22% (-0.12, 0.6) | 0.13% (-0.23, 0.54) |
| Type 1 diabetes mellitus | 79 (0.04%) | 20 (0.05%) | -0.01% (-0.05, 0.03) | 0.02% (-0.05, 0.11) | 0.1% (-0.06, 0.31) | 33 (0.03%) | 14 (0.04%) | 0.16% (-0.01, 0.09) | 0.04% (-0.01, 0.09) | 0.03% (-0.02, 0.07) |
| **Elective CD matched subpopulation** | | | | | | | | | | |
| **Childhood health outcome** | **Israel cohort** | | | | | **UK cohort** | | | | |
|  | **Number of incident cases** | | **10-yr risk**  **difference (95% CI)** | | | **Number of incident cases** | | **10-yr risk**  **difference (95% CI)** | | |
|  | **Vaginal**  **(n=200,879)** | **Cesarean**  **(n=16,379)** |  |  |  | **Vaginal** | **Cesarean** |  |  |  |
|  |  |  | **Standardized** | **OW** | **IPW** |  |  | **Standardized** | **OW** | **IPW** |
| ADHD | 6,119 | 585 | -0.3% (-3.2, 2.1) | -0.4% (-3.6, 2.1) | -2.1% (-7.6, 2.9) |  |  | 0.9 (0.7, 1.2) | 1.0 (0.6, 1.3) | 0.9 (0.5, 1.4) |
| ADHD | 6,119 (3.05%) | 585 (3.57%) | 0.2% (-0.6, 0.92) | 0.24% (-0.92, 1.37) | 0.72% (-0.45, 1.91) | 516 (0.41%) | 53 (0.34%) | -0.05% (-0.34, 0.26) | -0.12% (-0.33, 0.1) | -0.1% (-0.34, 0.31) |
| Allergy | 34,045 (16.95%) | 2,898 (17.69%) | -0.13% (-1.51, 1.28) | -0.53% (-1.91, 0.61) | -1.57% (-2.86, 0.2) | 1,270 (1.01%) | 196 (1.27%) | 0.24% (0.02, 0.55) | 0.26% (-0.04, 0.53) | 0.15% (-0.16, 0.39) |
| Asthma | 11,900 (5.92%) | 1,194 (7.29%) | 0.37% (-0.09, 0.92) | 0.92% (0.21, 1.67) | 1.21% (0.05, 2.33) | 9,117 (7.25%) | 1,151 (7.44%) | 0.96% (0.05, 2.33) | 0.53% (-0.04, 1.25) | 0.66% (-0.36, 1.78) |
| Atopic dermatitis | 20,019 (9.97%) | 1,746 (10.66%) | 0.33% (-0.27, 0.95) | 0.52% (-0.24, 1.44) | 0.27% (-0.92, 1.47) | 30,234 (24.04%) | 3,668 (23.7%) | -0.32% (-1.39, 1.32) | 0.23% (-0.78, 0.99) | -0.68% (-1.67, 0.91) |
| Atopy | 55,505 (27.63%) | 4,861 (29.68%) | -0.29% (-1.53, 0.9) | 0.48% (-0.8, 1.99) | -0.63% (-2.23, 1.19) | 35,805 (28.47%) | 4,383 (28.32%) | 0.02% (-1.02, 1.77) | 0.59% (-0.46, 1.5) | -0.37% (-1.64, 0.91) |
| Autistic spectrum disorder | 1,212 (0.6%) | 178 (1.09%) | 0.19% (-0.05, 0.47) | 0.15% (-0.16, 0.5) | 0.16% (-0.08, 0.45) | 1,887 (1.5%) | 249 (1.61%) | -0.09% (-0.58, 0.61) | -0.29% (-0.62, 0.06) | -0.26% (-0.74, 0.19) |
| Autoimmune diseases | 1,062 (0.53%) | 78 (0.48%) | -0.23% (-0.4, -0.04) | -0.22% (-0.46, 0.08) | -0.21% (-0.47, 0.14) | 597 (0.47%) | 100 (0.65%) | 0.28% (0.07, 0.59) | 0.28% (-0.0, 0.56) | 0.33% (-0.0, 0.71) |
| Celiac | 784 (0.39%) | 52 (0.32%) | -0.26% (-0.42, -0.05) | -0.2% (-0.39, -0.0) | -0.11% (-0.36, 0.2) | 223 (0.18%) | 43 (0.28%) | 0.8% (-0.06, 0.23) | 0.08% (-0.06, 0.21) | 0.1% (-0.08, 0.28) |
| Death | 583 (0.29%) | 66 (0.4%) | 0.07% (-0.04, 0.22) | 0.06% (-0.05, 0.18) | -0.05% (-0.14, 0.05) | 209 (0.17%) | 23 (0.15%) | -0.03% (-0.1, 0.0) | -0.07% (-0.18, 0.03) | -0.07% (-0.17, 0.03) |
| Inflammatory bowel disease | 30 (0.01%) | 0 (0.0%) | -0.05% (-0.1, -0.02) | -0.04% (-0.06, -0.02) | -0.04% (-0.06, -0.02) | 25 (0.02%) | 6 (0.04%) | 0.34% (-0.03, 0.19) | 0.06% (-0.01, 0.15) | 0.02% (-0.03, 0.09) |
| Juvenile idiopathic arthritis | 55 (0.03%) | 3 (0.02%) | -0.02% (-0.06, 0.01) | -0.04% (-0.06, -0.02) | -0.03% (-0.05, -0.01) | - | - | - | - | - |
| Negative control (arm fracture) | 3,581 (1.78%) | 306 (1.87%) | -0.14% (-0.72, 0.38) | -0.52% (-1.04, -0.0) | -0.5% (-1.2, 0.18) | 3,278 (2.61%) | 418 (2.7%) | 0.42% (-0.25, 1.43) | 0.07% (-0.61, 0.66) | -0.27% (-0.93, 0.37) |
| Type 1 diabetes mellitus | 79 (0.04%) | 8 (0.05%) | -0.03% (-0.06, 0.01) | -0.04% (-0.09, 0.0) | -0.04% (-0.09, -0.01) | 33 (0.03%) | 9 (0.06%) | 0.08% (0.0, 0.17) | 0.08% (0.01, 0.17) | 0.05% (-0.02, 0.13) |

ADHD - Attention deficit hyperactivity Disorder, ATE - Average Treatment Effect, OW - Overlap Weights, IPW - Inverse Probability Weighting

| **Table S6.2 Sensitivity analysis of estimated 10-yr childhood disease-free risk differences for comparing vaginal and cesarean deliveries - standardized, OW, IPW** | | | | | |
| --- | --- | --- | --- | --- | --- |
| **Clinics matched subpopulation** | | | | | |
| **Childhood health outcome** | **Number of incident cases** | | **10-yr risk**  **difference (95% CI)** | | |
|  | **Vaginal**  **(n=33,232)** | **Cesarean**  **(n=33,232)** |  |  |  |
|  |  |  | **Standardized** | **OW** | **IPW** |
| ADHD | 1,207 (3.48%) | 1,343 (4.03%) | 0.27% (-0.45, 1.24) | -0.31% (-1.27, 0.83) | -0.13% (-1.01, 1.19) |
| Allergy | 5,869 (16.92%) | 6,110 (18.35%) | 0.23% (-1.15, 1.47) | -0.78% (-2.18, 0.37) | -1.14% (-2.61, 0.21) |
| Asthma | 2,234 (6.44%) | 2,437 (7.32%) | 0.31% (-0.14, 0.82) | 0.14% (-0.58, 0.77) | -0.27% (-1.02, 0.74) |
| Atopic dermatitis | 3,614 (10.42%) | 3,752 (11.27%) | 0.25% (-0.33, 0.93) | -0.27% (-1.09, 0.52) | -0.7% (-1.76, 0.17) |
| Atopy | 9,757 (28.13%) | 10,207 (30.66%) | 0.93% (-0.36, 2.02) | 0.04% (-1.13, 1.38) | -1.01% (-2.83, 0.57) |
| Autistic spectrum disorder | 209 (0.6%) | 368 (1.11%) | 0.44% (0.08, 0.88) | 0.31% (-0.06, 0.65) | 0.3% (0.02, 0.66) |
| Autoimmune diseases | 183 (0.53%) | 166 (0.5%) | -0.11% (-0.31, 0.1) | -0.24% (-0.6, 0.08) | 0.3% (-0.11, 0.78) |
| Celiac | 136 (0.39%) | 112 (0.34%) | -0.05% (-0.26, 0.16) | -0.25% (-0.57, 0.01) | 0.28% (-0.07, 0.7) |
| Death | 81 (0.23%) | 151 (0.45%) | 0.1% (-0.02, 0.25) | 0.13% (-0.01, 0.28) | 0.21% (-0.05, 0.57) |
| Inflammatory bowel disease | 4 (0.01%) | 3 (0.01%) | 0.0% (-0.06, 0.07) | -6.0% (-99.9, 0.02) | -11.01% (-100.0, 0.01) |
| Juvenile idiopathic arthritis | 8 (0.02%) | 8 (0.02%) | -0.03% (-0.17, 0.07) | -0.02% (-0.04, 0.01) | -0.02% (-0.04, 0.0) |
| Negative control (arm fracture) | 632 (1.82%) | 628 (1.89%) | -0.23% (-0.9, 0.48) | -0.36% (-0.83, 0.32) | -0.43% (-1.04, 0.36) |
| Type 1 diabetes mellitus | 14 (0.04%) | 20 (0.06%) | -0.04% (-0.23, 0.07) | 0.04% (-0.04, 0.14) | 0.14% (-0.03, 0.39) |
| **Siblings matched subpopulation** | | | | | |
| **Childhood health outcome** | **Number of incident cases** | | **10-yr risk**  **difference (95% CI)** | | |
|  | **Vaginal**  **(n=1,968)** | **Cesarean**  **(n=1,968)** |  |  |  |
|  |  |  | **Standardized** | **OW** | **IPW** |
| ADHD | 59 (2.53%) | 73 (3.07%) | 0.08% (-3.27, 3.61) | 2.08% (-1.35, 5.6) | 0.07% (-3.05, 3.02) |
| Allergy | 316 (13.56%) | 362 (15.21%) | 1.85% (-4.62, 8.32) | 3.29% (-0.8, 7.86) | 5.08% (0.48, 9.81) |
| Asthma | 131 (5.62%) | 136 (5.71%) | 1.7% (-0.37, 3.84) | 0.12% (-2.22, 2.05) | -0.84% (-3.91, 2.01) |
| Atopic dermatitis | 211 (9.06%) | 219 (9.2%) | 0.17% (-3.06, 3.01) | -0.51% (-3.63, 2.65) | -1.34% (-4.75, 2.4) |
| Atopy | 560 (24.03%) | 590 (24.79%) | 0.4% (-4.43, 5.92) | 2.14% (-1.96, 6.34) | 2.61% (-2.84, 9.03) |
| Autistic spectrum disorder | 13 (0.56%) | 15 (0.63%) | 0.38% (-1.56, 2.05) | -0.01% (-1.5, 1.03) | -0.27% (-0.92, 0.5) |
| Autoimmune diseases | 16 (0.69%) | 9 (0.38%) | 0.19% (-2.29, 2.38) | -0.23% (-1.4, 0.9) | -0.33% (-2.08, 1.24) |
| Celiac | 11 (0.47%) | 7 (0.29%) | 1.52% (-0.37, 4.43) | 0.2% (-0.95, 1.26) | 0.37% (-1.07, 2.21) |
| Death | 8 (0.34%) | 14 (0.59%) | -0.58% (-3.68, 0.92) | -2.29% (-1.84, 0.24) | -2.24% (-0.78, 0.07) |
| Inflammatory bowel disease | - | - | - | - | - |
| Juvenile idiopathic arthritis | - | - | - | - | - |
| Negative control (arm fracture) | 43 (1.85%) | 33 (1.39%) | -2.43% (-6.13, 0.85) | -1.46% (-4.49, 0.87) | -2.69% (-4.71, -0.52) |
| Type 1 diabetes mellitus | - | - | - | - | - |
| **Subpopulation of women with no history of a previous CD** | | | | | |
| **Childhood health outcome** | **Number of incident cases** | | **10-yr risk**  **difference (95% CI)** | | |
|  | **Vaginal**  **(n=194,275)** | **Cesarean**  **(n=25,766)** |  |  |  |
|  |  |  | **Standardized** | **OW** | **IPW** |
| ADHD | 5,955 (3.06%) | 1,043 (4.04%) | 0.22% (-0.28, 0.89) | 0.18% (-0.66, 0.85) | 0.59% (-0.36, 1.57) |
| Allergy | 33,073 (17.02%) | 5,038 (19.55%) | 0.24% (-0.88, 0.94) | 0.07% (-0.86, 1.05) | 0.17% (-0.87, 1.51) |
| Asthma | 11,516 (5.93%) | 1,920 (7.45%) | 0.82% (0.48, 1.15) | 1.17% (0.59, 1.97) | 1.00% (0.08, 1.76) |
| Atopic dermatitis | 19,410 (9.99%) | 3,088 (11.98%) | 0.48% (-0.02, 0.97) | 0.96% (0.28, 1.64) | 0.52% (-0.37, 1.32) |
| Atopy | 53,819 (27.7%) | 8,257 (32.04%) | 0.89% (0.15, 1.8) | 1.31% (0.44, 2.2) | 1.01% (-0.27, 2.62) |
| Autistic spectrum disorder | 1,184 (0.61%) | 310 (1.2%) | 0.24% (0.09, 0.46) | 0.26% (0.05, 0.5) | 0.33% (0.04, 0.69) |
| Autoimmune diseases | 1,017 (0.52%) | 112 (0.43%) | -0.13% (-0.3, 0.02) | -0.09% (-0.4, 0.1) | 0.15% (-0.18, 0.72) |
| Celiac | 746 (0.38%) | 80 (0.31%) | -0.06% (-0.2, 0.11) | -0.08% (0.07, 0.11) | 0.15% (-0.13, 0.53) |
| Death | 552 (0.28%) | 121 (0.47%) | 0.09% (0.01, 0.18) | 0.07% (-0.02, 0.2) | 0.13% (-0.08, 0.44) |
| Inflammatory bowel disease | 30 (0.02%) | 3 (0.01%) | -0.03% (-0.08, 0.0) | -0.03% (-0.06, 0.0) | -0.03% (-0.05, -0.02) |
| Juvenile idiopathic arthritis | 53 (0.03%) | 3 (0.01%) | -0.04% (-0.06, -0.02) | -0.05% (-0.07, -0.03) | -0.04% (-0.05, -0.03) |
| Negative control (arm fracture) | 3,480 (1.8%) | 481 (1.87%) | -0.14% (-0.47, 0.33) | -0.23% (-0.85, 0.24) | -0.48% (-0.95, 0.16) |
| Type 1 diabetes mellitus | 76 (0.04%) | 10 (0.04%) | 0.0 (-0.05, 0.07) | 0.02% (-0.04, 0.13) | 0.1% (-0.07, 0.34) |

ADHD - Attention deficit hyperactivity Disorder, ATE - Average Treatment Effect, OW - Overlap Weights, IPW - Inverse Probability Weighting

| **Table S6.3 Estimated childhood obesity, antibiotics intake and clinic visits differences for comparing vaginal and cesarean deliveries, across two data sources** | | | | | | | | | | |
| --- | --- | --- | --- | --- | --- | --- | --- | --- | --- | --- |
| **Full study population** | | | | | | | | | | |
| **Childhood health outcome** | **Israel cohort** | | | | | **UK cohort** | | | | |
|  | **Number of children with sufficient data** | | **ATE difference** | | | **Number of children with sufficient data** | | **ATE difference** | | |
|  | **Vaginal** | **Cesarean** |  |  |  | **Vaginal** | **Cesarean** |  |  |  |
|  |  |  | **Standardized** | **OW** | **IPW** |  |  | **Standardized** | **OW** | **IPW** |
| BMI z-score at age 5-6 years old | 99,345 (83.6%) | 19,454 (16.4%) | 0.10 (0.07, 0.12) | 0.10 (0.07, 0.12) | 0.22 (0.17, 0.29) | - | - | - | - | - |
| Respiratory infections - number of incidents until 5 years of age | 107,654 (83.8%) | 20,864 (16.2%) | 0.92 (0.68, 1.14) | 0.96 (0.72, 1.21) | 1.43 (0.95, 1.89) | 70,705 (78.24%) | 19,666 (21.76%) | 0.12 (0.08, 0.15) | 0.14 (0.10, 0.17) | 0.14 (0.11 ,0.17) |
| **Elective CD subpopulation** | | | | | |  |  |  |  |  |
| BMI z-score at age 5-6 years old | 99,345 (92.5%) | 8,058 (7.5%) | 0.10 (0.08, 0.12) | 0.12 (0.08, 0.17) | 0.22 (0.01, 0.38) | - | - | - | - | - |
| Respiratory infections - number of incidents until 5 years of age | 107,654 (92.6%) | 8,577 (7.4%) | 1.11 (0.91, 1.35) | 1.02 (0.62, 1.44) | 1.63 (0.24, 3.81) | 70,705  (89.63%) | 8,181  (10.37%) | 0.10 (0.06, 0.15) | 0.12 (0.07, 0.17) | 0.11 (0.06 ,0.16) |

BMI - Body Mass Index, ATE - Average Treatment Effect, OW - Overlap Weights, IPW - Inverse Probability Weighting

BMI z-score calculated according to 2000 CDC Growth Charts for the United States [[25]](https://sciwheel.com/work/citation?ids=1111621&pre=&suf=&sa=0)

| **Table S6.4 Estimated childhood obesity, antibiotics intake and clinic visits differences for comparing vaginal and cesarean deliveries** | | | | | |
| --- | --- | --- | --- | --- | --- |
| **Clinics matched subpopulation** | | | | | |
| **Childhood health outcome** | **Number of children with sufficient data** | | **ATE difference** | | |
|  | **Vaginal** | **Cesarean** |  |  |  |
|  |  |  | **Standardized** | **OW** | **IPW** |
| BMI z-score at age 5-6 years old | 17,122 (49.5%) | 17,478 (50.5%) | 0.09 (0.07, 0.12) | 0.09 (0.06, 0.12) | 0.22 (0.11, 0.35) |
| Respiratory infections - number of incidents until 5 years of age | 18,832 (50.0%) | 18,851 (50.0%) | 0.96 (0.76, 1.17) | 0.82 (0.52, 1.09) | 1.46 (0.58, 2.25) |
| **Siblings matched subpopulation** | | | | | |
| BMI z-score at age 5-6 years old | 1,022 (48.4%) | 1,089 (51.6%) | 0.10 (0.07, 0.12) | 0.10 (0.07, 0.13) | 0.22 (0.18, 0.27) |
| Respiratory infections - number of incidents until 5 years of age | 1,133 (49.5%) | 1,155 (50.5%) | 1.02 (0.81, 1.25) | 0.97 (0.73, 1.19) | 1.43 (0.99, 1.80) |
| **No previous CD subpopulation** | | | | | |
| BMI z-score at age 5-6 years old | 96,031 (87.7%) | 13,441 (12.3%) | 0.09 (0.07, 0.12) | 0.09 (0.07, 0.12) | 0.22 (0.18, 0.26) |
| Respiratory infections - number of incidents until 5 years of age | 104,048 (87.9%) | 14,337 (12.1%) | 0.92 (0.70, 1.16) | 1.00 (0.72, 1.23) | 1.74 (1.23, 2.34) |

BMI - Body Mass Index, ATE - Average Treatment Effect, OW - Overlap Weights, IPW - Inverse Probability Weighting

BMI z-score calculated according to 2000 CDC Growth Charts for the United States [[25]](https://sciwheel.com/work/citation?ids=1111621&pre=&suf=&sa=0)

**Elective CD subpopulation**

| **Table S6.5 Baseline characteristics of the elective CD subpopulation - Israel cohort** | | | |
| --- | --- | --- | --- |
| **Characteristic, mean (SD) or counts %** | **Vaginal (n = 200,879) (92.5%)** | **Cesarean (n = 16,379) (7.5%)** | **All (n = 217,258)** |
| **Neonatal characteristics** | | | |
| Sex - Male | 102,717 (51.13%) | 8,633 (52.71%) | 111,350 (51.25%) |
| Birth weight (gr) | 3,291.02 (391.48) | 3,277.03 (451.44) | 3,289.96 (396.33) |
| Gestational age at delivery (weeks) | 39.32 (1.14) | 38.13 (0.79) | 39.23 (1.16) |
| **Maternal characteristics** | | | |
| Maternal age (years) | 29.78 (5.34) | 33.12 (5.16) | 30.03 (5.4) |
| Maternal weight pre-pregnancy (Kg) | 62.37 (12.78) | 68.54 (15.93) | 62.86 (13.17) |
| Maternal height (meters) | 1.62 (0.06) | 1.62 (0.07) | 1.62 (0.06) |
| Diabetes Mellitus | 2,461 (1.23%) | 520 (3.17%) | 2,981 (1.37%) |
| Chronic Hypertension | 1,642 (0.82%) | 443 (2.7%) | 2,085 (0.96%) |
| Gravidity | 0.77 (0.81) | 0.63 (0.62) | 0.76 (0.8) |
| Previous cesarean deliveries | 6,604 (3.29%) | 7,123 (43.49%) | 13,727 (6.32%) |
| Sister’s cesarean deliveries, percent* | 12.25 (27.98) | 19.24 (35.12) | 12.66 (28.5) |
| **Pregnancy characteristics** | | | |
| Gestational Diabetes | 9,439 (4.7%) | 1,785 (10.9%) | 11,224 (5.17%) |
| Gestational Hypertension | 1,867 (0.93%) | 253 (1.54%) | 2,120 (0.98%) |
| Gestational weight gain z-score | -0.51 (2.42) | -0.36 (2.54) | -0.49 (2.43) |

Gestational weight gain z-score was calculated according to [[39]](https://sciwheel.com/work/citation?ids=8196823&pre=&suf=&sa=0)

*Sisters CD percent was calculated as the percentage of CDs over all sister’s pregnancies which ended prior to the relevant date of birth.

| **Table S6.6 Baseline characteristics of the elective CD subpopulation - UK cohort** | | | |
| --- | --- | --- | --- |
| **Characteristic, mean (SD) or counts %** | **Vaginal (n = 125,743) (89.04%)** | **Cesarean (n = 15,479) (10.96%)** | **All (n =141,222)** |
| **Neonatal characteristics** | | | |
| Sex - Male | 63,755 (50.70%) | 7,964 (51.45%) | 71,719 (50.78%) |
| Birth weight (gr) (N=58,695) | 3,478 (453) | 3,473 (487) | 3,478 (457) |
| Gestational age at delivery (weeks) (N=137,011) | 40.24 (1.28) | 39.31 (1.20) | 40.15 (1.30) |
| **Maternal characteristics** | | | |
| Maternal age (years) | 30.32 (5.59) | 32.92 (5.15) | 30.61 (5.57) |
| Maternal weight pre-pregnancy (Kg) (N=44,520) | 69.09 (16.36) | 73.68 (18.32) | 69.60 (16.65) |
| Maternal height (meters) (N=133,343) | 1.65 (0.07) | 1.63 (0.07) | 1.64 (0.07) |
| Diabetes Mellitus | 730 (0.58%) | 328 (2.12%) | 1058 (0.75%) |
| Chronic Hypertension | 528 (0.42%) | 138 (0.89%) | 666 (0.47%) |
| Gravidity | 0.82 (1.21) | 1.01 (1.28) | 0.84 (1.22) |
| Previous cesarean deliveries | 3206 (2.55%) | 5085 (32.85%) | 8291 (5.87%) |
| Sister’s cesarean deliveries, percent* | - | - | - |
| **Pregnancy characteristics** | | | |
| Gestational Diabetes | 1699 (1.35%) | 483 (3.12%) | 2182 (1.55%) |
| Gestational Hypertension | 1,248 (0.99%) | 243 (1.57%) | 1491 (1.06%) |
| Gestational weight gain z-score | - | - | - |

Gestational weight gain z-score was calculated according to [[39]](https://sciwheel.com/work/citation?ids=8196823&pre=&suf=&sa=0)

*Sisters CD percent was calculated as the percentage of CDs over all sister’s pregnancies which ended prior to the relevant date of birth.

**Clinics matched subpopulation**

| **Table S6.7 Baseline characteristics of the clinics matched subpopulation** | | | |
| --- | --- | --- | --- |
| **Characteristic, mean (SD) or counts %** | **Vaginal (n = 33,232) (50.0%)** | **Cesarean (n = 33,232) (50.0%)** | **All (n = 66,464)** |
| **Neonatal characteristics** | | | |
| Sex - Male | 16,910 (50.88%) | 18,072 (54.38%) | 34,982 (52.63%) |
| Birth weight (gr) | 3,294.54 (392.77) | 3,306.15 (460.67) | 3,300.34 (428.11) |
| Gestational age at delivery (weeks) | 39.29 (1.14) | 38.73 (1.24) | 39.01 (1.22) |
| **Maternal characteristics** | | | |
| Maternal age (years) | 30.2 (5.14) | 32.12 (5.4) | 31.16 (5.36) |
| Maternal weight pre-pregnancy (Kg) | 62.46 (12.87) | 66.94 (15.57) | 64.78 (14.5) |
| Maternal height (meters) | 1.62 (0.06) | 1.61 (0.07) | 1.62 (0.06) |
| Diabetes Mellitus | 423 (1.27%) | 837 (2.52%) | 1,260 (1.9%) |
| Chronic Hypertension | 289 (0.87%) | 725 (2.18%) | 1,014 (1.53%) |
| Gravidity | 0.72 (0.78) | 0.54 (0.65) | 0.63 (0.72) |
| Previous cesarean deliveries | 1,063 (3.2%) | 10,433 (31.39%) | 11,496 (17.3%) |
| Sister’s cesarean deliveries, percent* | 12.94 (29.29) | 18.75 (34.37) | 15.7 (31.93) |
| **Pregnancy characteristics** | | | |
| Gestational Diabetes | 1,655 (4.98%) | 3,207 (9.65%) | 4,862 (7.32%) |
| Gestational Hypertension | 336 (1.01%) | 520 (1.56%) | 856 (1.29%) |
| Gestational weight gain z-score | -0.47 (2.42) | -0.35 (2.56) | -0.4 (2.5) |

Gestational weight gain z-score was calculated according to [[39]](https://sciwheel.com/work/citation?ids=8196823&pre=&suf=&sa=0)

*Sisters CD percent was calculated as the percentage of CDs over all sister’s pregnancies which ended prior to the relevant date of birth.

**Siblings matched subpopulation**

| **Table S6.8 Baseline characteristics of the siblings matched subpopulation** | | | |
| --- | --- | --- | --- |
| **Characteristic, mean (SD) or counts %** | **Vaginal (n = 1,968) (50.0%)** | **Cesarean (n = 1,968) (50.0%)** | **All (n = 3,936)** |
| **Neonatal characteristics** | | | |
| Sex - Male | 1,119 (56.86%) | 1,119 (56.86%) | 2,238 (56.86%) |
| Birth weight (gr) | 3,314.51 (395.12) | 3,286.42 (468.41) | 3,300.47 (433.49) |
| Gestational age at delivery (weeks) | 39.4 (1.15) | 39.04 (1.28) | 39.22 (1.23) |
| **Maternal characteristics** | | | |
| Maternal age (years) | 29.99 (4.93) | 29.95 (5.42) | 29.97 (5.18) |
| Maternal weight pre-pregnancy (Kg) | 63.5 (13.44) | 63.55 (13.51) | 63.53 (13.47) |
| Maternal height (meters) | 1.61 (0.06) | 1.61 (0.07) | 1.61 (0.06) |
| Diabetes Mellitus | 36 (1.83%) | 31 (1.58%) | 67 (1.7%) |
| Chronic Hypertension | 25 (1.27%) | 17 (0.86%) | 42 (1.07%) |
| Gravidity | 0.94 (0.66) | 0.65 (0.78) | 0.8 (0.74) |
| Previous cesarean deliveries | 1,061 (53.91%) | 24 (1.22%) | 1,085 (27.57%) |
| Sister’s cesarean deliveries, percent* | 14.25 (30.57) | 15.83 (31.38) | 15.03 (30.97) |
| **Pregnancy characteristics** | | | |
| Gestational Diabetes | 99 (5.03%) | 153 (7.77%) | 252 (6.4%) |
| Gestational Hypertension | 20 (1.02%) | 22 (1.12%) | 42 (1.07%) |
| Gestational weight gain z-score | -0.56 (2.55) | -0.43 (2.62) | -0.48 (2.59) |

Gestational weight gain z-score was calculated according to [[39]](https://sciwheel.com/work/citation?ids=8196823&pre=&suf=&sa=0)

*Sisters CD percent was calculated as the percentage of CDs over all sister’s pregnancies which ended prior to the relevant date of birth.

**No previous CD subpopulation**

| **Table S6.9 Baseline characteristics of the no previous CD exclusion subpopulation** | | | |
| --- | --- | --- | --- |
| **Characteristic, mean (SD) or counts %** | **Vaginal (n = 194,275) (%)** | **Cesarean (n = 25,766) (%)** | **All (n = 220,041)** |
| **Neonatal characteristics** | | | |
| Sex - Male | 194,275 (88.3%) | 25,766 (11.7%) | 220,041 |
| Birth weight (gr) | 99,388 (51.16%) | 14,192 (55.08%) | 113,580 (51.62%) |
| Gestational age at delivery (weeks) | 3,291.82 (391.57) | 3,315.01 (470.94) | 3,294.54 (401.74) |
| **Maternal characteristics** | | | |
| Maternal age (years) | 29.77 (5.36) | 31.96 (5.7) | 30.03 (5.44) |
| Maternal weight pre-pregnancy (Kg) | 62.33 (12.77) | 66.21 (15.33) | 62.82 (13.18) |
| Maternal height (meters) | 1.62 (0.06) | 1.62 (0.07) | 1.62 (0.06) |
| Diabetes Mellitus | 2,243 (1.15%) | 464 (1.8%) | 2,707 (1.23%) |
| Chronic Hypertension | 1,558 (0.8%) | 465 (1.8%) | 2,023 (0.92%) |
| Gravidity | 0.75 (0.81) | 0.28 (0.59) | 0.69 (0.8) |
| Previous cesarean deliveries | 0 (0.0%) | 0 (0.0%) | 0 (0.0%) |
| Sister’s cesarean deliveries, percent* | 12.17 (27.95) | 18.17 (34.07) | 12.71 (28.6) |
| **Pregnancy characteristics** | | | |
| Gestational Diabetes | 9,151 (4.71%) | 2,591 (10.06%) | 11,742 (5.34%) |
| Gestational Hypertension | 1,832 (0.94%) | 489 (1.9%) | 2,321 (1.05%) |
| Gestational weight gain z-score | -0.5 (2.42) | -0.21 (2.48) | -0.46 (2.43) |

Gestational weight gain z-score was calculated according to [[39]](https://sciwheel.com/work/citation?ids=8196823&pre=&suf=&sa=0)

*Sisters CD percent was calculated as the percentage of CDs over all sister’s pregnancies which ended prior to the relevant date of birth.

| **Table S6.10 FDR corrected p-values for the estimated 10-yr childhood disease-free risk differences for comparing vaginal and cesarean deliveries - standardized, OW, IPW, across two data sources** | | | | | | |
| --- | --- | --- | --- | --- | --- | --- |
| **Full study population** | | | | | | |
| **Childhood health outcome** | **Israel cohort** | | | **UK cohort** | | |
|  | **10-yr risk difference P-value (FDR corrected)** | | | **10-yr risk difference P-value (FDR corrected)** | | |
|  |  |  |  |  |  |  |
|  | **Standardized** | **OW** | **IPW** | **Standardized** | **OW** | **IPW** |
|  |  |  |  |  |  |  |
| ADHD | 0.454 | 0.535 | 0.483 | 1 | 0.712 | 0.623 |
| Allergy | 0.514 | 1 | 1 | 0.008 | 0.042 | 0.104 |
| Asthma | 0.003 | 0.001 | 0.120 | 0.006 | 0 | 0 |
| Atopic dermatitis | 0.514 | 0.082 | 0.589 | 1 | 0.388 | 0.623 |
| Atopy | 0.214 | 0.022 | 0.537 | 0.531 | 0.108 | 0.104 |
| Autistic spectrum disorder | 0.140 | 0.082 | 0.140 | 1 | 1 | 1 |
| Autoimmune diseases | 1 | 1 | 0.537 | 0.501 | 0.388 | 0.411 |
| Celiac | 1 | 1 | 0.537 | 1 | 0.619 | 0.623 |
| Death | 0.098 | 0.082 | 0.537 | 1 | 1 | 0.892 |
| Inflammatory bowel disease | 1 | 1 | 0.537 | 0.531 | 0.388 | 0.623 |
| Juvenile idiopathic arthritis | 1 | 1 | 0.140 | - | - | - |
| Negative control (arm fracture) | 1 | 1 | 1 | 0.530 | 0.388 | 0.623 |
| Type 1 diabetes mellitus | 1 | 1 | 0.537 | 0 | 0.351 | 0.463 |

###

### 7. Elective CD classification model

#### Elective CD classification model parameters

We used a gradient boosting trees model trained with the XGBOOST python package. Hyperparameters were selected with the following settings:

- nuestimators= 50
- max_depth= 3

Missing values were not imputed prior to training the model. XGBOOST uses the method of *block propagation* in which tree splits are learned only from non-missing data, and only after that, the direction of splitting missing values is learned (by minimizing error) for the whole block of samples for missing values for that feature. Josse et al has shown that this procedure is a good option relative to various imputation methods [[49]](https://sciwheel.com/work/citation?ids=7217224&pre=&suf=&sa=0).

#### Elective CD classification model evaluation

| **A B C**  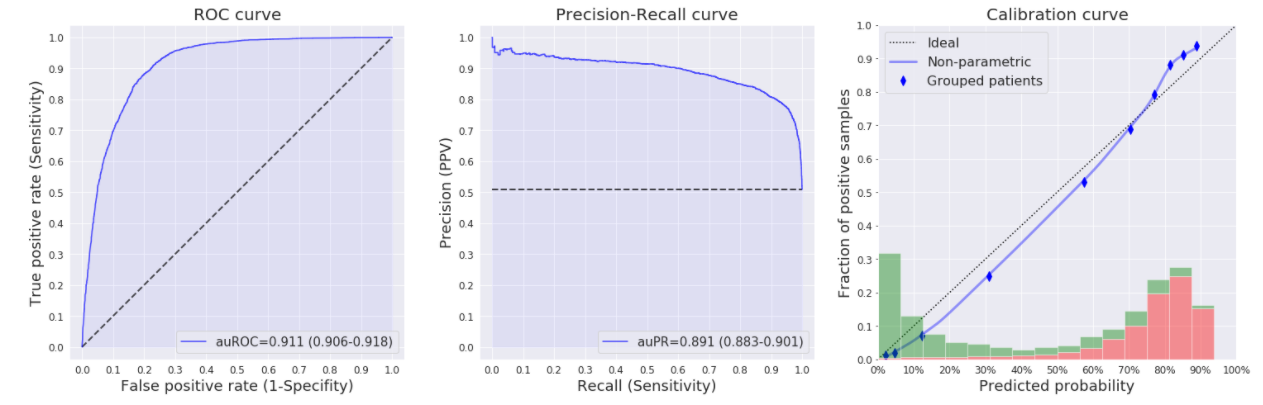  **D**  **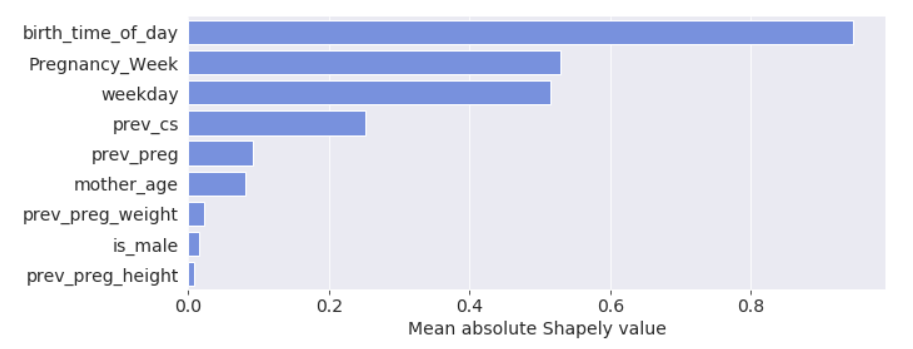**  **Figure S7.1. Elective CD classification model. A:** Receiver operating characteristic (ROC) curve of our model. **B:** Precision-Recall curve of our model. **C:** Calibration curve. Blue dots represent deciles of predicted probabilities. Dotted diagonal line represents an ideal calibration. Histogram at the bottom: predicted probabilities of children born in emergency CD (green) and elective CD (red). **D:** Mean absolute Shapley values (in log-odds scale) of the model features. |
| --- |

| 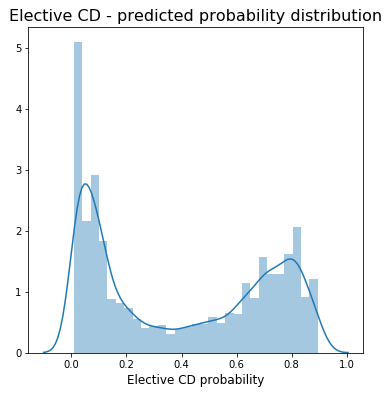  **Figure S7.2. Elective CD model in Clalit EHRs.** Elective CD probability distribution as predicted by the model on Clalit EHRs database. CDs with probability of 0.5 and higher were considered as elective. |
| --- |

###

###

### 8. DAGs for CD and pediatric health

| 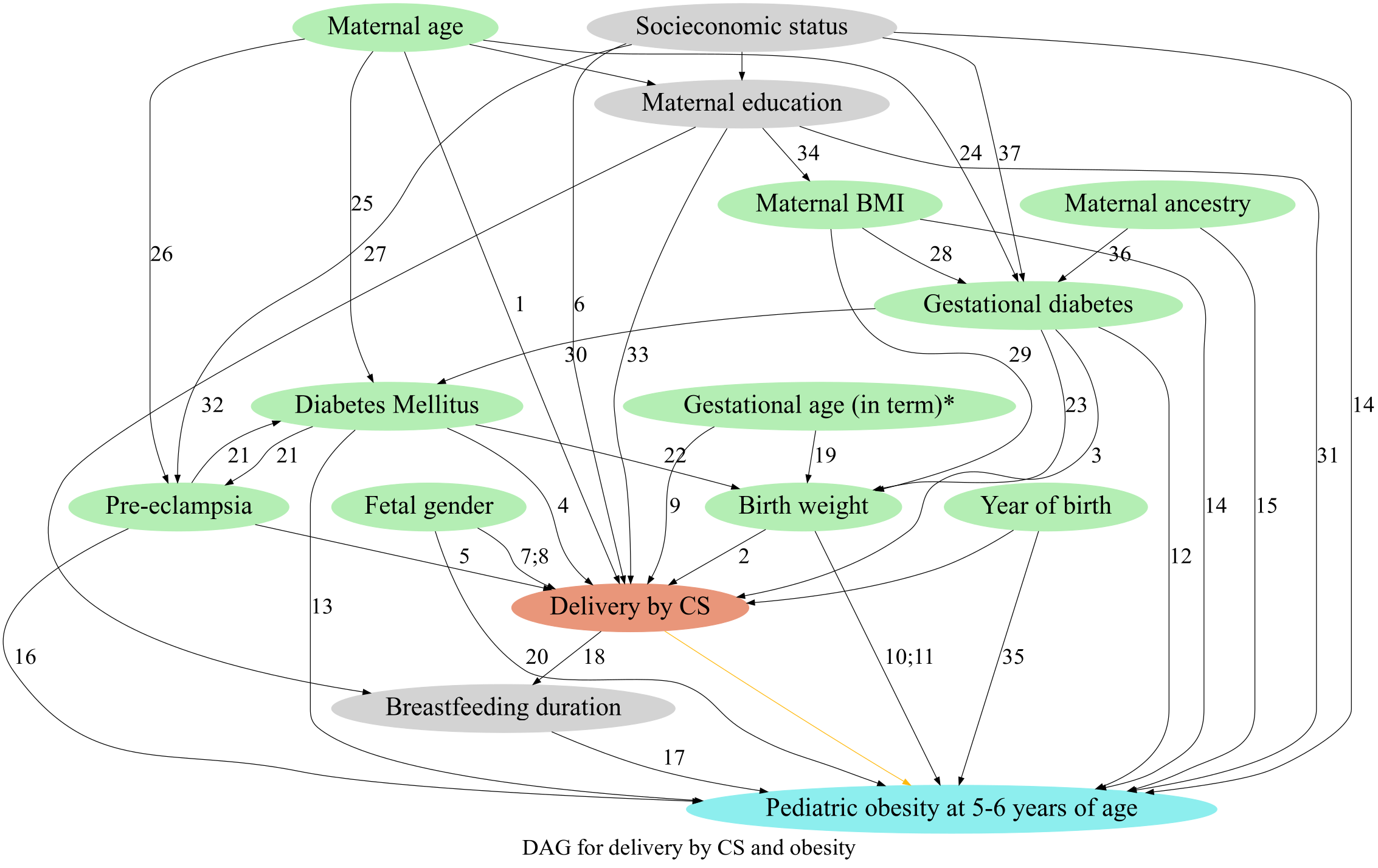  **Figure S8.1: Directed acyclic graph (DAG) for cesarean delivery and pediatric obesity at 5-6 years of age.**  DAG derived from literature and expert knowledge - nodes represent variables and arrows represent causal associations. Exposure, delivery by CS, colored in coral. Outcome, pediatric obesity at 5-6 years of age, colored in light blue. Variable nodes are colored in light green if they were available in our data, and light grey otherwise. *Our study includes only infants born in term delivery and therefore the factors that are associated with preterm birth are not accounted for. Numbers represent available sources of literature describing the associations, references for these associations are given in section 8 of the supplementary appendix. |
| --- |

### 8. References

1. Liang, J., Zhang, Z., Yang, W., Dai, M., Lin, L., & Chen, Y. et al. (2017). Association between Cesarean Section and Weight Status in Chinese Children and Adolescents: A National Survey. International Journal Of Environmental Research And Public Health, 14(12), 1609
2. Poma, P. (1999). Correlation of birth weights with cesarean rates. International Journal Of Gynecology & Obstetrics, 65(2), 117-123.
3. Remsberg, K., McKeown, R., McFarland, K., & Irwin, L. (1999). Diabetes in pregnancy and cesarean delivery. Diabetes Care, 22(9), 1561-1567.
4. Remsberg, K., McKeown, R., McFarland, K., & Irwin, L. (1999). Diabetes in pregnancy and cesarean delivery. Diabetes Care, 22(9), 1561-1567.
5. AM, B., SA, A., YS, K., A, B., F, S., TZ, A., & M, S. (2017). Cesarean Section: Incidence, Causes, Associated Factors and Outcomes: A National Prospective Study from Jordan. Gynecology & Obstetrics Case Report, 03(03).
6. Reza Omani-Samani, S. (2017). Cesarean Section and Socioeconomic Status in Tehran, Iran . Journal Of Research In Health Sciences, 17(4), 394.
7. Antonakou, A. (2016). The Effect of Fetal Gender on the Delivery Outcome in Primigravidae Women with Induced Labours for all Indications. JOURNAL OF CLINICAL AND DIAGNOSTIC RESEARCH.
8. Lieberman, E., Lang, J., Cohen, A., Frigoletto, F., Acker, D., & Rao, R. (1997). The association of fetal sex with the rate of cesarean section. American Journal Of Obstetrics And Gynecology, 176(3), 667-671.
9. MJ, O., & JA, M. (2013). Changes in cesarean delivery rates by gestational age: United States, 1996-2011. NCHS Data Brief, (124).
10. Casey, P., Bradley, R., Whiteside-Mansell, L., Barrett, K., Gossett, J., & Simpson, P. (2011). Evolution of obesity in a low birth weight cohort. Journal Of Perinatology, 32(2), 91-96.
11. Qiao, Y., Ma, J., Wang, Y., Li, W., Katzmarzyk, P., & Chaput, J. et al. (2015). Birth weight and childhood obesity: a 12-country study. International Journal Of Obesity Supplements,
12. Zhao, P., Liu, E., Qiao, Y., Katzmarzyk, P., Chaput, J., & Fogelholm, M. et al. (2016). Maternal gestational diabetes and childhood obesity at age 9–11: results of a multinational study. Diabetologia, 59(11), 2339-2348.
13. Lawlor, D., Lichtenstein, P., & Långström, N. (2011). Association of Maternal Diabetes Mellitus in Pregnancy With Offspring Adiposity Into Early Adulthood. Circulation, 123(3), 258-265.
14. Hernández-Valero, M., Wilkinson, A., Forman, M., Etzel, C., Cao, Y., & Bárcenas, C. et al. (2007). Maternal BMI and Country of Birth as Indicators of Childhood Obesity in Children of Mexican Origin**. Obesity, 15(10), 2512-2519.
15. Taveras, E., Gillman, M., Kleinman, K., Rich-Edwards, J., & Rifas-Shiman, S. (2010). Racial/Ethnic Differences in Early-Life Risk Factors for Childhood Obesity. PEDIATRICS, 125(4), 686-695.
16. Davis, E., Lazdam, M., Lewandowski, A., Worton, S., Kelly, B., & Kenworthy, Y. et al. (2012). Cardiovascular Risk Factors in Children and Young Adults Born to Preeclamptic Pregnancies: A Systematic Review. PEDIATRICS, 129(6), e1552-e1561.
17. Elliott, K., Kjolhede, C., Gournis, E., & Rasmussen, K. (1997). Duration of Breastfeeding Associated With Obesity During Adolescence. Obesity Research, 5(6), 538-541.
18. Hobbs, A., Mannion, C., McDonald, S., Brockway, M., & Tough, S. (2016). The impact of cesarean section on breastfeeding initiation, duration and difficulties in the first four months postpartum. BMC Pregnancy And Childbirth, 16(1).
19. Haksari, E., Lafeber, H., Hakimi, M., Pawirohartono, E., & Nyström, L. (2016). Reference curves of birth weight, length, and head circumference for gestational ages in Yogyakarta, Indonesia. BMC Pediatrics, 16(1).
20. Lo, J., Maring, B., Chandra, M., Daniels, S., Sinaiko, A., & Daley, M. et al. (2013). Prevalence of obesity and extreme obesity in children aged 3-5 years. Pediatric Obesity, 9(3), 167-175.
21. Weissgerber, T., & Mudd, L. (2015). Preeclampsia and Diabetes. Current Diabetes Reports, 15(3).
22. Yang, Y., Wang, Z., Mo, M., Muyiduli, X., Wang, S., & Li, M. et al. (2018). The association of gestational diabetes mellitus with fetal birth weight. Journal Of Diabetes And Its Complications, 32(7), 635-642.
23. KC, K., Shakya, S., & Zhang, H. (2015). Gestational Diabetes Mellitus and Macrosomia: A Literature Review. Annals Of Nutrition And Metabolism, 66(2), 14-20.
24. Li, Y., Ren, X., He, L., Li, J., Zhang, S., & Chen, W. (2020). Maternal age and the risk of gestational diabetes mellitus: A systematic review and meta-analysis of over 120 million participants. Diabetes Research And Clinical Practice, 162, 108044.
25. Lao, T., Ho, L., Chan, B., & Leung, W. (2006). Maternal Age and Prevalence of Gestational Diabetes Mellitus. Diabetes Care, 29(4), 948-949.
26. Lamminpää, R., Vehviläinen-Julkunen, K., Gissler, M., & Heinonen, S. (2012). Preeclampsia complicated by advanced maternal age: a registry-based study on primiparous women in Finland 1997–2008. BMC Pregnancy And Childbirth, 12(1).
27. How does the prevalence of preeclampsia vary among different age groups and races?. (2020). Retrieved 14 October 2020, from https://www.medscape.com/answers/261435-32626/how-does-the-prevalence-of-preeclampsia-vary-among-different-age-groups-and-races
28. Martino, J., Sebert, S., Segura, M., García-Valdés, L., Florido, J., & Padilla, M. et al. (2016). Maternal Body Weight and Gestational Diabetes Differentially Influence Placental and Pregnancy Outcomes. The Journal Of Clinical Endocrinology & Metabolism, 101(1), 59-68.
29. Mastella, L., Weinert, L., Gnielka, V., Hirakata, V., Oppermann, M., Silveiro, S., & Reichelt, A. (2018). Influence of maternal weight gain on birth weight: a gestational diabetes cohort. Archives Of Endocrinology And Metabolism, 62(1), 55-63.
30. Zhu, Y., & Zhang, C. (2016). Prevalence of Gestational Diabetes and Risk of Progression to Type 2 Diabetes: a Global Perspective. Current Diabetes Reports, 16(1).
31. Ruiz, M., Goldblatt, P., Morrison, J., Porta, D., Forastiere, F., & Hryhorczuk, D. et al. (2016). Impact of Low Maternal Education on Early Childhood Overweight and Obesity in Europe. Paediatric And Perinatal Epidemiology, 30(3), 274-284.
32. Saxton, J., Carnell, S., van Jaarsveld, C., & Wardle, J. (2009). Maternal Education Is Associated with Feeding Style. Journal Of The American Dietetic Association, 109(5), 894-898.
33. Tollånes, M., Thompson, J., Daltveit, A., & Irgens, L. (2007). Cesarean section and maternal education; secular trends in Norway, 1967–2004. Acta Obstetricia Et Gynecologica Scandinavica, 86(7), 840-848.
34. Ogden, C., Fakhouri, T., Carroll, M., Hales, C., Fryar, C., Li, X., & Freedman, D. (2017). Prevalence of Obesity Among Adults, by Household Income and Education — United States, 2011–2014. MMWR. Morbidity And Mortality Weekly Report, 66(50), 1369-1373.
35. Abarca-Gómez, L., Abdeen, Z., Hamid, Z., Abu-Rmeileh, N., Acosta-Cazares, B., & Acuin, C. et al. (2017). Worldwide trends in body-mass index, underweight, overweight, and obesity from 1975 to 2016: a pooled analysis of 2416 population-based measurement studies in 128·9 million children, adolescents, and adults. The Lancet, 390(10113), 2627-2642.
36. Yuen, L. (2015). Gestational diabetes mellitus: Challenges for different ethnic groups. World Journal Of Diabetes, 6(8), 1024.
37. Carroll, X., Liang, X., Zhang, W., Zhang, W., Liu, G., Turner, N., & Leeper-Woodford, S. (2018). Socioeconomic, environmental and lifestyle factors associated with gestational diabetes mellitus: A matched case-control study in Beijing, China. Scientific Reports, 8(1).
38. [63. Tollånes MC, Rasmussen S, Irgens LM. Caesarean section among relatives. Int J Epidemiol. 2008;37:1341–8.](https://sciwheel.com/work/bibliography/8137919)
39. [64. Hutcheon JA, Platt RW, Abrams B, Himes KP, Simhan HN, Bodnar LM. A weight-gain-for-gestational-age z score chart for the assessment of maternal weight gain in pregnancy. Am J Clin Nutr. 2013;97:1062–7.](https://sciwheel.com/work/bibliography/8196823)
40. [65. Nissen F, Quint JK, Wilkinson S, Mullerova H, Smeeth L, Douglas IJ. Validation of asthma recording in electronic health records: a systematic review. Clin Epidemiol. 2017;9:643–56.](https://sciwheel.com/work/bibliography/7761789)
41. [66. Rifas-Shiman SL, Gillman MW, Oken E, Kleinman K, Taveras EM. Similarity of the CDC and WHO weight-for-length growth charts in predicting risk of obesity at age 5 years. Obesity (Silver Spring). 2012;20:1261–5.](https://sciwheel.com/work/bibliography/6652734)
42. [67. Mukherjee M, Wyatt JC, Simpson CR, Sheikh A. Usage of allergy codes in primary care electronic health records: a national evaluation in Scotland. Allergy. 2016;71:1594–602.](https://sciwheel.com/work/bibliography/7783245)
43. [68. Hirsch AG, Pollak J, Glass TA, Poulsen MN, Bailey-Davis L, Mowery J, et al. Early-life antibiotic use and subsequent diagnosis of food allergy and allergic diseases. Clin Exp Allergy. 2017;47:236–44.](https://sciwheel.com/work/bibliography/3889195)
44. [69. Lingren T, Chen P, Bochenek J, Doshi-Velez F, Manning-Courtney P, Bickel J, et al. Electronic Health Record Based Algorithm to Identify Patients with Autism Spectrum Disorder. PLoS ONE. 2016;11:e0159621.](https://sciwheel.com/work/bibliography/2989354)
45. [70. Elder TE. The importance of relative standards in ADHD diagnoses: evidence based on exact birth dates. J Health Econ. 2010;29:641–56.](https://sciwheel.com/work/bibliography/4519071)
46. [71. Karlstad Ø, Zoëga H, Furu K, Bahmanyar S, Martikainen JE, Kieler H, et al. Use of drugs for ADHD among adults-a multinational study among 15.8 million adults in the Nordic countries. Eur J Clin Pharmacol. 2016;72:1507–14.](https://sciwheel.com/work/bibliography/3447956)
47. [72. Upadhyaya SG, Murphree DH, Ngufor CG, Knight AM, Cronk DJ, Cima RR, et al. Automated diabetes case identification using electronic health record data at a tertiary care facility. Mayo Clin Proc Innov Qual Outcomes. 2017;1:100–10.](https://sciwheel.com/work/bibliography/7729773)
48. [73. Linder JA, Bates DW, Williams DH, Connolly MA, Middleton B. Acute infections in primary care: accuracy of electronic diagnoses and electronic antibiotic prescribing. J Am Med Inform Assoc. 2006;13:61–6.](https://sciwheel.com/work/bibliography/7783755)
49. [74. Josse J, Prost N, Scornet E, Varoquaux G. On the consistency of supervised learning with missing values. arXiv. 2019;](https://sciwheel.com/work/bibliography/7217224)
